# Neuromuscular disease genetics in under-represented populations: increasing data diversity

**DOI:** 10.1093/brain/awad254

**Published:** 2023-07-30

**Authors:** Lindsay A Wilson, William L Macken, Luke D Perry, Christopher J Record, Katherine R Schon, Rodrigo S S Frezatti, Sharika Raga, Kireshnee Naidu, Özlem Yayıcı Köken, Ipek Polat, Musambo M Kapapa, Natalia Dominik, Stephanie Efthymiou, Heba Morsy, Melissa Nel, Mahmoud R Fassad, Fei Gao, Krutik Patel, Maryke Schoonen, Michelle Bisschoff, Armand Vorster, Hallgeir Jonvik, Ronel Human, Elsa Lubbe, Malebo Nonyane, Seena Vengalil, Saraswati Nashi, Kosha Srivastava, Richard J L F Lemmers, Alisha Reyaz, Rinkle Mishra, Ana Töpf, Christina I Trainor, Elizabeth C Steyn, Amokelani C Mahungu, Patrick J van der Vliet, Ahmet Cevdet Ceylan, A Semra Hiz, Büşranur Çavdarlı, C Nur Semerci Gündüz, Gülay Güleç Ceylan, Madhu Nagappa, Karthik B Tallapaka, Periyasamy Govindaraj, Silvère M van der Maarel, Gayathri Narayanappa, Bevinahalli N Nandeesh, Somwe Wa Somwe, David R Bearden, Michelle P Kvalsund, Gita M Ramdharry, Yavuz Oktay, Uluç Yiş, Haluk Topaloğlu, Anna Sarkozy, Enrico Bugiardini, Franclo Henning, Jo M Wilmshurst, Jeannine M Heckmann, Robert McFarland, Robert W Taylor, Izelle Smuts, Francois H van der Westhuizen, Claudia Ferreira da Rosa Sobreira, Pedro J Tomaselli, Wilson Marques, Rohit Bhatia, Ashwin Dalal, M V Padma Srivastava, Sireesha Yareeda, Atchayaram Nalini, Venugopalan Y Vishnu, Kumarasamy Thangaraj, Volker Straub, Rita Horvath, Patrick F Chinnery, Robert D S Pitceathly, Francesco Muntoni, Henry Houlden, Jana Vandrovcova, Mary M Reilly, Michael G Hanna

**Affiliations:** Department of Neuromuscular Diseases, UCL Queen Square Institute of Neurology and The National Hospital for Neurology and Neurosurgery, London WC1N 3BG, UK; Department of Neuromuscular Diseases, UCL Queen Square Institute of Neurology and The National Hospital for Neurology and Neurosurgery, London WC1N 3BG, UK; Institute of Child Health and Centre for Neuromuscular Diseases, Neurosciences Unit, The Dubowitz Neuromuscular Centre, University College London, UCL Great Ormond Street, Great Ormond Street Hospital, London WC1N 3JH, UK; NIHR Great Ormond Street Hospital Biomedical Research Centre, UCL Great Ormond Street Institute of Child Health, University College London, London WC1N 1EH, UK; Department of Neuromuscular Diseases, UCL Queen Square Institute of Neurology and The National Hospital for Neurology and Neurosurgery, London WC1N 3BG, UK; Department of Clinical Neurosciences, University of Cambridge, Cambridge CB2 0QQ, UK; Department of Neurosciences, School of Medicine of Ribeirão Preto, University of São Paulo, São Paulo, Brazil; Neuroscience Institute, University of Cape Town, Cape Town, South Africa; Division of Paediatric Neurology, Department of Paediatrics and Child Health, Red Cross War Memorial Children’s Hospital, Cape Town, South Africa; Neurology Research Group, Division of Neurology, Department of Medicine, University of Cape Town, Cape Town, South Africa; Division of Neurology, Department of Medicine, Stellenbosch University, Cape Town, South Africa; Faculty of Medicine, Department of Pediatric Neurology, Akdeniz University, Antalya, Turkey; Faculty of Medicine, Pediatric Neurology Department, Dokuz Eylül University, Izmir, Turkey; Izmir International Biomedicine and Genome Institute, Dokuz Eylül University, Izmir, Turkey; Department of Physiotherapy, University of Zambia School of Health Sciences & University Teaching Hospital Neurology Research Office, Lusaka, Zambia; Department of Neuromuscular Diseases, UCL Queen Square Institute of Neurology and The National Hospital for Neurology and Neurosurgery, London WC1N 3BG, UK; Department of Neuromuscular Diseases, UCL Queen Square Institute of Neurology and The National Hospital for Neurology and Neurosurgery, London WC1N 3BG, UK; Department of Neuromuscular Diseases, UCL Queen Square Institute of Neurology and The National Hospital for Neurology and Neurosurgery, London WC1N 3BG, UK; Neuroscience Institute, University of Cape Town, Cape Town, South Africa; Neurology Research Group, Division of Neurology, Department of Medicine, University of Cape Town, Cape Town, South Africa; Wellcome Centre for Mitochondrial Research, Translational and Clinical Research Institute, Faculty of Medical Sciences, Newcastle University, Newcastle upon Tyne NE2 4HH, UK; Department of Clinical Neurosciences, University of Cambridge, Cambridge CB2 0QQ, UK; Wellcome Centre for Mitochondrial Research, Translational and Clinical Research Institute, Faculty of Medical Sciences, Newcastle University, Newcastle upon Tyne NE2 4HH, UK; Focus Area for Human Metabolomics, North-West University, Potchefstroom, South Africa; Focus Area for Human Metabolomics, North-West University, Potchefstroom, South Africa; Focus Area for Human Metabolomics, North-West University, Potchefstroom, South Africa; Department of Neuromuscular Diseases, UCL Queen Square Institute of Neurology and The National Hospital for Neurology and Neurosurgery, London WC1N 3BG, UK; Department of Paediatrics, Steve Biko Academic Hospital, University of Pretoria, Pretoria, South Africa; Department of Paediatrics, Steve Biko Academic Hospital, University of Pretoria, Pretoria, South Africa; Department of Paediatrics, Steve Biko Academic Hospital, University of Pretoria, Pretoria, South Africa; Department of Neurology, National Institute of Mental Health and Neurosciences (NIMHANS), Bengaluru, India; Department of Neurology, National Institute of Mental Health and Neurosciences (NIMHANS), Bengaluru, India; Department of Neurology, National Institute of Mental Health and Neurosciences (NIMHANS), Bengaluru, India; Department of Human Genetics, Leiden University Medical Center (LUMC), Leiden, The Netherlands; Department of Neurology, All India Institute of Medical Sciences (AIIMS), Delhi, India; Department of Neurology, All India Institute of Medical Sciences (AIIMS), Delhi, India; John Walton Muscular Dystrophy Research Centre, Newcastle University Translational and Clinical Research Institute and Newcastle Hospitals NHS Foundation Trust, Newcastle upon Tyne, UK; John Walton Muscular Dystrophy Research Centre, Newcastle University Translational and Clinical Research Institute and Newcastle Hospitals NHS Foundation Trust, Newcastle upon Tyne, UK; Neurology Research Group, Division of Neurology, Department of Medicine, University of Cape Town, Cape Town, South Africa; Neuroscience Institute, University of Cape Town, Cape Town, South Africa; Neurology Research Group, Division of Neurology, Department of Medicine, University of Cape Town, Cape Town, South Africa; Department of Human Genetics, Leiden University Medical Center (LUMC), Leiden, The Netherlands; Department of Medical Genetics, Ankara Bilkent City Hospital, Ankara, Turkey; Faculty of Medicine, Department of Medical Genetics, Ankara Yıldırım Beyazıt University, Ankara, Turkey; Faculty of Medicine, Pediatric Neurology Department, Dokuz Eylül University, Izmir, Turkey; Izmir Biomedicine and Genome Center (IBG), Izmir, Turkey; Department of Medical Genetics, Ankara Bilkent City Hospital, Ankara, Turkey; Department of Medical Genetics, Ankara Bilkent City Hospital, Ankara, Turkey; Faculty of Medicine, Department of Medical Genetics, Ankara Yıldırım Beyazıt University, Ankara, Turkey; Department of Medical Genetics, Ankara Bilkent City Hospital, Ankara, Turkey; Faculty of Medicine, Department of Medical Genetics, Ankara Yıldırım Beyazıt University, Ankara, Turkey; Department of Neurology, National Institute of Mental Health and Neurosciences (NIMHANS), Bengaluru, India; CSIR—Centre for Cellular and Molecular Biology (CCMB), Hyderabad, Telangana, India; Diagnostics Division, Centre for DNA Fingerprinting and Diagnostics, Hyderabad, Telangana, India; Department of Human Genetics, Leiden University Medical Center (LUMC), Leiden, The Netherlands; Department of Neuropathology, National Institute of Mental Health and Neurosciences (NIMHANS), Bengaluru, India; Department of Neuropathology, National Institute of Mental Health and Neurosciences (NIMHANS), Bengaluru, India; Department of Clinical Sciences, School of Medicine and Health Sciences, University of Lusaka, Lusaka, Zambia; University of Zambia Department of Educational Psychology, Lusaka, Zambia; Department of Neurology, School of Medicine and Dentistry, University of Rochester Medical Center, Rochester, NY 14642, USA; Department of Neurology, School of Medicine and Dentistry, University of Rochester Medical Center, Rochester, NY 14642, USA; Department of Internal Medicine, University of Zambia School of Medicine, Lusaka, Zambia; Department of Neuromuscular Diseases, UCL Queen Square Institute of Neurology and The National Hospital for Neurology and Neurosurgery, London WC1N 3BG, UK; Izmir International Biomedicine and Genome Institute, Dokuz Eylül University, Izmir, Turkey; Izmir Biomedicine and Genome Center (IBG), Izmir, Turkey; Faculty of Medicine, Pediatric Neurology Department, Dokuz Eylül University, Izmir, Turkey; Yeditepe University Hospitals, Istanbul, Turkey; NIHR Great Ormond Street Hospital Biomedical Research Centre, UCL Great Ormond Street Institute of Child Health, University College London, London WC1N 1EH, UK; Department of Neuromuscular Diseases, UCL Queen Square Institute of Neurology and The National Hospital for Neurology and Neurosurgery, London WC1N 3BG, UK; Division of Neurology, Department of Medicine, Stellenbosch University, Cape Town, South Africa; Neuroscience Institute, University of Cape Town, Cape Town, South Africa; Division of Paediatric Neurology, Department of Paediatrics and Child Health, Red Cross War Memorial Children’s Hospital, Cape Town, South Africa; Neuroscience Institute, University of Cape Town, Cape Town, South Africa; Neurology Research Group, Division of Neurology, Department of Medicine, University of Cape Town, Cape Town, South Africa; Wellcome Centre for Mitochondrial Research, Translational and Clinical Research Institute, Faculty of Medical Sciences, Newcastle University, Newcastle upon Tyne NE2 4HH, UK; NHS Highly Specialised Service for Rare Mitochondrial Disorders, Newcastle upon Tyne Hospitals NHS Foundation Trust, Newcastle upon Tyne NE1 4LP, UK; Wellcome Centre for Mitochondrial Research, Translational and Clinical Research Institute, Faculty of Medical Sciences, Newcastle University, Newcastle upon Tyne NE2 4HH, UK; NHS Highly Specialised Service for Rare Mitochondrial Disorders, Newcastle upon Tyne Hospitals NHS Foundation Trust, Newcastle upon Tyne NE1 4LP, UK; Department of Paediatrics, Steve Biko Academic Hospital, University of Pretoria, Pretoria, South Africa; Focus Area for Human Metabolomics, North-West University, Potchefstroom, South Africa; Department of Neurosciences, School of Medicine of Ribeirão Preto, University of São Paulo, São Paulo, Brazil; Department of Neurosciences, School of Medicine of Ribeirão Preto, University of São Paulo, São Paulo, Brazil; Department of Neurosciences, School of Medicine of Ribeirão Preto, University of São Paulo, São Paulo, Brazil; Department of Neurology, All India Institute of Medical Sciences (AIIMS), Delhi, India; Diagnostics Division, Centre for DNA Fingerprinting and Diagnostics, Hyderabad, Telangana, India; Department of Neurology, All India Institute of Medical Sciences (AIIMS), Delhi, India; Department of Neurology, Nizam’s Institute of Medical Sciences (NIMS), Hyderabad, Telangana, India; Department of Neurology, National Institute of Mental Health and Neurosciences (NIMHANS), Bengaluru, India; Department of Neurology, All India Institute of Medical Sciences (AIIMS), Delhi, India; CSIR—Centre for Cellular and Molecular Biology (CCMB), Hyderabad, Telangana, India; John Walton Muscular Dystrophy Research Centre, Newcastle University Translational and Clinical Research Institute and Newcastle Hospitals NHS Foundation Trust, Newcastle upon Tyne, UK; Department of Clinical Neurosciences, University of Cambridge, Cambridge CB2 0QQ, UK; Department of Clinical Neurosciences, University of Cambridge, Cambridge CB2 0QQ, UK; Department of Neuromuscular Diseases, UCL Queen Square Institute of Neurology and The National Hospital for Neurology and Neurosurgery, London WC1N 3BG, UK; Institute of Child Health and Centre for Neuromuscular Diseases, Neurosciences Unit, The Dubowitz Neuromuscular Centre, University College London, UCL Great Ormond Street, Great Ormond Street Hospital, London WC1N 3JH, UK; NIHR Great Ormond Street Hospital Biomedical Research Centre, UCL Great Ormond Street Institute of Child Health, University College London, London WC1N 1EH, UK; Department of Neuromuscular Diseases, UCL Queen Square Institute of Neurology and The National Hospital for Neurology and Neurosurgery, London WC1N 3BG, UK; Department of Neuromuscular Diseases, UCL Queen Square Institute of Neurology and The National Hospital for Neurology and Neurosurgery, London WC1N 3BG, UK; Department of Neuromuscular Diseases, UCL Queen Square Institute of Neurology and The National Hospital for Neurology and Neurosurgery, London WC1N 3BG, UK; Department of Neuromuscular Diseases, UCL Queen Square Institute of Neurology and The National Hospital for Neurology and Neurosurgery, London WC1N 3BG, UK

**Keywords:** genomic medicine, inherited neuromuscular disease, capacity building, low-to-middle income country, equality and diversity

## Abstract

Neuromuscular diseases (NMDs) affect ∼15 million people globally. In high income settings DNA-based diagnosis has transformed care pathways and led to gene-specific therapies. However, most affected families are in low-to-middle income countries (LMICs) with limited access to DNA-based diagnosis. Most (86%) published genetic data is derived from European ancestry. This marked genetic data inequality hampers understanding of genetic diversity and hinders accurate genetic diagnosis in all income settings. We developed a cloud-based transcontinental partnership to build diverse, deeply-phenotyped and genetically characterized cohorts to improve genetic architecture knowledge, and potentially advance diagnosis and clinical management.

We connected 18 centres in Brazil, India, South Africa, Turkey, Zambia, Netherlands and the UK. We co-developed a cloud-based data solution and trained 17 international neurology fellows in clinical genomic data interpretation. Single gene and whole exome data were analysed via a bespoke bioinformatics pipeline and reviewed alongside clinical and phenotypic data in global webinars to inform genetic outcome decisions.

We recruited 6001 participants in the first 43 months. Initial genetic analyses ‘solved’ or ‘possibly solved’ ∼56% probands overall. In-depth genetic data review of the four commonest clinical categories (limb girdle muscular dystrophy, inherited peripheral neuropathies, congenital myopathy/muscular dystrophies and Duchenne/Becker muscular dystrophy) delivered a ∼59% ‘solved’ and ∼13% ‘possibly solved’ outcome. Almost 29% of disease causing variants were novel, increasing diverse pathogenic variant knowledge. Unsolved participants represent a new discovery cohort. The dataset provides a large resource from under-represented populations for genetic and translational research.

In conclusion, we established a remote transcontinental partnership to assess genetic architecture of NMDs across diverse populations. It supported DNA-based diagnosis, potentially enabling genetic counselling, care pathways and eligibility for gene-specific trials. Similar virtual partnerships could be adopted by other areas of global genomic neurological practice to reduce genetic data inequality and benefit patients globally.

## Teaser

Wilson *et al.* present the findings of an international partnership established to study genetic causes of neuromuscular diseases in under-represented diverse populations from 12 low–middle income sites. A genetic cause was identified in ∼55% of cases and ∼30% of variants were novel, improving understanding of neuromuscular disease genetics.

## Introduction

Neuromuscular diseases (NMD) affect an estimated 15 million children and adults globally.^1^ They cause shortened life expectancy or chronic lifelong disability with personal and economic impact. Although individually rare, collectively they account for approximately ∼20% of all non-infectious neurological diseases. In low-to-middle income countries (LMICs), NMD prevalence and incidence are under-reported, as diagnosis is limited to a few specialist centres which may be geographically distant from much of the eligible population.

In high-income settings, improved diagnostics, especially genetic analysis, have delivered important advances in patient care pathways. Many interventions enabled by an accurate DNA-based diagnosis are relatively inexpensive, including genetic counselling, tailored application of widely-available medicines, screening for known complications (e.g. cardiac, respiratory, gastroenterological, metabolic) and physiotherapy. Genetic advances have also led to new advanced therapies, for example RNA targeting approaches (for spinal muscular atrophy, SMA) and AAV-mediated gene therapies or trials for SMA and Duchenne’s muscular dystrophy (DMD).^2,3^

A key challenge to realizing DNA-based diagnostic benefits for patients in LMICs is that ∼86% of published genomic studies are derived from populations of primarily European ancestry, and non-European populations are under-represented in control databases.^4^ Knowledge of genetic diversity outside European populations is limited.^5-7^ Improved understanding of NMD distribution and associated phenotypic and genetic variability requires a large, diverse, accurately-phenotyped cohort of patients and families with linked genetic data. Additional cohort benefits include generating allele frequency data for under-represented populations to aid variant classification, and potential to connect participants to clinical trials and novel therapies.

Here we describe our approach and genetic results in setting up a NMD genomic medicine partnership across 18 centres spanning seven countries.

## Materials and methods

### Structure of the International Centre for Genomic Medicine in Neuromuscular Diseases

The International Centre for Genomic Medicine in Neuromuscular Diseases (ICGNMD) was launched in June 2019 and is ongoing. Partner sites (Supplementary Table 1) established aligned, locally-approved studies to recruit NMD patients and relatives to an international cohort and share materials and data in full compliance with all ethics and legislation (for further details about ethics and data storage and sharing see the Supplementary material). The inclusion criterion for participants was a suspected inherited neuromuscular disease clinically diagnosed by a trained clinical neurologist, or being a close relative. Participants with a local genetic diagnosis could be included, or unsolved local whole exome sequencing (WES) data reanalysed. UK partner sites may also recruit participants (typically with existing genetic test data) living in the UK; however, this paper considers only participant data from low-to-middle income partner sites.

The international regulatory and ethics landscape is complex and securing all regulatory and ethical approvals to balance data and material accessibility with patient rights was highly challenging and required the nuanced input of experienced local teams.

Building future genomics medicine capacity was crucial to all partners realising precision medicine benefits. Therefore, 17 fellows were appointed to support recruitment, data entry and results interpretation. Twelve were based in LMICs and all are pursuing their careers locally. Fellows were assigned one LMIC Principal Investigator (PI) and one UK PI for mentorship and capacity building; supervision ran alongside regular remote training and data interpretation and was considered highly effective by both fellows and PIs.

In-person face-to-face study induction training of all neurology fellows focused on standardized data entry to the ICGNMD REDCap database,^8^ including Human Phenotype Ontology (HPO) terms,^9^ standard clinical assessment scales and summary genetic data (for database instrument, see the Supplementary material), followed by regular online refresher training. As not all sites could access all investigations (e.g. MRI often unavailable), the only mandatory data entries were: proband or relationship to proband, diagnostic category (provisional clinical diagnosis), sex, age at recruitment and positive and negative HPO terms. Repeat measurements (e.g. blood creatine kinase) and progression indicators could be recorded but the study was primarily cross-sectional, reflecting challenges of re-evaluating participants who may struggle to travel to clinics.

### ICGNMD genetic analysis and data report generation

**Figure 1:**
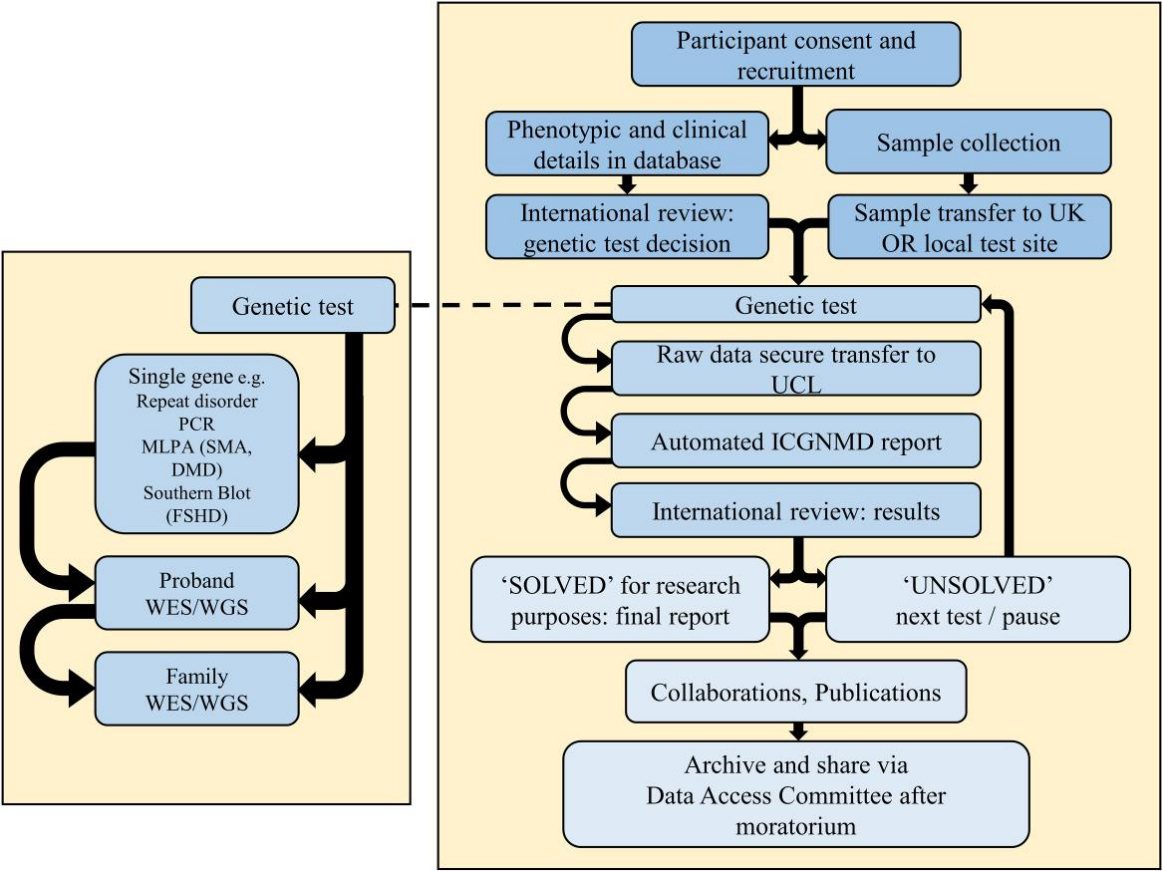
**ICGNMD workflow with key nodes for international discussion at genetic analysis decision and results review**. ICGNMD Fellows’ training spans this pathway. DMD = Duchenne muscular dystrophy; FSHD = facioscapulohumeral muscular dystrophy; MLPA = multiplex ligation dependent probe analysis; SMA = spinal muscular atrophy; WES = whole exome sequencing; WGS = whole genome sequencing.

After consent and data collection, international, remote expert group ‘genetic analysis decision’ meetings discussed the most appropriate initial genetic analysis. These meetings, attended by all PIs, also served as a Fellows’ training forum. Clinical phenotype and any investigational data underpinned the decision to apply specific single gene tests (e.g. MLPA for SMA) or WES (Supplementary material), with optional genotyping arrays to detect large structural or copy number variation and/or for linkage analysis. Testing was typically proband first, and extended to relatives if needed and/or available (Fig. 1). Partners in Brazil, South Africa and Zambia sent DNA to the UK; however, partners in India and Turkey generated pseudonymized raw data to agreed standards and shared this for centralized analysis.

Single gene test (SGT) results were reviewed by trained staff at ICGNMD partner sites. Raw WES data were analysed via a common bioinformatics pipeline (Supplementary material), with ICGNMD Fellows supported to interpret and present genetic results. WES candidate variant prioritization followed a modified protocol developed by the 100 000 Genomes Project.^10^ Initial analysis focused on variants with a minor allele frequency of of <0.01 (autosomal recessive inheritance) and <0.001 (autosomal dominant inheritance) within a subset of genes present in expertly reviewed gene panels (https://panelapp.genomicsengland.co.uk/) aligning to the participant HPO terms and phenotype. If no significant genetic variants were reported, extended pipeline analyses (structural and copy number, mitochondrial, repeat expansion, *de novo*) were applied. To maximize reproducibility, open-source and well maintained software tools and databases were implemented.^11,12^ Given their relatively limited non-European ancestry data and lack of subpopulation resolution, e.g. for specific Indian and African ethnicities, we supplemented large-scale population data resources including gnomAD^13^ with additional allele frequency data generated in local populations ^14-18^ and the growing ICGNMD in-house dataset.

Prioritized variants were reviewed, and potentially causative variants were classified using American College of Medical Genetics (ACMG) criteria.^19^ The outcome was classed as ‘solved’ where pathogenic/likely pathogenic variant(s) were identified and fitted with the phenotype (two variants/homozygous in recessive disorders). The outcome was classed as ‘possibly solved’ if there was a strong candidate variant (two variants/homozygous recessive disorders) based on population frequency (<0.01% frequency), bioinformatic predictions and clinical phenotype, but at least one variant was classified as a variant of uncertain significance (VUS) according to ACMG criteria. Where further manual curation was performed for subgroups of disease categories, rare variants considered relevant for each proband were reviewed against gnomAD, ClinVar, DECIPHER, VarSome, PubMed and Google to ascertain if previously reported. Variants were classified as ‘novel’ if absent from all these sources.

## Results

Despite the SARS-CoV-2 pandemic, we established a phenotyping, genetic analysis and data sharing platform connecting centres across Brazil, India, Netherlands, South Africa, Turkey, UK and Zambia. We developed remote training and global webinars to discuss participants and supported decision-making for genetic analysis and result interpretation. As of January 2023, 6001 participants (including 3631 probands) had consented and provided DNA (Supplementary Table 2). The majority were in India (3578 participants, 60% of the total), followed by Brazil (979 participants, 16%), South Africa (737 participants, 12%), Turkey (578 participants, 10%) and Zambia (129 participants, 2%). The cohort included 337 (9% probands) participants ‘locally genetically solved’ or with existing genetic data to review at study start. The majority (3294, 91%) of participants had no previous genetic test.

Sixty-five per cent of participants were male, 35% female. The median age at proband recruitment was 26 years of age with 35% of the cohort aged 18 or under (Supplementary Fig. 1). Using 1000 Genomes populations as a background for ancestry estimation, 82% of individuals tested by WES were of non-European ancestry (Supplementary Fig. 2). Based on current recruitment, we estimate cohort size will exceed 10 000 at Year 5 end; June 2024.

### Phenotypic spectrum

**Figure 2:**
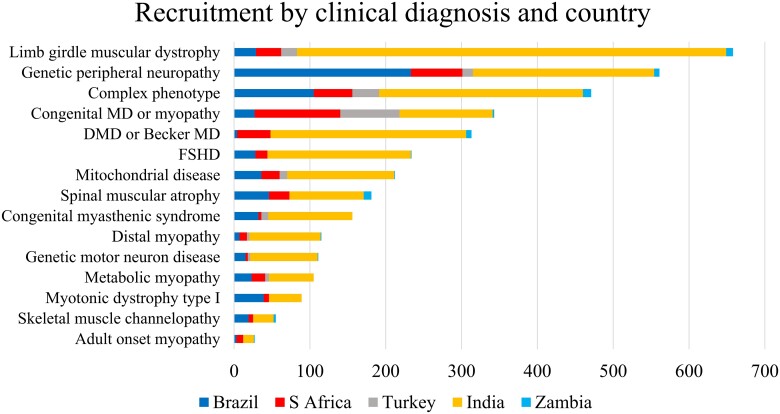
**Recruitment across clinical diagnostic categories in REDCap by country of recruitment**. Blue = Brazil; grey = Turkey; light blue = Zambia; red = South Africa; yellow = India. DMD = Duchenne muscular dystrophy; FSHD = facioscapulohumeral muscular dystrophy; MD = muscular dystrophy.

We recruited a people with a broad range of NMDs (Fig. 2 and Supplementary Table 3). Total recruitment to mid-January 2023 by initial clinical diagnosis included 18.1% limb girdle muscular dystrophy (LGMD), 15.5% genetic peripheral neuropathies (PN), 9.4% congenital myopathy or congenital muscular dystrophy (CM/CMD) and 8.6% Duchenne muscular dystrophy or Becker muscular dystrophy (DMD/BMD). Other categories each contributed less than 7%. The four most common NMD categories were in line with those reported by centres worldwide.^1^

### Genetic data results

We report 978 new genetic analyses (including 547 proband WES and 274 proband SGTs) by January 2023, following the process in Fig. 1. Fifty per cent of probands receiving a SGT were solved (Supplementary Table 4). The first proband WES data review identified 223 (41%) variants to ‘solve’ and 83 (15%) variants to ‘possibly solve’ participants’ NMDs (combined WES solved/possibly solved rate 56%). Single gene tests and WES combined yielded 43.8% ‘solved’ outcomes. Below are genetic summary data following in-depth review of proband single gene test and WES results for the four clinical diagnostic categories with highest recruitment levels (PN, LGMD, CMD/CM and DMD/BMD) to demonstrate project value at Year 4 stage. These categories combined represent 1875 of 3631 study probands (51.6% of cohort) and 340 of 547 (62%) exomes available in January 2023, plus 182 single gene tests.

### Genetic peripheral neuropathies

**Figure 3:**
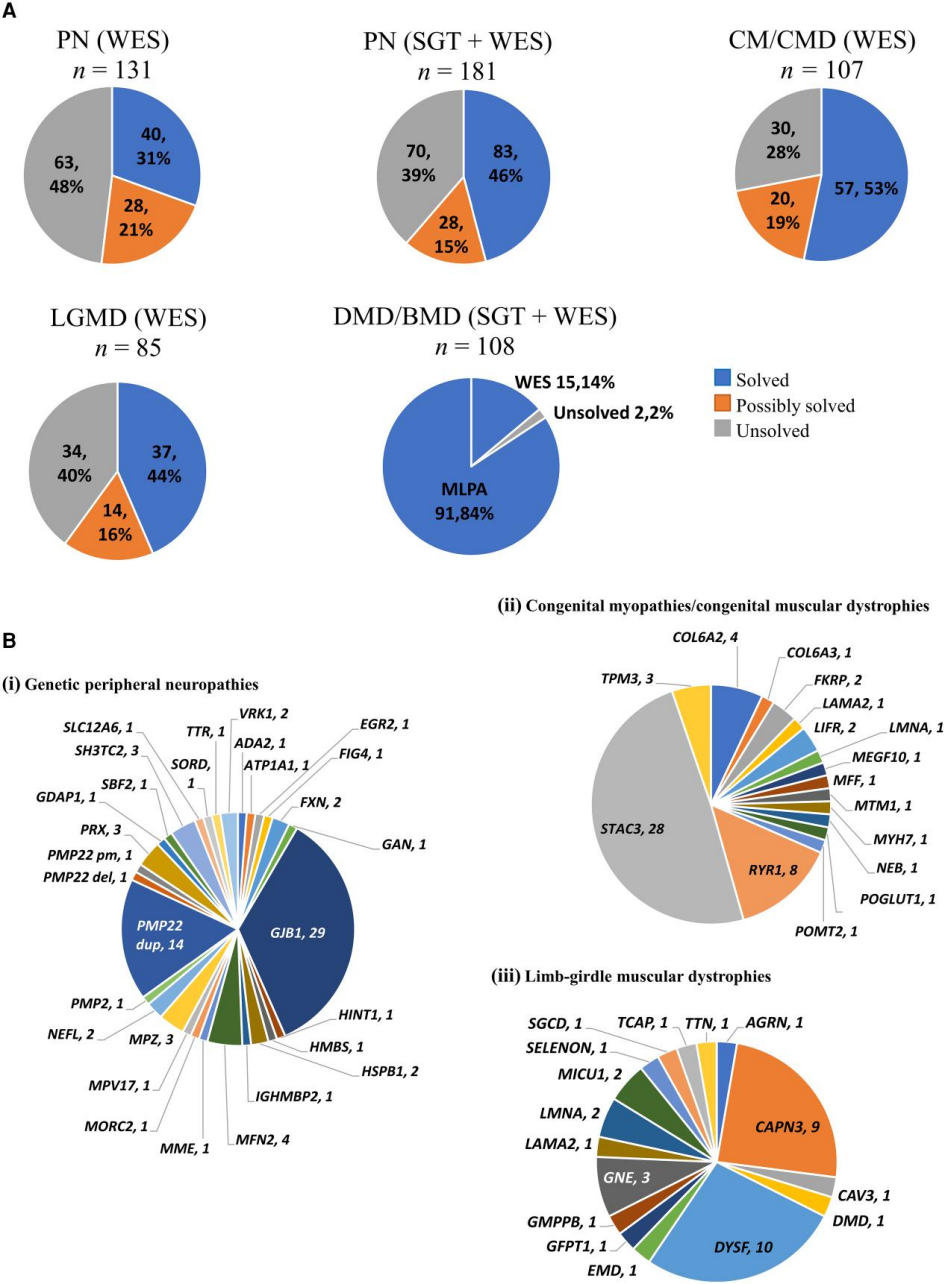
**Genetic analysis outcomes**. (**A**) Outcome in peripheral neuropathies (PN), limb girdle muscular dystrophy (LGMD), congenital myopathy/congenital muscular dystrophy (CM/CMD) and Duchenne muscular dystrophy/Becker muscular dystrophy (DMD/BMD) cohorts. (**B**) Genetic composition of (**i**) genetic PN; (**ii**) LGMD; and (**iii**) CM/CMD ‘solved’ cohorts showing the number of probands with variants detected in each named gene. *PMP22* del = *PMP22* gene deletion; *PMP22* dup = *PMP22* duplication; *PMP22* pm = *PMP22* point mutation.

One hundred and eighty-one participants with a presumed genetic peripheral neuropathy were analysed (114 Brazilian, 16 Indian, 51 South African). Single gene tests in 80 probands had a diagnostic yield of 54% (43/80) [*GJB1* (26 participants; 32.5%), *PMP22* duplication (14 participants; 17.5%), *FXN* (two participants; 2.5%), *PMP22* deletion for HNPP genetic diagnosis (one participant; 1.25%)]. All but seven probands with a negative SGT had WES afterwards. WES in 131 probands yielded 40 (31%) solved, 28 (21%) possibly solved and 63 (48%) unsolved outcomes. Combined diagnostic yield (solved) of SGT and WES was 46% (83/181) with 15% (28/181) classified as possibly solved (Fig. 3A). Combined solved outcomes involved 27 genes (Fig. 3B), with *GJB1*, *PMP22* duplication and *MFN2* most common across 29 (16%), 14 (8%) and four (2%) participants, respectively. Most common WES-identified variants were in genes *MFN2* (four participants, 3%), *GJB1* (three participants, 2.3%), *MPZ* (three participants, 2.3%), *PRX* (three participants, 2.3%*)* and *SH3TC2* (three participants, 2.3%). Twenty-two novel genetic variants (13 pathogenic/likely pathogenic, nine VUS) were identified in 19 genes (Table 1).

**Table 1:** Novel genetic variants identified in the genetic peripheral neuropathy (neuropathy) cohort

Disease category	Gene symbol	Variant	ACMG classification
Neuropathy	*SBF2*	ENST00000256190.13:c.2100+1G>A	Pathogenic
Neuropathy	*NEFL*	ENST00000610854.2:c.796G>T	Pathogenic
Neuropathy	*HSPB1*	ENST00000248553.7:c.504del	LP
Neuropathy	*SH3TC2*	ENST00000504517.5:c.321G>A	LP
Neuropathy	*IGHMBP2*	ENST00000255078.8:c.449+2T>A	LP
Neuropathy	*GAN*	ENST00000648994.2:c.280C>T	LP
Neuropathy	*MPZ*	ENST00000463290.5:c.620_623dup	LP
Neuropathy	*PMP22*	ENST00000312280.9:c.448_449delGGinsTT	LP
Neuropathy	*NEFL*	ENST00000610854.2:c.65_68delCCCGinsT	LP
Neuropathy	*VRK1*	ENST00000216639.8:c.1012A>T	LP
Neuropathy	*MPV17*	ENST00000233545.6:c.176_181del	LP
Neuropathy	*PRX*	ENST00000324001.8:c.1560_1562del	LP
Neuropathy	*PMP2*	ENST00000256103.3:c.19G>C	LP
Neuropathy	*MPZ*	ENST00000463290.5:c.212A>G	VUS
Neuropathy	*MPZ*	ENST00000672602.2:c.772C>G	VUS
Neuropathy	*ATP7A*	ENST00000341514.11:c.2083C>T	VUS
Neuropathy	*SCN11A*	ENST00000302328.9:c.1101G>T	VUS
Neuropathy	*AIFM1*	ENST00000287295.8:c.512T>C	VUS
Neuropathy	*ATL1*	ENST00000358385.12:c.1208G>C	VUS
Neuropathy	*KIF1A*	ENST00000648047.1:c.368A>G	VUS
Neuropathy	*KMT2C*	ENST00000262189.11:c.1013C>T	VUS
Neuropathy	*MPZ*	ENST00000672602.2:c.772C>G	VUS

ACMG = American College of Medical Genetics; LP = likely pathogenic; VUS = variant of uncertain significance.

### Congenital myopathies and muscular dystrophies

One hundred and seven probands underwent WES (72 South African, 22 Turkish, 10 Brazilian, three Indian), with 57 (53%) probands solved (16 genes), 20 (19%) possibly solved (18 genes) and 30 (28%) unsolved (Fig. 3A). Diagnostic yield (‘solved/possibly solved’ outcomes) varied between 0 and 60% across populations. *STAC3* (28), *RYR1* (8) and *COL6A2/3* (5) were the most common genes in solved probands (Fig. 3B). Twenty-one novel genetic variants (12 likely pathogenic, nine VUS) were identified in 14 genes (Table 2).

**Table 2:** Novel genetic variants identified in the congenital myopathy/congenital muscular dystrophy (CM/CMD) cohorts

Disease category	Gene symbol	Variant	ACMG classification
CM/CMD	*NEB*	ENST00000397345.8:c.17502_17510dup	LP
CM/CMD	*LAMA2*	ENST00000421865.3:c.4127T>A	LP
CM/CMD	*RYR1*	ENST00000355481.8:c.6175_6187del	LP
CM/CMD	*MSTO1*	ENST00000245564.2:c.1678C>T	LP
CM/CMD	*PIEZ02*	ENST00000302079.10:c.1345C>T	LP
CM/CMD	*PIEZ02*	ENST00000302079.10:c.5082+2T>C	LP
CM/CMD	*CHCHD10*	ENST00000484558.2:c.262-1_262dup	LP
CM/CMD	*MMF*	ENST00000304593.14:c.744+1G>A	LP
CM/CMD	*NEB*	ENST00000397337.6:c.736dup	LP
CM/CMD	*NEB*	ENST00000397345.8:c.23310del	LP
CM/CMD	*LAMA2*	ENST00000421865.3:c.1170C>A	LP
CM/CMD	*TPM3*	ENST00000271850.11:c.734G>C	LP
CM/CMD	*RYR1*	ENST00000355481.8:c.12323G>A	VUS
CM/CMD	*BICD2*	ENST00000356884.11:c.1993_1998dup	VUS
CM/CMD	*ACTA1*	ENST00000366683.3:c.182A>G	VUS
CM/CMD	*MYH2*	ENST00000245503.10:c.4809G>A	VUS
CM/CMD	*GBE1*	ENST00000429644.7:c.602A>G	VUS
CM/CMD	*RYR1*	ENST00000355481.8:c.9678G>T	VUS
CM/CMD	*MSTO1*	ENST00000245564.8:c.49G>C	VUS
CM/CMD	*FKRP*	ENST00000318584.10:c.1034G>T	VUS
CM/CMD	*PLOD1*	ENST00000196061.5:c.1285G>C	VUS

ACMG = American College of Medical Genetics; LP = likely pathogenic; VUS = variant of uncertain significance.

### Limb girdle muscular dystrophies

Eighty-five probands underwent WES (47 Indian, 13 Turkish, 11 Brazilian, 12 South African, two Zambian) with 37 (44%) patients solved (16 genes), 14 (16%) possibly solved (12 genes) and 34 (40%) unsolved (Fig. 3A). *DYSF* (10), *CAPN3* (9) and *GNE* (3) were the most common genes in solved probands (Fig. 3B). Fifteen novel genetic variants (7 pathogenic/likely pathogenic, 8 VUS) were identified in 10 genes (Table 3).

**Table 3:** Novel genetic variants identified in the limb-girdle muscular dystrophy (LGMD) and duchenne/becker muscular dystrophy (DMD/BMD) cohorts

Disease category	Gene symbol	Variant	ACMG classification
LGMD	*GNE*	ENST00000396594.8:c.1057C>T	Pathogenic
LGMD	*DYSF*	ENST00000258104.7:c.4558del	Pathogenic
LGMD	*DYSF*	ENST00000258104.7:c.3496_3508del	LP
LGMD	*GNE*	ENST00000396594.8:c.2196G>C	LP
LGMD	*GNE*	ENST00000396594.8:c.1000dup	LP
LGMD	*CAV3*	ENST00000343849.3:c.262T>G	LP
LGMD	*DYSF*	ENST00000258104.7:c.856-1G>A	LP
LGMD	*HSPG2* ^a^	ENST00000374676.4:c.14C>T^a^	VUS
LGMD	*SYNE2*	ENST00000344113.8:c.18212G>A	VUS
LGMD	*DYSF*	ENST00000258104.7:c.1781T>C	VUS
LGMD	*DYSF*	ENST00000258104.7:c.5388dup	VUS
LGMD	*KIF5A*	ENST00000286452.5:c.839G>T	VUS
LGMD	*MYH3*	ENST00000583535.6:c.3131A>T	VUS
LGMD	*RYR1*	ENST00000355481.8: c.2321 G>A	VUS
LGMD	*DMD*	ENST00000343523.7:c.1859A>T	VUS
DMD/BMD	*DMD*	ENST00000357033.9:c.2381-1G>C	LP
DMD/BMD	*DMD*	ENST00000357033.9:c.4575_4579del	LP

ACMG = American College of Medical Genetics; LP = likely pathogenic; VUS = variant of uncertain significance.

^a^The *HSPG2* c.14C>T variant was identified in two unrelated participants.

### Duchenne and Becker muscular dystrophies

**Figure 4:**
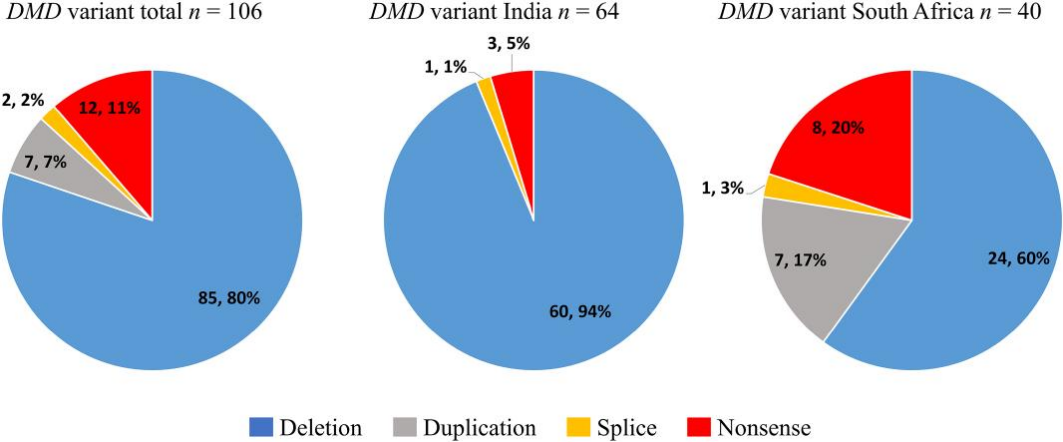
**Variants detected in Duchenne Muscular Dystrophy (DMD) participants with ‘solved’ outcomes**. Blue = deletion; grey = duplication; orange = splice; red = nonsense.

**Figure 5:**
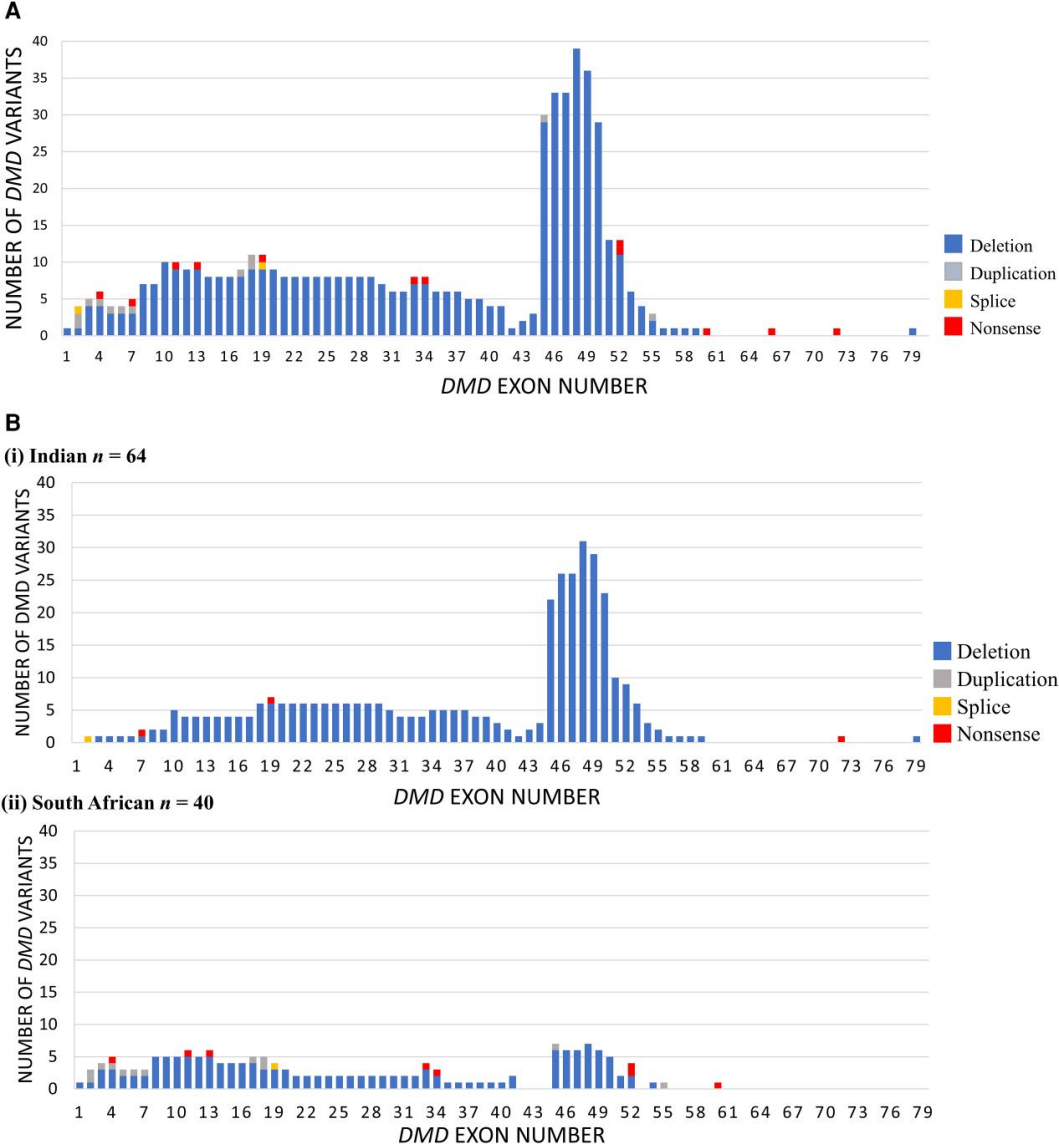
**Distribution of Duchenne Muscular Dystrophy (DMD) variants**. (**A**) Exon distribution of causative *DMD* genetic variants for the whole cohort. (**B**) Exon distribution of causative *DMD* genetic variants in (**i**) Indian and (**ii**) South African cohorts. Blue = deletion; grey = duplication; orange = splice; red = nonsense.

Multiplex ligation probe amplification (MLPA) test for dystrophin (*DMD*) gene deletions and duplications is relatively accessible in partner sites with the exception of Zambia; enabling strong ‘already solved’ ICGNMD participant recruitment for DMD and BMD to combine with new data. DMD MLPA data for 102 probands (94%) were reviewed, plus 17 ICGNMD exomes across a total of 108 probands (64 Indian, 41 South African, three Brazilian). One hundred and six (98%) patients were solved (81 DMD, 23 BMD, two symptomatic female carriers) and two (2%) unsolved after both MLPA and WES. Diagnosis came from *DMD* MLPA in 91 (86%) and WES in 15 (14%) solved outcomes (Fig. 3A). In the solved cohort, 85 (80%) patients had a deletion, 12 (11%) had a nonsense variant (point mutation), seven (7%) had a duplication and two (2%) had a splice variant (Fig. 4). All BMD patients (23/23) had in-frame deletions/duplications. Of the 69 DMD results involving deletions/duplications, 59 (86%) were frameshifts, eight (11%) were in frame and two (3%) involved the initial or terminal *DMD* exon. Of the two symptomatic female carriers one harboured a nonsense variant and the other an out of frame deletion. The prevalence of different types of *DMD* variant varied significantly (Fisher’s exact test, *P <* 0.001) between Indian and South African cohorts. Of 64 solved Indian participants, 60 (94%) carried a deletion, three (5%) a nonsense variant and one (1%) a splice variant. No Indian patients had a duplication. Of the 40 solved South African participants, 24 (60%) carried a deletion, eight (20%) a nonsense variant, seven (17%) a duplication and one a splice variant in *DMD* (Fig. 4). Two solved Brazilian outcomes were due to a *DMD* deletion and a nonsense variant. Analysis of *DMD* breakpoints for the entire cohort revealed two hotspots for deletions and duplications around exons 45–50 and 10–20 (Fig. 5A). There were recurrent deletion/duplication breakpoints in intron 45 (29/92; 32%), 46 (10/92; 11%) and 48 (15/92; 16%), with 59% of deletions/duplications having one breakpoint within introns 45–48. Duplications were spread across the gene with 2/7 occurring at exon 2 (one spanning exons 2–7), 2/7 at exon 18 and 1/7 each at exons 17, 45 and 55. Nonsense variants were spread across the gene between exons 4 and 72 (Fig. 5A). There was a notable difference in proportion of breakpoints affecting different areas of *DMD* between Indian and South African cohorts. Of 54 deletions/duplications involving exon 45–48 breakpoints, 40/54 (74%) were from the Indian cohort, compared with 13/54 (24%) from South Africa. There was also a comparatively higher proportion of deletions/duplications with breakpoints in the proximal 5′ end of the gene (before exon 10) in the South African cohort 9/31 (29%) (six involving exons 1–3), compared to the Indian cohort 3/60 (5%) (Fig. 5B). WES identified two novel pathogenic *DMD* variants (one splice variant and one frameshift deletion). Of the 85 patients with deletions, 44 (52%; 33 Indian and 11 South African) are potentially amenable to antisense oligonucleotide (ASO) exon skipping therapies. Twenty-nine patients (22 Indian, seven South African) carry deletions amenable to licenced exon skipping ASO therapies targeting exons 45 (9), 51 (14) and 53 (5); a single patient carried an exon 52 deletion amenable to either exon 51 or 53 skipping. Of the remaining 15 patients with deletions amenable to exon skipping, six (40%) would be amenable to skipping of exon 44, an ASO currently in clinical trials.

## Discussion

Recent years have seen dramatic advances in gene discovery and genetic diagnosis in NMD. Resulting patient benefits include accurate diagnosis, genetic counselling, improved care pathways including complication screening and prevention, and potential access to clinical trials and disease modifying genetic therapies. However, these benefits and impacts have so far been limited or non-existent in lower-income contexts, despite most NMD patients living in LMICs. Here we explored a principally remote method of connecting academic partners and building international capacity for cohort development and genetic analysis of NMD patients in LMICs. We harnessed key features of genetic analysis, i.e. that samples can be collected remotely and shipped for DNA extraction at relatively low-cost, enabling inclusion of geographically-dispersed patient populations and economies of scale. We took advantage of recent computational tools and high-performance clusters to enable efficient, remote processing of sequence data. We used low-cost cloud-based databases to support rapid and secure sharing of phenotypic data between distant sites. Specifically, we (i) tested feasibility of a distributed transcontinental genomic medicine partnership to build diverse, deeply-phenotyped and genetically characterized cohorts; and (ii) evaluated deploying this partnership and cohort to understand genetic architecture and advance diagnosis. We report 3600 probands and 6001 participants who together represent the first recruits to a new global cohort including previously under-represented populations.

The network of trained fellows working with local PIs and PIs in the UK assembled a deeply phenotyped cohort of children and adults with NMDs. Over 3600 probands’ detailed clinical phenotype and medical histories were recorded in the REDCap database after 43 months (June 2019 to January 2023), and over 2300 affected and unaffected (mainly first degree) family members. The male:female proband ratio (mean 1.86) is higher than reported by other NMD registries.^20^ This may be influenced by referral patterns to some recruitment sites and/or socio-economic circumstances differentially impacting ability to attend appointments.^21^

The data indicates patients with a wide spectrum of neuromuscular diseases joined the ICGNMD study, with a frequency broadly similar to reports from European centres.^1^ The most common NMD clinical diagnoses were genetic peripheral neuropathies, LGMD, CM/CMD and Duchenne or Becker muscular dystrophy, together comprising over half the cohort.

Review of over 820 first proband results (single gene tests and exomes) demonstrated a ‘solved’ rate of approximately 44% and ‘possibly solved’ rate of approximately 15% (i.e. 59% solved/possibly solved). Solved rate increased to over 58% for the four most common disease categories reviewed in depth (LGMD 44%, PN 46%, CM/CMD 53%, DMD/BMD 98%, increasing to LGMD 60%, PN 61% and CM/CMD 72%) (no change to DMD/BMD) when ‘possibly solved’ numbers are included.

The 44% of LGMDs solved by WES included *CAPN3*, *DYSF* and *GNE* variants found at frequencies similar to previously reported,^22^ alongside 16 novel variants. Overall, 1 in 3.5 LGMD WES variants considered disease-causing disease are novel.

Genetic peripheral neuropathies’ solved rate of 46% included single gene tests for common genetic causes. The most common causative genes identified in the genetic peripheral neuropathy cohort (*PMP22* duplication and *GJB1* variants) are similar to those described in previous European and US studies.^23-25^ There were more probands with *GJB1* variants (29 participants, 16%) than the *PMP22* duplication (14 participants, 8%) in our cohort, whereas other cohorts describe a higher rate of *PMP22* duplication participants, usually greater than 50% (e.g. US/UK/European study 61% *PMP22* duplication; 10% *GJB1* variants).^25^ A possible cause of this is that 114/181 probands in the current study were from Brazil, where *PMP22* duplication was excluded in many enrolled participants.

Congenital myopathies and muscular dystrophies yielded a comparable ‘solved’ rate of 53%, spanning 16 genes, including known variants in *STAC3*, *RYR1* and *COL6A2/3* in addition to 21 novel variants across 14 genes. There is a notable lack of solved patients with *COL6A1*- and *TTN-*related CM/CMD (two of the most common forms)^26-29^ and further WES is underway.

The dystrophin gene diagnostic rate was high (98%), with MLPA and WES contributing 86% and 14% of diagnoses, respectively. Deletions and duplications were most common (87% solved participants), in keeping with previous studies.^30,31^ Comparisons between Indian and South African cohorts identified differences in both types of genetic variants (deletions, duplications, nonsense, splice variants) and their distribution (including intronic breakpoints) within the DMD gene. The South African cohort demonstrated a higher number of duplications and nonsense mutations and a higher proportion of intronic breakpoints in the proximal 5′ end of the gene. This could be due to differences in cohort size and variation in patient recruitment (e.g. locally solved via MLPA versus unsolved patients), however a comparatively low proportion of large deletions in South African populations is reported.^32^ It will be important to interrogate reasons for this observation as there may be implications for applicability of exon-skipping therapies.

Overall, our data indicate that, depending on the NMD diagnostic category, 44–98% of patients in LMIC settings may receive an accurate genetic diagnosis with single gene tests and/or WES, creating potential for benefit. We increased the reported genetic diversity associated with NMD, since 1 in 3.5 mutations were novel variants. These data also indicate an additional 15% of probands with a strong VUS candidate and ‘possibly solved’ classification require further evaluation/functional studies to confirm pathogenicity with corollary benefits for discovery research and pharmaceutical insights. The 28% of probands for whom no convincing variants were identified represent an important new diverse discovery cohort for further analysis including whole genome and long read approaches.

The ICGNMD team and results depended on international collaborations established at a smaller scale over the preceding decade, building trust and mutual understanding of local populations, facilities and perspectives. Such collaborations benefited from local computing and data- and material storage infrastructure, small-scale pump-priming, initiatives promoting clinical and genetic data interoperability, and gene-matching platforms. The partnership has potential for additional bidirectional benefit, including enabling deeper understanding of VUS, which is relevant to NMD patients in all countries, and the partnership is a foundation on which to build expanded testing capacity, tailored care guidelines and clinical trial readiness.

In conclusion, we demonstrated that it is feasible to set up a virtual transcontinental partnership using a cloud-based platform and harness big data to describe phenotypes and causative variants present in a diverse cohort recruited from many LMIC settings. Over half of tested participants obtained a research-based genetic diagnosis, opening up potential benefts to patients of an accurate DNA diagnosis and demonstrating feasibility of including more diverse populations in clinical trials. We recognize there are limitations to this study. We did not seek to collect epidemiological data, and our study does not allow conclusions about incidence or prevalence of NMD or genes. On the other hand, this study is in a ‘real-world’ setting, reflecting current practice in each LMIC centre, and shows that despite limitations, a cross-NMD solved rate of 44–59% can be achieved in this previously genetically untested population. This work indicates that geographical inequalities of access to an accurate DNA-based diagnosis can potentially be addressed through such virtual partnerships. These have genuine bidirectional value to all partners and the wider research community. Increasing the knowledge of genetic diversity can improve reliable variant interpretation and therefore the accuracy of genetic diagnosis for patients in both low- and high-income settings.

## Supplementary Material

awad254_Supplementary_Data

## Data Availability

At the end of the study, participants de-identified exome and genome data will be archived in the European Molecular Biology Laboratory European Bioinformatics Institute’s European Genome-Phenome Archive (EMBL EBI EGA), with community access to this and selected de-identified REDCap data managed via an ICGNMD Data Access Committee.
