# Supplementary material for: Neuromuscular disease genetics in under-represented populations: increasing data diversity
Source: Brain. 2023 Jul 30;146(12):5098–109. doi: 10.1093/brain/awad254 (PMC10690022; doi:10.1093/brain/awad254)
Supplement: awad254_Supplementary_Data [file awad254_supplementary_data.zip › brain-2023-00969-File010.pdf]

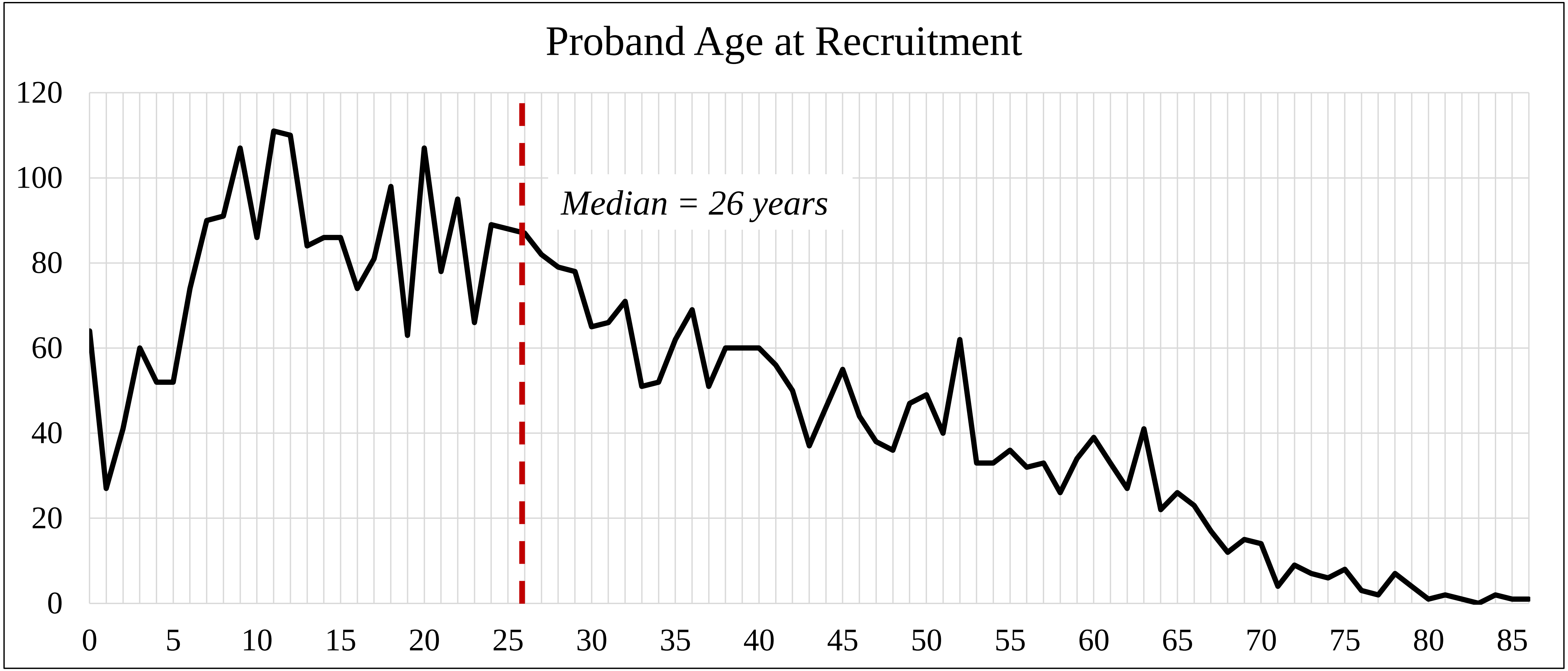

Supplementary Figure 1 Number of participants by age. Age ranges from new-borns to 86 years old and with median age at recruitment of 26 years old.

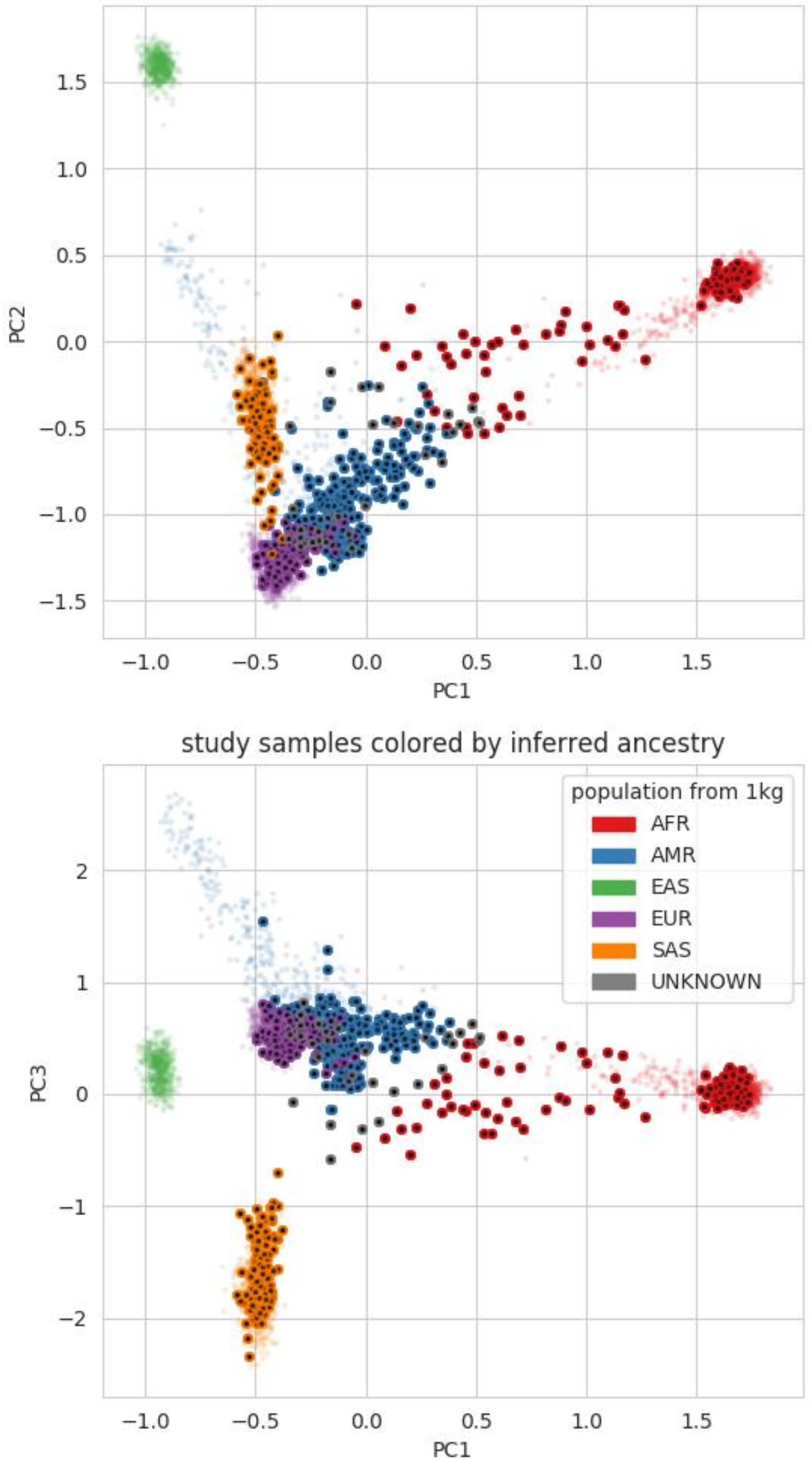

Supplementary Figure 2 Principal component analysis (PCA) plot of predicted ethnicity. PCA clusters generated for 611 exome samples using 1000 Genomes (1kg) population data as implemented in Peddy (*Supplementary Methods Ref 15*). The plot shows the majority of ICGNMD samples are of non-European ancestry and admixed. Dots with black centre represent ICGNMD samples. Faint background colour dots show 1kg samples. AFR=African; AMR=Admixed Americans; EAS=East Asians; EUR=European; SAS=South Asians.

Supplementary Table 1 ICGNMD partner sites (see also author affiliations and https://www.ucl.ac.uk/genomic-medicine-neuromuscular-diseases/global-contributor-list). Where ethics require the majority of DNA samples to remain in-country, local testing sites (India and Turkey) generate raw data and share with UCL for integration to the common ICGNMD pipeline. While UK partner sites recruit participants to the Study, in this paper we only include data from participants living in low-to-middle income partner sites.

**Country**	**ICGNMD Partner Sites**
Brazil	Da Faculdade de Medicina de Ribeirão Preto da Universidade de São Paulo (FAEPA), São Paolo
India	All India Institute of Medical Science, New Delhi (AIIMS Delhi), Delhi<br>National Institute of Mental Health and Neurosciences (NIMHANS), Bangalore<br>Nizam's Institute of Medical Sciences (NIMS), Hyderabad<br>*Genetic Analysis*:<br>Centre for DNA Fingerprinting and Diagnostics (CDFD), Hyderabad (single gene tests)<br>CSIR Centre for Cellular and Molecular Biology (CSIR-CCMB), Hyderabad (whole exome)
Netherlands	*Genetic Analysis (FSHD):* Leiden University Medical Centre (LUMC), Leiden
South Africa	Stellenbosch University, with recruitment at Tygerberg Academic Hospital, Belville<br>University of Cape Town, with recruitment at Groote Schuur Hospital and Red Cross War Memorial Children's Hospital, Cape Town<br>University of Pretoria, with recruitment at Steve Biko Academic Hospital, Pretoria<br>North-West University, Potchefstroom
Turkey	Izmir Biomedicine and Genome Centre and Institute (IBG), Dokuz Eylül University, Izmir<br>*Genetic Analysis*:<br>Yıldırım Beyazıt University and Ankara City Hospital, Genetic Diseases Diagnostic Centre (whole exome), Ankara
United Kingdom	UCL Queen Square Institute of Neurology, University College London, London with recruitment at University College London Hospitals NHS Foundation Trust<br>UCL Great Ormond Street Institute of Child Health, University College London, London with recruitment at Great Ormond Street.<br>University of Cambridge, Cambridge, with recruitment at Cambridge University Hospitals NHS Foundation Trust<br>Newcastle University, Newcastle upon Tyne, with recruitment at Newcastle upon Tyne Hospitals NHS Foundation Trust
Zambia	University of Zambia, with recruitment at University Teaching Hospital Lusaka<br>Additional PI affiliation and ethics approval from University of Rochester School of Medicine, Rochester (USA)

Supplementary Table 2 Cohort by probands and all participants (probands, affected and unaffected relatives) across ICGNMD partner countries. Only participants with data recorded in the ICGNMD database at mid-January 2023 counted. See Table 1 for details of recruitment locations in each country.

Country	Probands	All participants	% total probands	% total cohort
India	2316	3578	64	60
Brazil	645	979	18	16
South Africa	433	737	12	12
Turkey	183	578	5	10
Zambia	54	129	1	2
Total	3631	6001	100	100

Supplementary Table 3 Number of probands assigned to each of 15 clinical diagnostic categories in the ICGNMD database and % of each category of total (not shown for participants with "Complex Phenotypes" awaiting a diagnostic category).

Clinical Diagnostic Category	Number of Probands	% Clinical Diagnoses
Adult onset myopathy	27	0.7
Skeletal muscle channelopathy	55	1.5
Myotonic dystrophy Type I	89	2.5
Metabolic myopathy	105	2.9
Genetic motor neurone disease	111	3.1
Distal myopathy	115	3.2
Congenital myasthenic syndrome	156	4.3
Spinal muscular atrophy (SMA)	181	5.0
Mitochondrial disease	212	5.8
Facioscapulohumeral muscular dystrophy (FSHD)	234	6.4
Duchenne or Becker muscular dystrophy (DBMD)	313	8.6
Congenital muscular dystrophy or myopathy (CM/CMD)	343	9.4
Complex phenotype (mixed CNS and NMD)	471	n/a
Genetic peripheral neuropathies (PN)	561	15.5
Limb girdle muscular dystrophy	658	18.1

Supplemental Table 4 Summary of single gene analyses supported by the ICGNMD project up to January 2023.

See Supplemental Methods for further details.

Test	Total Tested	Solved	Unsolved	% Solved
Myotonic Dystrophy Type I (*DMPK* [a]Triplet Repeat Primed PCR[b])	20	19	1	95.0
Duchenne Muscular Dystrophy (Dystrophin (*DMD*) MLPA)	12	5	7	41.7
Friedreich's Ataxia (Frataxin (*FXN*) Triplet Repeat Primed PCR)	3	2	1	66.7
Spinal and bulbar muscular atrophy (SBMA; Kennedy's Disease) (androgen receptor (*AR*) repeat expansion (fluorescently labelled PCR)	6	3	3	50.0
Oculopharyngeal muscular dystrophy (OPMD) (PCR)	3	1	2	33.3
Spinal Muscular Atrophy (SMA) (*SMN* MLPA)	8	6	2	75.0
India CMT1[c] (MLPA[d]: *PMP22/MPZ/GJB1* dup/del)	9	1 *PMP22* deletion	8	11.1
Brazil CMT1 (*PMP22* dosage analysis)	55	14 *PMP22* duplications	41	25.5
Brazil CMT1 (*PMP22/GJB1/MPZ* PCRs)	60	26	34	43.3
Facioscapulohumeral Muscular Dystrophy (FSHD) (Southern Blot & Methylation analysis)	98	60	38	61.2
**Totals**	**274**	**137**	**137**	**50.0**

Abbreviations: [a]*DMPK*: myotonic dystrophy protein kinase, [b]PCR: polymerase chain reaction, [c]CMT1: Charcot-Marie-Tooth disease type 1; [d]MLPA: multiplex ligation-dependent probe amplification.
