# Supplementary material for: Neuromuscular disease genetics in under-represented populations: increasing data diversity
Source: Brain. 2023 Jul 30;146(12):5098–109. doi: 10.1093/brain/awad254 (PMC10690022; doi:10.1093/brain/awad254)
Supplement: awad254_Supplementary_Data [file awad254_supplementary_data.zip › brain-2023-00969-File011.pdf]

Supplementary Data: Methods

Ethics

International studies with a single sponsor that require identical ethics across all participating countries and sites can sometimes have barriers to participation (for example, mandating wider data sharing without local approval, or provisions for data sharing with for-profit entities). To address this, the ICGNMD cohort was contributed to by multiple, aligned studies. All participating sites were requested to implement locally sponsored Study Ethics aligned as closely as possible to UK Study documentation (which drew on GA4GH guidance), while also respecting local governance and rules. As a minimum, sites must permit the essential activities of the study, including international data sharing and publication of data. Local ethics also determine wider data sharing permissions for each participating site (see Data Sharing below).

Supplementary Methods Table 1 Study names and ethics approval details of each participating site.

Participant Recruitment Site	Study Name	Ethics References
Ribeirão Preto Medical School University of São Paulo Foundation for the Support of Teaching, Research and Service of the University Hospital (FAEPA)	International Centre for Genomic Medicine in Neuromuscular Diseases (ICGNMD)	Clinical Research Unity (UPC) and Local Ethics Committee (CEP) Approval letter dated 05/05/2020<br><br>National Research Ethics Commission (Comissão Nacional de Ética em Pesquisa (CONEP) Approval letter dated: 24/Nov/2020
All India Institute of Medical Science, New Delhi (AIIMS Delhi), India	International Centre for Genomic Medicine in Neuromuscular Diseases	IEC-237/05.04.2019, AA-2/3.05.2019<br><br>HMSC Ref 2020-9274
National Institute of Mental Health and Neurosciences (NIMHANS), India	1. Genomic Research in Mitochondrial disorders & inherited peripheral neuropathy – an international collaborative study (Neuropathology Group)<br>2. International Centre for Genomic Research in Neuromuscular Diseases (Neurology Group)	1. NIMHANS IEC (Basic and Neurosciences Division) Letter dated 11.11.2019<br>2. NIMHANS IEC (Basic and Neurosciences Division) Letter dated 25.06.2020<br><br>HMSC Ref 2020-9274
Nizam's Institute of Medical Sciences (NIMS), India	International Centre for Genomic Medicine in Neuromuscular Diseases (ICGNMD)	NIMS Institutional Ethics Committee Reference EC/NIMS/2431/2020<br><br>HMSC Ref 2020-9274
University of Cape Town, South Africa	International Centre for Genomic Medicine in Neuromuscular Diseases, Sub-Study R030/2018 (ICGNMD)	University of Cape Town Faculty of Health Sciences Human Research Ethics Committee HREC Ref: 601/2019
University of Cape Town, South Africa	Inherited Neuromuscular Disease research: moving towards a definitive molecular diagnosis for each patient. INMD_G and INMD_R databases	University of Cape Town Faculty of Health Sciences Human Research Ethics Committee HREC Ref: R030/2018 and amended 05/03/2019
Stellenbosch University, South Africa	Stellenbosch University Inherited Neuromuscular Disorders Registry and DNA Repository	Health Research Ethics Committee at Stellenbosch University Ref B19/01/002
University of Pretoria, South Africa	International Centre for Genomic Medicine in Neuromuscular Disease: A Systems Biology Approach in Neuromuscular or Neurogenetic Diseases	University of Pretoria Faculty of Health Sciences Research Ethics Committee Ref 296/2019
Ankara City Hospital, Ankara & Izmir Biomedicine and Genome Centre (IBG), Izmir, Turkey	Nöromusküler Hastaliklarda Genomik Tip Için Uluslararasi Merkez Oluşturulmasi	Dokuz Eylül Üniversitesi Klinik Araştırmalar Etik Kurulu; 477-SBKAEK<br><br>Ankara Şehir Hastanesi<br>Genetik Hastaliklari Tani Merkezi Approval letter dated 04/11/2019 and<br>Ankara Yıldırım Beyazıt Üniversitesi Tıp Fakültesi Approval letter dated 10/10/2019
University College London (UCL), University of Cambridge & University of Newcastle, United Kingdom	International Centre for Genomic Medicine in Neuromuscular Diseases (ICGNMD)	UK Study Sponsor: University College London: Joint Research Office Reference 125328<br><br>NHS Health Research Authority London – Camberwell St Giles Research Ethics Committee Reference 19/LO/1796
University Teaching Hospital, Lusaka, Zambia	Genomic Research in Neuromuscular Disorders in<br>Zambia: A Prospective Cohort Study of Genetic<br>Causes and Clinical Outcomes in Patients with<br>Neuromuscular Disorders (GRINZ)	RSRB, University of Rochester, USA Ref STUDY00003813 and University of Zambia Biomedical Research Ethics Committee Ref 091-2019.

Data Storage and Data Sharing

Data sharing between ICGNMD partners

The study's genetic data is classified as personal data and special category genetic data under UK General Data Protection Regulations (UK GDPR). These regulations apply to all study data because genetic data is stored on UCL-owned physical hardware hosted in the UK, or in a UK/EU hosted Cloud. REDCap ICGNMD database phenotypic data and genetic reports are also stored in a Cloud-based server hosted in the UK/EU and also fall under UK GDPR. UK GPDR consequently apply as a "minimum universal standard" to all study data sent outside of partner sites, in addition to any local country requirements that apply to data held solely at the local site or shared overseas. As the data for the study is generated by both local sites and UCL, each local site and UCL act as "joint data controllers" of the final dataset and discuss in advance any plans for data sharing.

The nature of the study requires all partner sites to share data with each other. To this end, the ICGNMD established a multi-party data sharing agreement (ICGNMD DSA) that sets out data sharing and storage requirements across parties and confirms GDPR for all data. Prior to sharing between specific sites, recipient sites complete a Risk Assessment Form listing local data processors and describing local data storage locations and levels of security then collaborating partners complete a Schedule to the DSA which confirms the exchange can take place.

Study participants are able to request access to their data according to local ethics and governance rules, however where genetic data is shared, this must be communicated as "research only" data and may not be used to inform clinical interventions without validation in a locally-approved clinical diagnostic context.

It is widely acknowledged that low-to-middle income (LMIC) partners have limited resources for data review, validation, publication preparation, IP evaluation and integration into local capacity building initiatives. The 12-month embargo prior to external data sharing mandated by many internationally-funded genetics projects is often insufficient time for LMIC partners to deliver local benefits in full, particularly for Mendelian diseases where a sizeable cohort may take several years to recruit. The ICGNMD applies a minimum 24-month embargo (post-genetic report generation) on external data sharing, with additional flexibility where there is a demonstrable need to recruit multiple individuals locally to derive meaningful data. This embargo does not preclude early external collaborations but is the minimum time before which the "expectation to freely share data" applies.

External Data Sharing

At the end of the study, participants de-identified exome and genome data will be archived in the EMBL European Bioinformatics Institute's European Genome-Phenome Archive (EMBL EBI EGA)[1], with community access to this and selected de-identified REDCap data managed via an ICGNMD Data Access Committee. To adhere to local ethics specifications, each partner's archived data will be tagged with use limitations using GA4GH Data Use Ontology (DUO) codes. Where local ethics permit, a subset of participants' pseudonymised data will be shared via the RD-Connect Genome-Phenome Analysis Platform (GPAP)[2]. GPAP is designed for use by clinicians and researchers without specialist informatics training and so may enhance its uptake across sites with limited access to bioinformatics support. While a centralised ICGNMD data resource may not preclude the need for some external collaborators to enter into formal collaborations with more than one partner to use country-specific data, UCL is authorised by ICGNMD partners to act as signatory in external data sharing agreements to streamline external engagement and allow a single point of liaison for multiple sites' data.

Supplementary Methods Table 2 Data Use Ontology codes applying to ICGNMD partner site data under existing ethics.

ICGNMD Site	Study ID Prefix	All Sites [DUO:00000]	All Sites [DUO:00000]	All Sites [DUO:00000]	All Sites [DUO:00000]	All Sites [DUO:00000]	All Sites [DUO:00000]	All Sites [DUO:00000]	Site Specific: [DUO:00000]
AIIMS	IC_AIM	19	21	25	26	27	28	44	07 (NMDs), 15
NIMS	IC_NIM	19	21	25	26	27	28	44	06 (health/med/biomed)
NIMHANS Neurology	IC_NAN	19	21	25	26	27	28	44	06 (health/med/biomed), 20, 46
NIMHANS Neuropathology	IC_NAS	19	21	25	26	27	28	44	06 (health/med/biomed), 20
FAEPA	IC_BAP	19	21	25	26	27	28	44	07 (NMDs), 15
UTH Lusaka	IC_UTH	19	21	25	26	27	28	44	07 (NMDs), 15, 18
Ankara	IC_TAN	19	21	25	26	27	28	44	06 (health/med/biomed), 20
Izmir	IC_TIM	19	21	25	26	27	28	44	06 (health/med/biomed), 20
Stellenbosch	IC_SAB	19	21	25	26	27	28	44	07 (NMDs)
Pretoria	IC_SPR	19	21	25	26	27	28	44	07 (NMDs)
Cape Town RCCH	IC_SRC	19	21	25	26	27	28	44	07 (NMDs)
Cape Town GSH	IC_SGS	19	21	25	26	27	28	44	07 (NMDs)

Abbreviations RCCH; Red Cross War Memorial Children's Hospital, Cape Town, GSH; Groote Schuur Hospital. DUO codes: 19; requestor agrees to make results of studies using the data available to the larger scientific community, 21; requestor must provide documentation of local IRB/ERB approval, 25; use is approved for a specific number of months, 26; use is limited to use by approved users, 27; use is limited to use within an approved project, 28; use is limited to use within an approved institution, 44; use for purposes of population, origin, or ancestry research is prohibited (without additional ethics approvals), 06; use is allowed for health/medical/biomedical purposes; does not include the study of population origins or ancestry, 07; use is allowed provided it is related to the specified disease (neuromuscular disease), 15; use includes methods development research(e.g., development of software or algorithms) only within the bounds of other use limitations, 18; use is limited to not-for-profit organizations, 20; the requestor must agree to collaboration with the primary study investigator(s), applied here by default if data use always involves new ethics approval by the local site, 46; use of the data is limited to not-for-profit use.

REDCap data entry

The REDCap database[3,4] is accessed via 2-factor authentication and data entry may be online or offline to accommodate intermittent internet access. Recruitment sites have ring-fenced Data Access Groups accessible only to authorised personnel at that site and the central administration team in the UK. Each participant is assigned a unique ICGNMD Study ID (IC_SiteID_Number) for pseudonymisation. Standardised data entry is encouraged via use of Human Phenotype Ontology (HPO) [5] terms (for muscle biopsies, and assay, biochemistry and imaging results, as well as clinical phenotype), Orphanet terms (http://www.orpha.net) and standard neuromuscular disease scales, namely the 6 minute walk test [6], MRC Scale for Muscle Power (Aids to the Examination of the Peripheral Nervous System (Memorandum No.45)) used with the permission of the Medical Research Council, Revised Hammersmith Scale for Spinal Muscular Atrophy [7], Charcot-Marie-Tooth Neuropathy Score (CMTNS) [8], Charcot-Marie-Tooth disease paediatric scale (CMTPedS) [9] and North Star Ambulatory Assessment (NSAA) [10] and Apgar Score [11]. Training in use of HPO terms and scales was provided at the study induction, and consistency of REDCap use was evaluated using "mock cases". To improve use of semantic similarity scoring tools such as Exomiser[12], recruiters are encouraged to select a minimum of 5 positive, relevant HPO terms to help ongoing efforts to optimise automated genetic variant identification. Pertinent negative findings e.g. the absence of intellectual disability, cardiomyopathy or abnormal MRI brain were also recorded with HPO terms. The database contains 8 main sections: (1) "*Core Data*" (with space to confirm consent and record participants' family status (proband or specified relative), sex, age at recruitment, age of symptoms onset, self-reported ethnicity (terms defined by each recruiting country), HPO terms, clinical diagnosis, progression and family history of disease); (2) "*Other Medical History*" (from birth, and including past surgical history, in addition to space to record handedness, smoking and alcohol consumption); (3) "*Clinical Assessment*" (enabling detailed NMD symptoms recording, including cardiac and respiratory involvement); (4) "*Investigations*" (to record clinical tests where available, including EMG, nerve conduction studies, echocardiogram, MRI, ultrasounds, biopsies and blood tests, including for viruses and other infectious diseases; (5) "*Non-ICGNMD Genetic Testing*" where genetic tests conducted outside of the current study can be recorded; (6) "*Sample Tracking*", (7) "*Cohort Management*" to record if a participant has died after recruitment, and; (8) "*Monitoring & Analysis*" where study discussions, test decisions and outcomes are recorded. The latter contains checkboxes for the team to select the most commonly-used PanelApp panels to filter whole exome data (*see*

main text), with free text options to request less-common ones. A PDF of the instrument is provided as Supplemental data. Recruiting partners are asked to complete, as a minimum, details of Participant consent, Study ID, their proband or family member status, sex, age at recruitment, initial clinical diagnosis and HPO terms.

Whole exome sequencing and variant prioritisation

Libraries were generated in four centres (Macrogen Europe (Amsterdam, The Netherlands), MedGenome (Bangalore, India), CCMB (Hyderabad, India), Genetic Diseases Diagnostics Centre, Ankara City Hospital (Ankara, Turkey)) using SureSelect All Exon V6 (Agilent), Twist Comprehensive Exome Panel (Twist Bioscience), TruSeq DNA Exome (Illumina) or QIAseq Human Exome (QIAGEN). Samples were sequenced on Illumina Novaseq 6000 platform to a minimum of 25x coverage.

Fastq files were aligned to the human reference genome (GRCh38) using BWA-MEM v.0.7.17[13] and variant calling followed GATK v.4.1.9.0 best practices pipeline[14]. Alignment and variant calling quality control was calculated using GATK. Relatedness and sex mismatches were detected using Peddy v.0.4.8[15]. After quality control, variant files were annotated using Ensembl Variant Effect Predictor v.103[16] to facilitate variant interpretation and prioritization. Variant allele frequencies were collected from several cohorts including gnomAD[17], an in-house database, SAHGP[18], and H3Africa[19].

Only rare (MAF < 0.01) variants in genes present in virtual gene panels applied to each patient were retained for automated reporting. Gene panels selection from Genomics England PanelApp[20] was carried out during genetic multidisciplinary meetings where each case was discussed. An automated tiering system for variant prioritisation was implemented based on Genomics England pipelines[21]. Cases unsolved after review of automated reports undergone manual analysis of all variants.

ICGNMD Single Gene Tests

CMT:

CMT testing in Brazil: In Brazil, sequential testing for suspected demyelinating neuropathy is as follows, (halting if positive result returned): (i) PMP22 duplication/deletion testing via low-cost microsatellite and restriction fragment length polymorphism analysis [22] followed by

MLPA if negative, (ii) PMP22 sequencing, (iii) MPZ sequencing and (iv) GJB1 sequencing. Where family history suggests a specific gene, testing may be focused there first.

PMP22 duplications and deletions with microsatellite markers and PCR-RFLP: The PCR conditions are available upon request. Amplified DNA fragments were digested with the EcoR I restriction enzyme according to the manufacturer's instructions (Invitrogen, Thermo Fisher, MA, USA), electrophoresed on a 1.0% agarose gel.

PMP22 duplications and deletions with MLPA: Multiplex ligation-dependent probe amplification (MLPA) was performed using The SALSA MLPA Probemix P033-B4 CMT1 kit according to the manufacturer's instructions (MRC Holland, Amsterdam, the Netherlands). MLPA products (detecting deletions or duplications in the PMP22 and KIF1b genes) were resolved by capillary electrophoresis and analysed with the Coffalyser.net software (MRC Holland; www.mlpa.com).

Sanger sequencing: All exons and flanking intronic regions of PMP22, MPZ and GJB1 genes were amplified by PCR on extracted DNA. Primer sequences and polymerase chain reaction conditions are available upon request. PCR products were purified and sequenced bidirectionally using ABI PRISM 3500 XL equipment (Applied Biosystems®). Sequence analysis was performed with the Sequencing Analysis Software 6 (Applied Biosystems®) and SeqMan (DNASTAR®) programs. Variants were named according to Human Genome Variation Society (HGVS) and confirmed using the Mutalyzer tool. Nucleotides were numbered in accordance with the following following nerve-specific transcript: PMP22 (RefSeq Transcript: NM_000304.4); *MPZ* (RefSeq Transcript: NM_000530.8) and *GJB1* (RefSeq Transcript: NM_000166.5).

CMT testing in India (CDFD):

PMP22 duplications and deletions with MLPA: Multiplex ligation-dependent probe amplification (MLPA) was performed using The SALSA MLPA Probemix P405-B1 CMT1 kit according to the manufacturer's instructions (MRC Holland, Amsterdam, the Netherlands). MLPA products (detecting deletions or duplications in PMP22, MPZ and GJB1) were resolved by capillary electrophoresis and analysed with the Coffalyser.net software (MRC Holland; www.mlpa.com).

Duchenne Muscular Dystrophy (all sites):

DNA was extracted from peripheral blood and tested using the P034-DMD-1 and P035-DMD-2 SALSA multiplex ligation-dependent probe (MLPA) probemix (MRC-Holland, Amsterdam, The Netherlands). MLPA products were resolved by capillary electrophoresis and analysed with the Coffalyser.net software (MRC Holland; www.mlpa.com).

Spinal Muscular Atrophy:

SMA testing in South Africa:

DNA was extracted from blood or other bodily tissues, followed by PCR amplification and restriction enzyme digestion using HinfI to detect a homozygous deletion of SMN1 exon 7. Mutations were classified according to GenBank Accession Number NM_000344.4 (CCDS Accession Number 34181.1). Nucleotide 164 was counted as the first nucleotide of transcription. The test can detect homozygous deletion of SMN1 exon 7 which causes recessive SMA in approximately 95-98% of SMA patients worldwide and approximately 55% of childhood-onset black South African SMA patients. This test does not detect heterozygous SMN gene deletions or any other mutation in the SMN gene.

SMA testing in India (CDFD):

DNA was extracted from peripheral blood and tested using the P021 and P60 SMA Carrier SALSA multiplex ligation-dependent probe (MLPA) kits (MRC-Holland, Amsterdam,The Netherlands). All reactions were performed according to the manufacturer’s protocols. MLPA products were resolved by capillary electrophoresis ABI 3500 genetic analyzer (ThermoFisher Scientific) and analysed with the Coffalyser.NET software (www.mlpa.com). Probemix P021 detects in patients copy number changes in copy number changes in SMN1 exon 7 and 8, SMN2 exon 7 and 8, SMN1+2 exon 1-6 and SMN1+2 exon 7+8. Probemix P60 detects deletions or duplications in exons 7 and 8 of the SMN1 and SMN2 genes in genomic DNA of carriers.

Myotonic Dystrophy, Type I (DM1):

DM1 at UCL, UK:

Short PCR: Genomic DNA was extracted from whole blood. 50 ng of DNA was amplified in a 25µl total volume of Fast-start Master Mix (7.5µl, Merck), PCR grade water (5µl) and 10µM of each forward (CTTCCCAGGCCTGCAGTTTGCCCATC) and reverse (AACGGGGCTCGAAGGGTCCTTGTAGC) primers. The reactions were subjected to 1 cycle of 95°C for 4 min, and 35 cycles of 95°C for 30 s, 70°C for 30 s and 72°C for 60 s, followed by a final extension time at 72°C for 5 min. The PCR products were electrophoresed on 2% agarose gel at 90 V for 35 min to check for the presence of a PCR product.

Triplet Repeat Primed PCR (TP-PCR): 50 ng of DNA was amplified in a 25µl total volume of FailSafe PCR 2X PreMix H (13µl, Lucigen), PCR grade water (8 µl) and 10µM FAM labelled forward (6FAM- CTTCCCAGGCCTGCAGTTTGCCCATC), anchor (GGCGGTGGCGGCTGTTG) and reverse (GGCGGTGGCGGCTGTTGCTGCTGCTGCTGC) primers (1µl each). The reactions were subjected to 1 cycle of 95°C for 3 min, and 30 cycles of 95°C for 50 s, 61°C for 60 s and 72°C for 1 min, followed by a final extension time at 72°C for 3 min. Products were separated on an ABI PRISM 3730 × l genetic analyzer (Life Tech, Grand Island, NY 14072, USA). 1µl of PCR product was mixed with 0.1µl of LIZ-500 size standard (ThermoFisher) and 9.2µl of HiDi Formamide (ThermoFisher). The mixture was denatured at 86°C for 3 min then loaded onto the 3730 × l genetic analyzer. The data was analysed with Geneious Prime (version 2023.0.2) where an expansion was visualised as a decremental saw-tooth pattern, and the size was measured against LIZ-500 ladder.

DM1 in India (CDFD):

Short PCR: Genomic DNA was extracted from whole blood. 50 -100 ng of DNA was amplified in a 10µl total volume of Taq PCR Master Mix (5ul) (Qiagen), PCR grade water (2µl) and 10pmoles/µl of each forward FAM labelled (5’-6 FAM - CTTCCCAGGCCTGCAGTTTGCCCATC) and reverse (GAACGGGGCTCGAAGGGTCCTTGTAGC) primers (0.5µl each) . [23] The reactions were subjected to 1 cycle of 95°C for 5 min, and 35 cycles of 95°C for 10 s, 62°C for 30 s and 72°C for 30 s, followed by a final extension time at 72°C for 10 min. The PCR products were electrophoresed on 2% agarose gel at 90 V for 35 min to check for the presence of a PCR product.

Triplet Repeat Primed PCR (TP-PCR): 100 ng of DNA was amplified in a 12.5µl total volume of Taq PCR 2X PreMix H (5µl, Qiagen), PCR grade water (2.0µl) and 10pmoles/µl FAM labelled forward (6FAM-AGAAAGAAATGGTCTGTGATCCC) (1 µl), anchor (TACGCATCCCAGTTTGAGACG) (1µl) and reverse TACGCATCCGAGTTTGAGACGTGCTGCTGCTGCTGCT (0.3µl) primers. The reactions were subjected to 1 cycle of 96°C for 10 min, and 30 cycles of 94°C for 60 s, 60°C for 60 s and 72°C for 60 s, followed by a final extension time at 72°C for 10 min. Products were separated on an ABI 3500 genetic analyzer (ThermoFisher Scientific). 4µl of PCR product was mixed with 0.3µl of LIZ-500 size standard (ThermoFisher) and 10µl of HiDi Formamide (ThermoFisher). The mixture was denatured at 94°C for 4 min then loaded onto the 3500 genetic analyzer. The data was analysed with Genemarker software (v2.2.0) where an expansion was visualised as a decremental saw-tooth pattern, and the size was measured against LIZ-500 ladder.

Allele sizes are calculated using internal size standard, from which repeat size is obtained by subtracting size of non-repeat region from size of the PCR product divided by 3 (triplet repeat). Individuals beyond the threshold of 150 bp are considered as affected with myotonic dystrophy type 1.

OPMD PCR (UCL, UK)

Genomic DNA was extracted from whole blood. 50ng of DNA was amplified in a 25µl total volume of PCR Reaction Buffer, with 20 mM MgCl2 10x concentrated (2.5µl, Roche), dNTPs (0.5µl, Roche), Fast start Taq DNA polymerase (0.5µl, Roche), PCR grade water (13.75µl) water and 10µM of each forward (TGGCGCAGTGCCCGCCTTAGA) and reverse (CCAGTTCCTCAGACTCCAGG) primers. The reactions were subjected to 1 cycle of 96°C for 2 min, and 38 cycles of 96°C for 30 s, 61°C for 30s and 72°C for 30 s, followed by a final extension time at 72°C for 7 min. Enzymatic clean-up was performed with 3µl of ExoSap-IT (ThermoFisher) and 7µl of the PCR product. Sanger sequencing was performed at Source Bioscience (Cambridge) and the sequences were aligned and analysed with Geneious Prime (version 2023.0.2).

Friedreich's Ataxia (CDFD India)

Short PCR: Genomic DNA was extracted from whole blood. 50 -100 ng of DNA was amplified in a 10µl total volume of Taq PCR Master Mix (5ul) (Qiagen), PCR grade water (2µl) and 10pmoles/µl of each forward FAM-labelled (5'-6 FAM-GGCTTAAACTTCCCACACGTGTT) and reverse (AGGACCATCATGGCCACACTT) primers (0.5µl each). [24]The reactions were subjected to 1 cycle of 95°C for 5 min, and 10 cycles each of Denaturation (96°C for 1min) and Annealing and Extension at 68°C for (i) 3 min, (ii) 7 min and (iii) 10 min.

Triplet Repeat Primed PCR (TP-PCR): 100 ng of DNA was amplified in a 12.5µl total volume of Taq PCR 2X PreMix H (5µl, Qiagen), PCR grade water (2.0µl) and 10pmoles/µl forward primer (GCTGGGATTACAGGCGCGCGA) (1 µl), anchor (TACGCATCCCAGTTTGAGACG) (1µl) and FAM labelled reverse (5' 6FAM-TACGCATCCCAGTTTGAGACGGAAGAAGAAGAAGAAGAA) (0.3µl) primers. The reactions were subjected to 1 cycle of 96°C for 5 min, and 30 cycles of 94°C for 1 min, 60°C for 1 min and 72°C for 2 min, followed by a final extension time at 72°C for 10 min. Products were separated on an ABI 3500 genetic analyzer (ThermoFisher Scientific). 4µl of PCR product was mixed with 0.3µl of LIZ-500 size standard (ThermoFisher) and 10µl of HiDi Formamide (ThermoFisher). The mixture was denatured at 94°C for 4 min then loaded onto the 3500 genetic analyzer. The data was analysed with Genemarker software (v2.2.0) where an expansion was visualised as a decremental saw-tooth pattern, and the size was measured against LIZ-500 ladder.

Allele sizes are calculated using internal size standard, from which GAA repeat size is obtained by subtracting size of non-repeat region from size of the PCR product divided by 3 (triplet repeat). Individuals beyond the threshold of 250 bp are considered as affected with myotonic dystrophy type 1.

Spinal-Bulbar Muscular Atrophy (SBMA) Kennedy's PCR (CDFD, India)

Short PCR: Genomic DNA was extracted from whole blood. 50 -100 ng of DNA was amplified in a 10µl total volume of Taq PCR Master Mix (5ul) (Qiagen), PCR grade water (2µl) and 10pmoles/µl of FAM labelled forward (5-6 FAM-TCC AGAATCTGTTCCAGAGCGTGC) and reverse (GCTGTGAAGGTTGCTGTTCCTCAT) primers (0.5µl each). The reactions were subjected to 1 cycle of 95°C for 5 min, and 30

cycles of Denaturation (95°C for 1 min), Annealing (95°C for 30 s), and Extension at 72°C for 1 min), with a final extension of 72°C for 10 min.

Products were separated on an ABI 3500 genetic analyzer (ThermoFisher Scientific). 4µl of PCR product was mixed with 0.3µl of LIZ-500 size standard (ThermoFisher) and 10µl of HiDi Formamide (ThermoFisher). The mixture was denatured at 94°C for 4 min then loaded onto an ABI 3500 genetic analyzer (ThermoFisher Scientific). The data was analysed with Genemarker software (v2.2.0) where an expansion was visualised as a decremental saw-tooth pattern, and the size was measured against LIZ-500 ladder.

Allele sizes are calculated using internal size standard, from which CAG repeat size is obtained by subtracting size of non-repeat region from size of the PCR product divided by 3 (triplet repeat).

FSHD genetic testing (Leiden University Medical Centre)

20 mls of blood-EDTA were collected from consenting participants. Blood samples were stored for between 1 and 6 weeks at 4°C prior to shipping in insulated packaging to UCL or Leiden for testing. Shipping packaging was selected to maintain cool temperatures and minimise risk of freezing in air transit. Date of sample collection was recorded to facilitate more accurate leukocyte number calculations based on pellet size, with older samples generating larger pellets due to cell size changes.

Isolated white blood cells were embedded in agarose plugs to obtain high molecular weight DNA as previously described[25]. DNA in agarose plugs was digested with informative restriction enzymes followed by pulsed field gel electrophoresis (PFGE), Southern blotting and sequential hybridization with radioactive labelled probes p13E-11, D4Z4, 4qA and 4qB. Finally, haplotype analysis was completed using the SSLP PCR as described previously[26]. For all patients with >7U D4Z4 repeat and having at least one 4qA chromosome, we determined the methylation at the FseI site in D4Z4 and calculated the delta1 methylation score to reveal differences in CpG methylation at D4Z4 that correlate with clinical variability in FSHD1 and FSHD2[26-28].

Sanger sequencing (UCL, UK)

Primers were designed using Primer3 (http://primer3.ut.ee/) to include approximately 100bp flanking intronic regions from the target DNA region (visualised on the Ensembl website).

Optimum primer size is usually around 20 BP, with optimal melting temperatures set for between 55°C and 65°C, the primer GC content is set at around 50 % (30-70), and low self-complementarity is preferable (avoiding primer dimers). Primers were ordered from Sigma already resuspended in autoclaved, double distilled MilliQ water. The stock solutions were diluted to a final working concentration of 10 pmol to be used in subsequent PCR. 50 ng of DNA was amplified in a 25µl total volume of Fast-start Master Mix (7.5µl, Merck), PCR grade water (5µl) and 10µM of forward and reverse primers. The reactions were subjected to 1 cycle of 94°C for 10 min, and 40 touch-down cycles of 94°C for 30 s, 65°C for 30 s and 72°C for 45 s, where in each cycle the annealing temperature slowly drops (eventually to 55°C), followed by a final extension time at 72°C for 5 min. The PCR products were electrophoresed on 2% agarose gel at 90 V for 35 min to check for the presence of a PCR product. Enzymatic clean-up was performed with 3µl of ExoSap-IT (ThermoFisher) and 7µl of the PCR product. Sanger sequencing was performed at Source Bioscience (Cambridge) and the sequences were aligned and analysed with Geneious Prime (version 2023.0.2)

References to Supplementary Data: Methods

1. Lappalainen I, Almeida-King J, Kumanduri V, et al. The European Genome-phenome Archive of human data consented for biomedical research. *Nat Genet* 2015; 47(7): 692-5.
2. Gainotti S, Torreri P, Wang CM, et al. The RD-Connect Registry & Biobank Finder: a tool for sharing aggregated data and metadata among rare disease researchers. *Eur J Hum Genet* 2018; 26(5): 631-43.
3. Harris PA, Taylor R, Minor BL, et al. The REDCap consortium: Building an international community of software platform partners. *J Biomed Inform* 2019; 95: 103208.
4. Harris PA, Taylor R, Thielke R, Payne J, Gonzalez N, Conde JG. Research electronic data capture (REDCap)--a metadata-driven methodology and workflow process for providing translational research informatics support. *J Biomed Inform* 2009; 42(2): 377-81.
5. Kohler S, Gargano M, Matentzoglu N, et al. The Human Phenotype Ontology in 2021. *Nucleic Acids Res* 2021; 49(D1): D1207-D17.
6. Balke B. A Simple Field Test for the Assessment of Physical Fitness. Rep 63-6. *Rep Civ Aeromed Res Inst US* 1963: 1-8.
7. Ramsey D, Scoto M, Mayhew A, et al. Revised Hammersmith Scale for spinal muscular atrophy: A SMA specific clinical outcome assessment tool. *PLoS One* 2017; 12(2): e0172346.
8. Padua L, Aprile I, Caliandro P, Pazzaglia C, Commodari I, Tonali P. Reliability and validity of the CMT neuropathy score as a measure of disability. *Neurology* 2006; 66(4): 614-5; author reply -5.
9. Burns J, Ouvrier R, Estilow T, et al. Validation of the Charcot-Marie-Tooth disease pediatric scale as an outcome measure of disability. *Ann Neurol* 2012; 71(5): 642-52.
10. Scott E, Eagle M, Mayhew A, et al. Development of a functional assessment scale for ambulatory boys with Duchenne muscular dystrophy. *Physiother Res Int* 2012; 17(2): 101-9.
11. Apgar V, Kreiselman J. Studies on Resuscitation - an Experimental Evaluation of the Bloxsom Air Lock. *Am J Obstet Gynecol* 1953; 65(1): 45-52.
12. Smedley D, Jacobsen JO, Jager M, et al. Next-generation diagnostics and disease-gene discovery with the Exomiser. *Nat Protoc* 2015; 10(12): 2004-15.
13. Li H, Durbin R. Fast and accurate short read alignment with Burrows-Wheeler transform. *Bioinformatics* 2009; 25(14): 1754-60.
14. Van der Auwera GA, Carneiro MO, Hartl C, et al. From FastQ data to high confidence variant calls: the Genome Analysis Toolkit best practices pipeline. *Curr Protoc Bioinformatics* 2013; 43(1110): 11 0 1- 0 33.
15. Pedersen BS, Quinlan AR. Who's Who? Detecting and Resolving Sample Anomalies in Human DNA Sequencing Studies with Peddy. *Am J Hum Genet* 2017; 100(3): 406-13.
16. McLaren W, Gil L, Hunt SE, et al. The Ensembl Variant Effect Predictor. *Genome Biol* 2016; 17(1): 122.
17. Karczewski KJ, Francioli LC, Tiao G, et al. The mutational constraint spectrum quantified from variation in 141,456 humans. *Nature* 2020; 581(7809): 434-43.
18. Choudhury A, Ramsay M, Hazelhurst S, et al. Whole-genome sequencing for an enhanced understanding of genetic variation among South Africans. *Nat Commun* 2017; 8(1): 2062.
19. Ramsay M, Crowther N, Tambo E, et al. H3Africa AWI-Gen Collaborative Centre: a resource to study the interplay between genomic and environmental risk factors for cardiometabolic diseases in four sub-Saharan African countries. *Glob Health Epidemiol Genom* 2016; 1: e20.
20. Martin AR, Williams E, Foulger RE, et al. PanelApp crowdsources expert knowledge to establish consensus diagnostic gene panels. *Nat Genet* 2019; 51(11): 1560-5.

21. Investigators GPP, Smedley D, Smith KR, et al. 100,000 Genomes Pilot on Rare-Disease Diagnosis in Health Care - Preliminary Report. *N Engl J Med* 2021; 385(20): 1868-80.
22. Thomas PK, Marques W, Jr., Davis MB, et al. The phenotypic manifestations of chromosome 17p11.2 duplication. *Brain* 1997; 120 (Pt 3): 465-78.
23. Warner JP, Barron LH, Goudie D, et al. A general method for the detection of large CAG repeat expansions by fluorescent PCR. *J Med Genet* 1996; 33(12): 1022-6.
24. Gacy AM, Goellner GM, Spiro C, et al. GAA instability in Friedreich's Ataxia shares a common, DNA-directed and intraallelic mechanism with other trinucleotide diseases. *Mol Cell* 1998; 1(4): 583-93.
25. van Overveld PG, Enthoven L, Ricci E, et al. Variable hypomethylation of D4Z4 in facioscapulohumeral muscular dystrophy. *Ann Neurol* 2005; 58(4): 569-76.
26. Lemmers RJ. Analyzing Copy Number Variation Using Pulsed-Field Gel Electrophoresis: Providing a Genetic Diagnosis for FSHD1. *Methods Mol Biol* 2017; 1492: 107-25.
27. Lemmers RJ, Goeman JJ, van der Vliet PJ, et al. Inter-individual differences in CpG methylation at D4Z4 correlate with clinical variability in FSHD1 and FSHD2. *Hum Mol Genet* 2015; 24(3): 659-69.
28. Rieken A, Bossler AD, Mathews KD, Moore SA. CLIA Laboratory Testing for Facioscapulohumeral Dystrophy: A Retrospective Analysis. *Neurology* 2021; 96(7): e1054-e62.
