# Supplementary material for: Neuromuscular disease genetics in under-represented populations: increasing data diversity
Source: Brain. 2023 Jul 30;146(12):5098–109. doi: 10.1093/brain/awad254 (PMC10690022; doi:10.1093/brain/awad254)
Supplement: awad254_Supplementary_Data [file awad254_supplementary_data.zip › brain-2023-00969-File012.pdf]

Supplementary Data: ICGNMD REDCap Database Instrument

ICGNMD Study Database

Consent obtained	◯ Consent by subject<br>◯ Consent by parent/guardian, advocate, or next of kin<br>◯ Other
Consent obtained - other	______________________
Consent taken by (must be authorised member of study team)	______________________<br>(Full name)
Consent Method Used	◯ In person (face to face)<br>◯ Postal Consent*<br>◯ Telephone Consent*<br>◯ Online Consent*<br>(*Only where permitted under local ethics)

After determination of eligibility and consent, assign and record ICGNMD study ID

ICGNMD study ID	______________________<br>(IC_[site code]_[participant number]; IC_XXX_XXXXX, e.g. IC_UTH_00001)
Other local study ID (optional)	______________________<br>(Other local study ID for participant (in addition to ICGNMD study ID))
Date consent form signed	______________________<br>(YYYY-MM-DD)

Participant type

Participant type	◯ Affected proband<br>◯ Affected relative<br>◯ Unaffected relative<br>◯ Unrelated control subject<br>(Clinical status)
Proband study ID (family grouping code)	______________________<br>(The ICGNMD study ID of the Proband, e.g. IC_UTH_00001)
Proband initials	______________________<br>(For data validation)
Proband year of birth	______________________<br>(For data validation)

Relation

Relationship to Proband

- ◯ Mother
- ◯ Father
- ◯ Son
- ◯ Daughter
- ◯ Grandchild
- ◯ Brother - non-identical
- ◯ Sister - non-identical
- ◯ Identical twin
- ◯ Paternal aunt/uncle
- ◯ Maternal aunt/uncle
- ◯ Paternal grandparent
- ◯ Maternal grandparent
- ◯ Paternal cousin
- ◯ Maternal cousin
- ◯ Niece/nephew
- ◯ Other

Relationship to Proband - other

____

Diagnosis

Diagnostic category

- ◯ Genetic motor neuron disease
- ◯ Spinal muscular atrophy
- ◯ Genetic peripheral neuropathy
- ◯ Congenital myasthenic syndrome
- ◯ Congenital muscular dystrophy or myopathy
- ◯ Duchenne or Becker muscular dystrophy
- ◯ Facioscapulohumeral muscular dystrophy
- ◯ Myotonic dystrophy type I
- ◯ Limb girdle muscular dystrophy
- ◯ Distal myopathy
- ◯ Adult onset myopathy
- ◯ Metabolic myopathy
- ◯ Mitochondrial disease
- ◯ Skeletal muscle channelopathy
- ◯ Undetermined genetic neuromuscular or neurological disorder

(Clinical diagnosis most likely, in opinion of researcher)

Confirmed diagnosis

____
(Orphanet)

Other confirmed diagnosis - 1

____
(Orphanet)

Other confirmed diagnosis - 2

____
(Orphanet)

Confirmed diagnoses - freetext

____
(Offline entry)

Age clinically diagnosed - years	______________________
Age clinically diagnosed - months	______________________

Pre-existing samples available

Pre-existing samples available for genetic analysis	◯ No<br>◯ Yes<br>◯ Unknown
DNA	◯ No<br>◯ Yes<br>◯ Unknown
Blood	◯ No<br>◯ Yes<br>◯ Unknown
Skin tissue/fibroblast	◯ No<br>◯ Yes<br>◯ Unknown
Muscle tissue	◯ No<br>◯ Yes<br>◯ Unknown
Buccal	◯ No<br>◯ Yes<br>◯ Unknown
Saliva	◯ No<br>◯ Yes<br>◯ Unknown
Urine	◯ No<br>◯ Yes<br>◯ Unknown

New samples collected

New samples collected at study visit	◯ No<br>◯ Yes<br>◯ Unknown
DNA	◯ No<br>◯ Yes<br>◯ Unknown
Blood	◯ No<br>◯ Yes<br>◯ Unknown

Skin tissue/fibroblast	◯ No<br>◯ Yes<br>◯ Unknown
Muscle tissue	◯ No<br>◯ Yes<br>◯ Unknown
Buccal	◯ No<br>◯ Yes<br>◯ Unknown
Saliva	◯ No<br>◯ Yes<br>◯ Unknown
Urine	◯ No<br>◯ Yes<br>◯ Unknown

Demographics

Current age - years	__________________________
Current age - months	__________________________
Sex	◯ Male<br>◯ Female<br>(Biological sex at birth)
Proband year of birth	__________________________

Ethnicity (self-reported)

Country of birth	◯ Brazil<br>◯ India<br>◯ South Africa<br>◯ Turkey<br>◯ United Kingdom<br>◯ Zambia<br>◯ Other
Country of birth - other	__________________________
Region/province of birth	__________________________
Main language	__________________________

Ethnicity

____
(Please describe the ethnic origin)

Ethnicity - subgroup

____
((if applicable))

Father's ethnicity

____
(Please describe the paternal ethnic origin)

Father's ethnicity - subgroup

____
((if applicable))

Mother's ethnicity

____
(Please describe the maternal ethnic origin)

Mother's ethnicity - subgroup

____
((if applicable))

Brazil

Ethnicity

- ◯ White
- ◯ Amerindians
- ◯ Pardo-Brazilians
- ◯ African-Brazilians
- ◯ Asian-Brazilians
- ◯ Other

Ethnicity - other

____
(Please describe the ethnic origin)

Ethnicity - subgroup

____
((if applicable))

Father's ethnicity

- ◯ White
- ◯ Amerindians
- ◯ Pardo-Brazilians
- ◯ African-Brazilians
- ◯ Asian-Brazilians
- ◯ Other

Father's ethnicity - other

____
(Please describe the paternal ethnic origin)

Father's ethnicity - subgroup

____
((if applicable))

Mother's ethnicity	◯ White<br>◯ Amerindians<br>◯ Pardo-Brazilians<br>◯ African-Brazilians<br>◯ Asian-Brazilians<br>◯ Other
Mother's ethnicity - other	__________________________________<br>(Please describe the maternal ethnic origin)
Mother's ethnicity - subgroup	__________________________________<br>((if applicable))

India

Ethnicity - tribe	__________________________________
Ethnicity - other	__________________________________<br>(Please describe the ethnic origin)
Ethnicity - subgroup	__________________________________<br>((if applicable))
Father's ethnicity - tribe	__________________________________
Father's ethnicity - other	__________________________________<br>(Please describe the paternal ethnic origin)
Father's ethnicity - subgroup	__________________________________<br>((if applicable))
Mother's ethnicity - tribe	__________________________________
Mother's ethnicity - other	__________________________________<br>(Please describe the maternal ethnic origin)
Mother's ethnicity - subgroup	__________________________________<br>((if applicable))

South Africa

Ethnicity	◯ Black African<br>◯ Mixed ancestry<br>◯ White<br>◯ Indian/Asian<br>◯ Other
Ethnicity - other	__________________________________<br>(Please describe the ethnic origin)
Ethnicity - subgroup	__________________________________<br>((if applicable))
Father's ethnicity	◯ Black African<br>◯ Mixed ancestry<br>◯ White<br>◯ Indian/Asian<br>◯ Other
Father's ethnicity - other	__________________________________<br>(Please describe the paternal ethnic origin)
Father's ethnicity - subgroup	__________________________________<br>((if applicable))
Mother's ethnicity	◯ Black African<br>◯ Mixed ancestry<br>◯ White<br>◯ Indian/Asian<br>◯ Other
Mother's ethnicity - other	__________________________________<br>(Please describe the maternal ethnic origin)
Mother's ethnicity - subgroup	__________________________________<br>((if applicable))

Turkey

Ethnicity	◯ Turkish<br>◯ Kurdish<br>◯ Other
Ethnicity - other	__________________________________<br>(Please describe the ethnic origin)
Ethnicity - subgroup	__________________________________<br>((if applicable))

Father's ethnicity	◯ Turkish<br>◯ Kurdish<br>◯ Other
Father's ethnicity - other	__________________________________<br>(Please describe the paternal ethnic origin)
Father's ethnicity - subgroup	__________________________________<br>((if applicable))
Mother's ethnicity	◯ Turkish<br>◯ Kurdish<br>◯ Other
Mother's ethnicity - other	__________________________________<br>(Please describe the maternal ethnic origin)
Mother's ethnicity - subgroup	__________________________________<br>((if applicable))

United Kingdom

Ethnicity	◯ White British<br>◯ White Irish<br>◯ Gypsy or Irish Traveller<br>◯ Any other White background<br>◯ White and Black Caribbean<br>◯ White and Black African<br>◯ White and Asian<br>◯ Any other Mixed / Multiple ethnic background<br>◯ Indian<br>◯ Pakistani<br>◯ Bangladeshi<br>◯ Chinese<br>◯ Any other Asian background<br>◯ African<br>◯ Caribbean<br>◯ Any other Black / African / Caribbean background<br>◯ Arab<br>◯ Any other ethnic group<br>◯ Unknown
Ethnicity - other	__________________________________<br>(Please describe the ethnic origin)
Ethnicity - subgroup	__________________________________<br>((if applicable))

Father's ethnicity	◯ White British<br>◯ White Irish<br>◯ Gypsy or Irish Traveller<br>◯ Any other White background<br>◯ White and Black Caribbean<br>◯ White and Black African<br>◯ White and Asian<br>◯ Any other Mixed / Multiple ethnic background<br>◯ Indian<br>◯ Pakistani<br>◯ Bangladeshi<br>◯ Chinese<br>◯ Any other Asian background<br>◯ African<br>◯ Caribbean<br>◯ Any other Black / African / Caribbean background<br>◯ Arab<br>◯ Any other ethnic group<br>◯ Unknown
Father's ethnicity - other	__________________________________<br>(Please describe the paternal ethnic origin)
Father's ethnicity - subgroup	__________________________________<br>((if applicable))
Mother's ethnicity	◯ White British<br>◯ White Irish<br>◯ Gypsy or Irish Traveller<br>◯ Any other White background<br>◯ White and Black Caribbean<br>◯ White and Black African<br>◯ White and Asian<br>◯ Any other Mixed / Multiple ethnic background<br>◯ Indian<br>◯ Pakistani<br>◯ Bangladeshi<br>◯ Chinese<br>◯ Any other Asian background<br>◯ African<br>◯ Caribbean<br>◯ Any other Black / African / Caribbean background<br>◯ Arab<br>◯ Any other ethnic group<br>◯ Unknown
Mother's ethnicity - other	__________________________________<br>(Please describe the maternal ethnic origin)
Mother's ethnicity - subgroup	__________________________________<br>((if applicable))

Zambia

Ethnicity	◯ Black African<br>◯ Mixed ancestry<br>◯ White<br>◯ Indian/Asian<br>◯ Other
Ethnicity - other	__________________________________<br>(Please describe the ethnic origin)
Ethnicity - subgroup	__________________________________<br>((if applicable))
Father's ethnicity	◯ Black African<br>◯ Mixed ancestry<br>◯ White<br>◯ Indian/Asian<br>◯ Other
Father's ethnicity - other	__________________________________<br>(Please describe the paternal ethnic origin)
Father's ethnicity - subgroup	__________________________________<br>((if applicable))
Mother's ethnicity	◯ Black African<br>◯ Mixed ancestry<br>◯ White<br>◯ Indian/Asian<br>◯ Other
Mother's ethnicity - other	__________________________________<br>(Please describe the maternal ethnic origin)
Mother's ethnicity - subgroup	__________________________________<br>((if applicable))

Known genes in VCF format - please use GRCh38 (e.g. 1 230710048 rs699 A G)

Known gene mutation - VCF	__________________________________<br>(1 230710048 rs699 A G)
Known gene mutation - OMIM	__________________________________<br>(Optional)
Second known gene mutation - VCF	__________________________________<br>(1 230710048 rs699 A G)

Second known gene mutation - OMIM

____
(Optional)

Third known gene mutation - VCF

____
(1 230710048 rs699 A G)

Third known gene mutation - OMIM

____
(Optional)

Fourth known gene mutation - VCF

____
(1 230710048 rs699 A G)

Fourth known gene mutation - OMIM

____
(Optional)

Fifth known gene mutation - VCF

____
(1 230710048 rs699 A G)

Fifth known gene mutation - OMIM

____
(Optional)

Known mutation(s) - freetext

____

Family history

Family history of core disease

- ◯ Absent
- ◯ Present
- ◯ Unknown

Family history of core disease - details

____

Consanguinity

- ◯ Absent
- ◯ Present
- ◯ Unknown

Consanguinity - description

____
(E.g. 1st, 2nd, 3rd cousin)

Known mutation(s) in family - freetext

____
(Offline entry)

Upload pedigree	(Online tool for drawing pedigree: https://www.progenygenetics.com/online-pedigree/)

Inheritance

Inheritance	◯ Autosomal dominant<br>◯ Autosomal recessive<br>◯ X-linked dominant<br>◯ X-linked recessive<br>◯ Matrilineal<br>◯ Familial (unspecified inheritance)<br>◯ Idiopathic/sporadic<br>◯ Unknown

Symptoms

First symptom noticed by subject or parents/carers	__________________________________
First symptom noticed by subject or parents/carers - freetext	__________________________________<br>(Offline entry)
Age of onset symptoms - years	__________________________________<br>(If present at birth, enter 0 as value)
Age of onset symptoms - months	__________________________________<br>(If present at birth, enter 0 as value)

Disease type

Progression	◯ Progressive<br>◯ Non-progressive<br>◯ Other or unspecified<br>◯ Unknown
Global pace of progression	◯ Days<br>◯ Months<br>◯ Years
Characteristics	◯ Typical<br>◯ Atypical<br>◯ Other or unspecified<br>◯ Unknown
Persistence	◯ Chronic<br>◯ Episodic/intermittent<br>◯ Other or unspecified<br>◯ Unknown
Complexity	◯ Pure (uncomplicated)<br>◯ Complicated<br>◯ Other or unspecified<br>◯ Unknown

Disease type - comments ______________________

Present phenotypic features (HPO) - please enter at least five

Present feature - 1 ______________________

Present feature - 2 ______________________

Present feature - 3 ______________________

Present feature - 4 ______________________

Present feature - 5 ______________________

Present feature - 6 ______________________

Present feature - 7 ______________________

Present feature - 8 ______________________

Present feature - 9 ______________________

Present feature - 10 ______________________

Present feature - 11 ______________________

Present feature - 12 ______________________

Present feature - 13 ______________________

Present feature - 14 ______________________

Present feature - 15 ______________________

Present feature - 16 ______________________

Present feature - 17 ______________________

Present feature - 18

____

Present feature - 19

____

Present features - freetext

____

(Offline entry)

Absent phenotypic features (HPO) - please enter at least five

Absent feature - 1

____

Absent feature - 2

____

Absent feature - 3

____

Absent feature - 4

____

Absent feature - 5

____

Absent feature - 6

____

Absent feature - 7

____

Absent feature - 8

____

Absent feature - 9

____

Absent feature - 10

____

Absent feature - 11

____

Absent feature - 12

____

Absent feature - 13

____

Absent feature - 14

____

Absent feature - 15

____

Absent feature - 16

____

Absent feature - 17

____

Absent feature - 18

____

Absent feature - 19

____

Absent features - freetext

____
(Offline entry)

Comments

Any other comments on core data

____

Other Medical History

Handedness

Handedness

◯ Left-handed
◯ Right-handed
◯ Both hands/Ambidextrous
◯ Unknown
(The hand used predominately; not necessarily the hand used for writing with exclusively)

Smoking history

Smoking status

◯ Current tobacco/cigarette smoker (including non-daily smoker)
◯ Ex-smoker
◯ Never smoker (not smoked for more than a year in total)
◯ Unknown

Frequency of smoking

◯ Non-daily
◯ Daily

Age smoking regularly

____
(At what age (years) did the subject start smoking regularly?)

Cigarette consumption / day

____
(If the subject smoked a pipe, rolling tobacco, cigar or similar then ** g of tobacco = 1 cigarette)

Age quit smoking

____
(At what age (years) did the subject quit smoking?)

Smoking - comments

____
(Any other comments on smoking history. Also insert any comments about (i) chewing tobacco and/or (ii) betel leaf/areca nut use here.)

Alcohol history

Does the participant currently consume alcohol? (within the past year)

◯ No
◯ Yes
◯ Unknown

Has the participant consumed alcohol in the past? (prior to the past year)

◯ No
◯ Yes
◯ Unknown

At what age did the participant start consuming alcohol?	______________________
At what age did the participant stop consuming alcohol (if applicable)?	______________________
How often does the participant consume alcohol? If the subject no longer consumes alcohol, how often did they previously consume alcohol?	◯ 4 or more times per week<br>◯ 2-3 times per week<br>◯ 2-4 times per month<br>◯ Monthly or less<br>◯ N/A
How many units does the participant consume on a typical day when drinking? If the participant no longer consumes alcohol, how many units did they previously consume?	◯ 1-2<br>◯ 3-4<br>◯ 5-6<br>◯ 7-9<br>◯ 10+<br>◯ N/A
How often does the participant have 6 or more alcoholic beverages on one occasion? If the participant no longer consumes alcohol, how often did they previously consume 6 or more alcoholic beverages on one occasion?	◯ Daily or almost daily<br>◯ Weekly<br>◯ Monthly<br>◯ Less than monthly<br>◯ Never
Has the participant ever been hospitalised for any alcohol-related problem? (e.g. oesophageal varices, delirium, tremors, cirrhosis, etc.)	◯ No<br>◯ Yes<br>◯ Unknown
Hospitalisation for alcohol - description	______________________<br>(Please describe)

Medication

Medication(s) taken	______________________<br>(Start/end dates. Generic drug name(s) and mean daily dosage(s) in mg.)
Describe other treatment given	______________________

Prenatal, perinatal, or early developmental history

Prenatal, perinatal, or early developmental history available	◯ No<br>◯ Yes

Prenatal history

In-utero movements	◯ Normal<br>◯ Reduced<br>◯ Unknown
Polyhydramnios	◯ No<br>◯ Yes<br>◯ Unknown
Maternal risk factors	__________________________________

Perinatal history

Gestation period in weeks	__________________________________
Birth - delivery	◯ Normal<br>◯ C-section (elective)<br>◯ C-section (emergency)<br>◯ Assisted (ventouse/forceps)
APGAR score(s) available?	◯ No<br>◯ Yes<br>◯ Unknown
APGAR - 1 minute - score	__________________________________<br>((0-10))
APGAR - 5 minutes - score	__________________________________<br>((0-10))
APGAR - 10 minutes - score	__________________________________<br>((0-10))
Birth length in centimetres	__________________________________
Birth weight in kg	__________________________________
Birth head circumference in centimetres	__________________________________
Neonatal complications	◯ No<br>◯ Yes<br>◯ Unknown

Neonatal complications - type	☐ Contractures<br>☐ Fractures<br>☐ Poor feeding<br>☐ Neonatal encephalopathy<br>☐ Required ventilatory support<br>☐ Other
Neonatal encephalopathy - severity	○ Mild<br>○ Moderate<br>○ Severe

Infancy and early childhood developmental history

Motor development normal or abnormal	○ Normal<br>○ Abnormal<br>○ Unknown
Age at sitting - months	__________________________________<br>(If never developed, enter 0)
Age at crawling - months	__________________________________<br>(If never developed, enter 0)
Age at walking - months	__________________________________<br>(If never developed, enter 0)
Speech and hearing development	○ Normal<br>○ Abnormal<br>○ Unknown
Age at first words - months	__________________________________<br>(If never developed, enter 0)
Age using two-word phrases - months	__________________________________<br>(If never developed, enter 0)
Social development	○ Normal<br>○ Abnormal<br>○ Unknown
Age overcoming stranger anxiety - months	__________________________________<br>(If never developed, enter 0)
Age waving bye bye - months	__________________________________<br>(If never developed, enter 0)

Past medical history

Past medical history

____
(Including any other medical conditions and details of any environmental toxin exposure)

Past surgical history

Past surgical history

____

Comments

Any other comments on other medical history

____

Clinical Assessment

Examination normal or abnormal	◯ Normal<br>◯ Abnormal<br>◯ Unknown
Have any aspects of patient examination been video recorded or photographed?	◯ No<br>◯ Yes<br>◯ Unknown
If yes, which aspects	☐ Gait<br>☐ Abnormal movements<br>☐ Seizures<br>☐ Cranial nerve examiation<br>☐ Limb examination - motor<br>☐ Limb examination - sensory<br>☐ Other
Please describe other aspect(s)	__________________________

Cardiac dysfunction

Cardiac dysfunction	◯ No<br>◯ Yes<br>◯ Unknown
Cardiac dysfunction - type	☐ Conduction defects<br>☐ Cardiomyopathy<br>☐ Other
Cardiac dysfunction - type - other	__________________________
Cardiomyopathy - type	◯ Dilated cardiomyopathy<br>◯ Hypertrophic cardiomyopathy<br>◯ Other
Cardiomyopathy type - other	__________________________
Age at onset of cardiac dysfunction - years	__________________________<br>(If present at birth, enter 0 as value)
Age at onset of cardiac dysfunction - months	__________________________<br>(If present at birth, enter 0 as value)
Cardiac intervention	◯ No<br>◯ Yes<br>◯ Unknown

Cardiac intervention - type	☐ Pacemaker<br>☐ ICD<br>☐ Cardiac transplant<br>☐ Other
Cardiac intervention - type - other	______________________
Age at intervention - years	______________________
Age at intervention - months	______________________

Respiratory dysfunction

Respiratory dysfunction	◯ No<br>◯ Yes<br>◯ Unknown
Respiratory dysfunction - type	☐ Neuromuscular<br>☐ Cardiac<br>☐ Pulmonary<br>☐ Central<br>☐ Other
Respiratory dysfunction - other	______________________
Age at onset of respiratory dysfunction - years	______________________<br>(If present at birth, enter 0 as value)
Age at onset of respiratory dysfunction - months	______________________<br>(If present at birth, enter 0 as value)
FVC tested	◯ No<br>◯ Yes<br>◯ Unknown
FVC tested - value - 1	______________________<br>(% of predicted value)
FVC tested - age in years - 1	______________________<br>(Years)
FVC tested - position - 1	◯ Sitting<br>◯ Lying
FVC tested - value - 2	______________________<br>(% of predicted value)

FVC tested - age in years - 2	______________________ (Years)
FVC tested - position - 2	◯ Sitting<br>◯ Lying
FVC tested - value - 3	______________________ (% of predicted value)
FVC tested - age in years - 3	______________________ (Years)
FVC tested - position - 3	◯ Sitting<br>◯ Lying
PEFR tested	◯ No<br>◯ Yes<br>◯ Unknown
PEFR tested - value - 1	______________________ (% of predicted value)
PEFR tested - age in years - 1	______________________ (Years)
PEFR tested - position - 1	◯ Sitting<br>◯ Lying
PEFR tested - value - 2	______________________ (% of predicted value)
PEFR tested - age in years - 2	______________________ (Years)
PEFR tested - position - 2	◯ Sitting<br>◯ Lying
PEFR tested - value - 3	______________________ (% of predicted value)
PEFR tested - age in years - 3	______________________ (Years)
PEFR tested - position - 3	◯ Sitting<br>◯ Lying

Artificial ventilation	◯ No<br>◯ Yes<br>◯ Unknown<br>(Ever used)
Daily duration of artificial ventilation	◯ All day<br>◯ Day only<br>◯ Night only<br>◯ Only with infections<br>◯ Unknown
Artificial ventilation - type	◯ NIV<br>◯ CPAP<br>◯ Tracheostomy<br>◯ Other
Artificial ventilation type - other	______________________
Artificial ventilation started age - years	______________________
Artificial ventilation started age - months	______________________
Artificial ventilation stopped age - years	______________________<br>(If applicable)
Artificial ventilation stopped age - months	______________________<br>(If applicable)

Seizures

Seizures	◯ No<br>◯ Yes<br>◯ Unknown
Seizures - type	◯ Focal onset<br>◯ Generalised onset<br>◯ Unknown onset
Seizures - age at onset - years	______________________<br>(If present at birth, enter 0 as value)
Seizures - age at onset - months	______________________<br>(If present at birth, enter 0 as value)

Movement disorder

Movement disorder	◯ No<br>◯ Yes<br>◯ Unknown
Movement disorder - type	☐ Cerebellar ataxia<br>☐ Chorea<br>☐ Dystonia<br>☐ Tremor<br>☐ Other
Movement disorder type - other	______________________
Movement disorder - distribution	☐ UL proximal<br>☐ UL distal<br>☐ LL proximal<br>☐ LL distal<br>☐ Ocular<br>☐ Facial<br>☐ Oromandibular<br>☐ Cervical
Movement disorder - age at onset - years	______________________<br>(If present at birth, enter 0 as value)
Movement disorder - age at onset - months	______________________<br>(If present at birth, enter 0 as value)

Autonomic dysfunction

Autonomic dysfunction	◯ No<br>◯ Yes<br>◯ Unknown
Autonomic dysfunction - type	☐ Hyperhidosis<br>☐ Constipation<br>☐ Cardiac dysfunction<br>☐ Bladder dysfunction<br>☐ Erectile dysfunction<br>☐ Other
Autonomic dysfunction type - other	______________________
Autonomic dysfunction - age at onset - years	______________________<br>(If present at birth, enter 0 as value)
Autonomic dysfunction - age at onset - months	______________________<br>(If present at birth, enter 0 as value)

Cognitive

Cognitive dysfunction	◯ No<br>◯ Yes<br>◯ Unknown
Cognitive dysfunction - type	☐ Global developmental delay (< 5 years)<br>☐ Intellectual disability (> 5 years)<br>☐ Learning disabilities<br>☐ Dementia<br>☐ Behavioural change<br>☐ Other
Learning disabilities - type	☐ Dyscalculea<br>☐ Dyspraxia<br>☐ Dyslexia
Cognitive dysfunction type - other	__________________________________
Cognitive dysfunction - course	◯ Static<br>◯ Improving<br>◯ Regression<br>◯ Unknown
Cognitive dysfunction - age at onset - years	__________________________________<br>(If present at birth, enter 0 as value)
Cognitive dysfunction - age at onset - months	__________________________________<br>(If present at birth, enter 0 as value)
Neuropsychometry performed	◯ No<br>◯ Yes<br>◯ Unknown
Neuropsychometry - report	(Upload/view Neuropsychometry report)
Neuropsychiatric disorders	◯ No<br>◯ Yes<br>◯ Unknown
Neuropsychiatric disorders- type	☐ Autistic spectrum disorder<br>☐ ADHD<br>☐ Psychosis<br>☐ Depression<br>☐ Other
Neuropsychiatric disorders - type - other	__________________________________
Neuropsychiatric disorders - age at onset - years	__________________________________<br>(If present at birth, enter 0 as value)

Neuropsychiatric disorders - age at onset - months

______________________ (If present at birth, enter 0 as value)

Gait

Motor ability

- ◯ Normal for age
- ◯ Walks without restrictions, with limitations for running and jumping
- ◯ Walks in most settings, may use physical assistance and climb stairs holding onto a railing
- ◯ Walks using a hand-held mobility device, use wheeled mobility for long distances
- ◯ Use methods of mobility that require physical assistance or powered mobility in most settings
- ◯ Transported in a manual wheelchair in all settings

(Gross motor function)

Diminished motor ability - age at onset - years

______________________ (If present at birth, enter 0 as value)

Diminished motor ability - age at onset - months

______________________ (If present at birth, enter 0 as value)

Lost ambulation - age at onset - years

______________________ (If present at birth, enter 0 as value)

Lost ambulation - age at onset - months

______________________ (If present at birth, enter 0 as value)

6 min walk test - distance in metres

____

Dysmorphic features

Dysmorphic features

- ◯ No
- ◯ Yes
- ◯ Unknown

Dysmorphic features - description

____

Dysmorphic features - age of onset - years

______________________ (If present at birth, enter 0 as value)

Dysmorphic features - age of onset - months

______________________ (If present at birth, enter 0 as value)

Dysmorphic features - photo

(Upload/view Photo of dysmorphic features)

Cranial nerves

Visual acuity	◯ Normal<br>◯ Abnormal<br>◯ Unknown
Visual acuity - right eye	____________________<br>(E.g. "6/6" (Snellen), "0.0" (LogMAR) or "20/20")
Visual acuity - left eye	____________________<br>(E.g. "6/6" (Snellen), "0.0" (LogMAR) or "20/20")
Visual impairment	◯ No<br>◯ Yes<br>◯ Unknown
Visual impairment - type	◯ Sight impaired<br>◯ Severely sight impaired or blind
Ocular signs	◯ No<br>◯ Yes<br>◯ Unknown
Ocular signs - type	☐ Ptosis<br>☐ Opthalmoparesis<br>☐ INO<br>☐ Gaze palsy<br>☐ Nystagmus<br>☐ Cataracts<br>☐ Glaucoma<br>☐ Optic atrophy<br>☐ Structural eye abnormalities<br>☐ Squint<br>☐ Other
Ocular signs type - other	____________________
Ocular signs - age at onset - years	____________________<br>(If present at birth, enter 0 as value)
Ocular signs - age at onset - months	____________________<br>(If present at birth, enter 0 as value)
Facial weakness	◯ No<br>◯ Yes<br>◯ Unknown
Facial weakness - age at onset - years	____________________<br>(If present at birth, enter 0 as value)

Facial weakness - age at onset - months	________________________________<br>(If present at birth, enter 0 as value)
Bulbar signs	◯ No<br>◯ Yes<br>◯ Unknown
Bulbar signs - type	☐ Dysarthria<br>☐ Dysphagia<br>☐ Brisk jaw jerk<br>☐ Tongue fasciculations<br>☐ Tongue stiff<br>☐ Tongue weak<br>☐ Tongue large<br>☐ Neck weakness<br>☐ Other
Bulbar signs type - other	________________________________
Bulbar signs - age at onset - years	________________________________<br>(If present at birth, enter 0 as value)
Bulbar signs - age at onset - months	________________________________<br>(If present at birth, enter 0 as value)
Percutaneous Endoscopic Gastrostomy (PEG)	◯ No<br>◯ Yes<br>◯ Refused<br>◯ Unknown<br>(Ever used)
Feeding orally with PEG?	◯ No<br>◯ Yes<br>◯ Unknown
Percutaneous Endoscopic Gastrostomy (PEG) - age at insertion - years	________________________________
Percutaneous Endoscopic Gastrostomy (PEG) - age at insertion - months	________________________________
Percutaneous Endoscopic Gastrostomy (PEG) - age at removal - years	________________________________<br>(If applicable)
Percutaneous Endoscopic Gastrostomy (PEG) - age at removal - months	________________________________<br>(If applicable)
Nasogastric (NG) tube	◯ No<br>◯ Yes<br>◯ Unknown<br>(Ever used)

Nasogastric (NG) tube - age at insertion - years	________________
Nasogastric (NG) tube - age at insertion - months	________________
Nasogastric (NG) tube - age at removal - years	________________ (If applicable)
Nasogastric (NG) tube - age at removal - months	________________ (If applicable)
Hearing loss	◯ No<br>◯ Yes<br>◯ Unknown
Hearing loss - type	◯ Conductive<br>◯ Sensorineural<br>◯ Unknown
Hearing loss - age of onset - years	________________ (If present at birth, enter 0 as value)
Hearing loss - age of onset - months	________________ (If present at birth, enter 0 as value)

Limbs and trunk

Scapular winging	◯ No<br>◯ Yes<br>◯ Unknown
Scapular winging - distribution	◯ Left<br>◯ Right<br>◯ Bilateral
Scapular winging - age at onset - years	________________ (If present at birth, enter 0 as value)
Scapular winging - age at onset - months	________________ (If present at birth, enter 0 as value)
Spinal abnormalities	◯ No<br>◯ Yes<br>◯ Unknown
Spinal abnormalities - type	☐ Kyphosis<br>☐ Scoliosis<br>☐ Hyperlordosis

Spinal abnormalities - age at onset - years	______________________ (If present at birth, enter 0 as value)
Spinal abnormalities - age at onset - months	______________________ (If present at birth, enter 0 as value)
Contractures	◯ No<br>◯ Yes<br>◯ Unknown
Contractures - distribution	☐ UL<br>☐ LL<br>☐ Axial/rigid spine
Contractures distribution - UL joints	☐ Left fingers<br>☐ Right fingers<br>☐ Left wrist<br>☐ Right wrist<br>☐ Left elbow<br>☐ Right elbow<br>☐ Left shoulder<br>☐ Right shoulder
Contractures distribution - LL joints	☐ Left toes<br>☐ Right toes<br>☐ Left ankle<br>☐ Right ankle<br>☐ Left knee<br>☐ Right knee<br>☐ Left hip<br>☐ Right hip
Contractures - age at onset - years	______________________ (If present at birth, enter 0 as value)
Contractures - age at onset - months	______________________ (If present at birth, enter 0 as value)
Joint laxity/hypermobility	◯ No<br>◯ Yes<br>◯ Unknown
Joint laxity/hypermobility - distribution	☐ UL<br>☐ LL<br>☐ Axial
Joint laxity/hypermobility distribution - UL joints	☐ Left fingers<br>☐ Right fingers<br>☐ Left wrist<br>☐ Right wrist<br>☐ Left elbow<br>☐ Right elbow<br>☐ Left shoulder<br>☐ Right shoulder

Joint laxity/hypermobility distribution - LL joints	☐ Left toes<br>☐ Right toes<br>☐ Left ankle<br>☐ Right ankle<br>☐ Left knee<br>☐ Right knee<br>☐ Left hip<br>☐ Right hip
Joint laxity/hypermobility - age at onset - years	__________________<br>(If present at birth, enter 0 as value)
Joint laxity/hypermobility - age at onset - months	__________________<br>(If present at birth, enter 0 as value)
Webbing/pterygium	◯ No<br>◯ Yes<br>◯ Unknown
Webbing/pterygium - distribution	☐ Hands<br>☐ Feet
Pes cavus	◯ No<br>◯ Yes<br>◯ Unknown
Pes cavus - age at onset - years	__________________<br>(If present at birth, enter 0 as value)
Pes cavus - age at onset - months	__________________<br>(If present at birth, enter 0 as value)
Pes planus	◯ No<br>◯ Yes<br>◯ Unknown
Pes planus - age at onset - years	__________________<br>(If present at birth, enter 0 as value)
Pes planus - age at onset - months	__________________<br>(If present at birth, enter 0 as value)
Equinovarus	◯ No<br>◯ Yes<br>◯ Unknown
Equinovarus - age at onset - years	__________________<br>(If present at birth, enter 0 as value)

Equinovarus - age at onset - months

____
(If present at birth, enter 0 as value)

Amputation(s)

- ◯ No
- ◯ Yes
- ◯ Unknown

Amputation - which limb(s) or toe(s)

____

Ulcers

- ◯ No
- ◯ Yes
- ◯ Unknown

Ulcers - distribution

- ☐ UL
- ☐ LL

Ulcers - age at onset - years

____
(If present at birth, enter 0 as value)

Ulcers - age at onset - months

____
(If present at birth, enter 0 as value)

Muscle bulk

- ◯ Normal
- ◯ Abnormal
- ◯ Unknown

Muscle atrophy/wasting

- ◯ No
- ◯ Yes
- ◯ Unknown

Muscle atrophy/wasting - distribution

- ☐ UL proximal
- ☐ UL distal
- ☐ LL proximal
- ☐ LL distal
- ☐ Axial

Muscle atrophy/wasting - age at onset - years

____
(If present at birth, enter 0 as value)

Muscle atrophy/wasting - age at onset - months

____
(If present at birth, enter 0 as value)

Muscle hypertrophy

- ◯ No
- ◯ Yes
- ◯ Unknown

Muscle hypertrophy - distribution	☐ UL proximal<br>☐ UL distal<br>☐ LL proximal<br>☐ LL distal<br>☐ Neck<br>☐ Facial<br>☐ Paraspinal<br>☐ Scapular
Muscle hypertrophy - age at onset - years	______________________________<br>(If present at birth, enter 0 as value)
Muscle hypertrophy - age at onset - months	______________________________<br>(If present at birth, enter 0 as value)
Muscle weakness	◯ No<br>◯ Yes<br>◯ Unknown
Muscle weakness - distribution	☐ UL proximal<br>☐ UL distal<br>☐ LL proximal<br>☐ LL distal<br>☐ Axial<br>☐ Symmetrical<br>☐ Asymmetrical
Muscle weakness - LL proximal compartment(s)	☐ Anterior compartment<br>☐ Posterior compartment
Muscle weakness - LL distal compartment(s)	☐ Anterior compartment<br>☐ Posterior compartment
Muscle weakness - age at onset - years	______________________________<br>(If present at birth, enter 0 as value)
Muscle weakness - age at onset - months	______________________________<br>(If present at birth, enter 0 as value)
Fatigability	◯ No<br>◯ Yes<br>◯ Unknown
Fatigability - type	◯ Exertion<br>◯ Diurnal
Fatigability - distribution	☐ UL<br>☐ LL<br>☐ Ptosis<br>☐ Extraocular<br>☐ Cervical<br>☐ Bulbar

Fatigability - age at onset - years	__________________________________ (If present at birth, enter 0 as value)
Fatigability - age at onset - months	__________________________________ (If present at birth, enter 0 as value)
Myotonia	◯ No<br>◯ Yes<br>◯ Unknown
Myotonia - distribution	☐ UL proximal<br>☐ UL distal<br>☐ LL proximal<br>☐ LL distal<br>☐ Eye closure<br>☐ Facial<br>☐ Cervical<br>☐ Paraspinal
Myotonia - age at onset - years	__________________________________ (If present at birth, enter 0 as value)
Myotonia - age at onset - months	__________________________________ (If present at birth, enter 0 as value)
Peripheral/lower motor neuron signs	◯ No<br>◯ Yes<br>◯ Unknown
Peripheral/lower motor neuron signs - type	☐ Reduced tone<br>☐ Reduced/absent reflexes
Peripheral/lower motor neuron signs reduced tone - distribution	☐ RUL<br>☐ LUL<br>☐ RLL<br>☐ LLL
Peripheral/lower motor neuron signs reduced/absent reflexes- distribution	☐ RUL<br>☐ LUL<br>☐ RLL<br>☐ LLL
Peripheral/lower motor neuron signs reduced/absent reflexes distribution - RUL	☐ Bicep<br>☐ Supinator<br>☐ Tricep
Peripheral/lower motor neuron signs reduced/absent reflexes distribution - LUL	☐ Bicep<br>☐ Supinator<br>☐ Tricep
Peripheral/lower motor neuron signs reduced/absent reflexes distribution - RLL	☐ Knee<br>☐ Ankle

Peripheral/lower motor neuron signs reduced/absent reflexes distribution - LLL	☐ Knee<br>☐ Ankle
Peripheral signs - age at onset - years	____________________<br>(If present at birth, enter 0 as value)
Peripheral signs - age at onset - months	____________________<br>(If present at birth, enter 0 as value)
Pyramidal signs	◯ No<br>◯ Yes<br>◯ Unknown
Pyramidal signs - type	☐ Spasticity<br>☐ Hyper-reflexia
Pyramidal signs spasticity - distribution	☐ RUL<br>☐ RLL<br>☐ LUL<br>☐ LLL<br>☐ Clonus
Pyramidal signs hyper-reflexia - distribution	☐ RUL<br>☐ LUL<br>☐ RLL<br>☐ LLL
Pyramidal signs hyper-reflexia distribution - RUL	☐ Bicep<br>☐ Supinator<br>☐ Tricep<br>☐ Finger flexors<br>☐ Hoffman's response
Pyramidal signs hyper-reflexia distribution - LUL	☐ Bicep<br>☐ Supinator<br>☐ Tricep<br>☐ Finger flexors<br>☐ Hoffman's response
Pyramidal signs hyper-reflexia distribution - RLL	☐ Knee<br>☐ Ankle<br>☐ Babinski
Pyramidal signs hyper-reflexia distribution - LLL	☐ Knee<br>☐ Ankle<br>☐ Babinski
Pyramidal signs - age at onset - years	____________________<br>(If present at birth, enter 0 as value)
Pyramidal signs - age at onset - months	____________________<br>(If present at birth, enter 0 as value)

Extrapyramidal signs	◯ No<br>◯ Yes<br>◯ Unknown
Extrapyramidal signs - type	☐ Rigidity<br>☐ Bradykinesia<br>☐ Tremor<br>☐ Loss of postural reflexes
Extrapyramidal signs - age of onset - years	______________________________<br>(If present at birth, enter 0 as value)
Extrapyramidal signs - age of onset - months	______________________________<br>(If present at birth, enter 0 as value)
Cerebellar signs	◯ No<br>◯ Yes<br>◯ Unknown
Cerebellar signs - type	☐ UL<br>☐ LL<br>☐ Gait<br>☐ Nystagmus<br>☐ Saccades<br>☐ Speech<br>☐ Tremor<br>☐ Trunk
Cerebellar signs - age at onset - years	______________________________<br>(If present at birth, enter 0 as value)
Cerebellar signs - age at onset - months	______________________________<br>(If present at birth, enter 0 as value)
Sensory loss	◯ Yes<br>◯ No<br>◯ Unknown
Sensory loss - age at onset - years	______________________________<br>(If present at birth, enter 0 as value)
Sensory loss - age at onset - months	______________________________<br>(If present at birth, enter 0 as value)
Pin prick	◯ Normal<br>◯ Abnormal<br>◯ Unknown
Pin prick - level and distribution	______________________________

Temperature	◯ Normal<br>◯ Abnormal<br>◯ Unknown
Temperature - level and distribution	__________________________
Vibration	◯ Normal<br>◯ Abnormal<br>◯ Unknown
Vibration - level and distribution	__________________________
Joint position sense	◯ Normal<br>◯ Abnormal<br>◯ Unknown
Joint position sense - level and distribution	__________________________
Joint position sense - pseudoathetosis	◯ No<br>◯ Yes<br>◯ Unknown

Hammersmith scale test

If the diagnosis is SMA Type 2 or 3, is a Hammersmith Scale result available?	◯ No<br>◯ Yes<br>◯ Unknown
Which Hammersmith Scale test was used? The Revised Hammersmith Scale (RHS) is preferred.	◯ Hammersmith Functional Motor Scale (HFMS)<br>◯ Modified Hammersmith Functional Motor Scale (MHFMS)<br>◯ Hammersmith Functional Motor Scale Expanded (HFMSE<br>◯ Revised Hammersmith Scale (RHS)<br>◯ Modified Hammersmith Functional Motor Scale Extended (MHFMS-Extend)<br>(http://www.smareachuk.org/smaoutcomemeasures/the-hammersmith-scale-what-is-it)
Hammersmith Scale test score	__________________________
Upload/view Hammersmith Scale test form	

North Star Ambulatory Assessment (NSAA)

If the diagnosis is DMD and participant is ambulatory and < 18 Years, is North Star Ambulatory Assessment (NSAA) result available?	◯ No<br>◯ Yes<br>◯ Unknown
Total NSAA score	__________________________<br>(0-34)
Upload/view NSAA test form	

Medical Research Council (MRC) sum score - used with the permission of the Medical Research Council

The total MRC sum score ranges from 0 (total paralysis) to 70 (normal strength). The score is the sum of the MRC score of 7 muscles (4 in the upper limbs and 3 in the lower limbs) on both sides, each muscle graded from 0 to 5.

Grade 5: Normal Strength
Grade 4: Ability to resist against moderate pressure throughout range of motion
Grade 3: Ability to move through full range of motion against gravity. If a subject has a contracture that limits joint movement, the mechanical range will be to the point at which the contracture causes joint restriction
Grade 2: Ability to move through full range of motion with gravity eliminated
Grade 1: A flicker of motion is seen or felt in the muscle
Grade 0: No movement

Upper limbs

	Grade 5	Grade 4	Grade 3	Grade 2	Grade 1	Grade 0
Right shoulder abduction (deltoid)	○	○	○	○	○	○
Right elbow flexion (biceps)	○	○	○	○	○	○
Right wrist extension (wrist extensors)	○	○	○	○	○	○
Right 1st DIO	○	○	○	○	○	○
Left shoulder abduction (deltoid)	○	○	○	○	○	○
Left elbow flexion (biceps)	○	○	○	○	○	○
Left wrist extension (wrist extensors)	○	○	○	○	○	○
Left 1st DIO	○	○	○	○	○	○

Lower limbs

	Grade 5	Grade 4	Grade 3	Grade 2	Grade 1	Grade 0
Right hip flexion (Ileopsoas)	○	○	○	○	○	○
Right knee extension (quadriceps femoris)	○	○	○	○	○	○
Right dorsiflexion foot (tibialis anterior)	○	○	○	○	○	○
Left hip flexion (Ileopsoas)	○	○	○	○	○	○
Left knee extension (quadriceps femoris)	○	○	○	○	○	○
Left dorsiflexion foot (tibialis anterior)	○	○	○	○	○	○

Total MRC sum score

____

CMTNS

Patient name: [subj_first_name] [subj_surname]

Date:

____
(YYYY-MM-DD)

Evaluator:

____

Sensory symptoms1
- ◯ None
- ◯ Symptoms below or at ankle bones
- ◯ Symptoms up to the distal half of the calf
- ◯ Symptoms up to the proximal half of the calf, including knee
- ◯ Symptoms above knee (above the top of the patella)

Motor symptoms legs2
- ◯ None
- ◯ Trips, catches toes, slaps feet. Shoe inserts
- ◯ Ankle support or stabilization (AFOs). Foot surgery5
- ◯ Walking aids (cane, walker)
- ◯ Wheelchair

Motor symptoms arms
- ◯ None
- ◯ Mild difficulty with buttons
- ◯ Severe difficulty or unable to do buttons
- ◯ Unable to cut most foods
- ◯ Proximal weakness (affect movements involving the elbow and above)

Pinprick sensibility1,3
- ◯ Normal
- ◯ Decreased below or at ankle bones
- ◯ Decreased up to the distal half of the calf
- ◯ Decreased up to the proximal half of the calf, including knee
- ◯ Decreased above knee (above the top of the patella)

Vibration4
- ◯ Normal
- ◯ Reduced at great toe
- ◯ Reduced at ankle
- ◯ Reduced at knee (tibial tuberosity)
- ◯ Absent at knee and ankle

Strength legs
- ◯ Normal
- ◯ 4+,4 or 4- on foot dorsiflexion or plantar flexion
- ◯ < 3 on foot dorsiflexion or < 3 on foot plantar flexion
- ◯ < 3 on foot dorsi and < 3 on plantar flexion
- ◯ Proximal weakness

Strength arms
- ◯ Normal
- ◯ 4+,4 or 4- on intrinsic hand muscles5
- ◯ < 3 on intrinsic hand muscles6
- ◯ < 5 on wrist extensors
- ◯ Weak above elbow

Ulnar CMAP (Median)	◯ >6mV (>4mV)<br>◯ 4-5.9mV (2.8-3.9)<br>◯ 2-3.9 mV (1.2-2.7)<br>◯ 0.1-1.9 mV (0.1-1.1)<br>◯ Absent (Absent)
Radial SAP amplitude, antidromic	◯ >15 ?V<br>◯ 10 - 14.9 ?V<br>◯ 5 - 9.9 ?V<br>◯ 1 - 4.9 ?V<br>◯ < 1 ?V

Notes: 1: Use the picture below to discriminate the level of the symptoms; 2: Uses aid most of the time. The patient was prescribed to wear/use or should be wearing/using the aid in the examiners opinion (see written instructions); 3: Abnormal if patient says it is definitely decreased compared to a normal reference point; 4: Use Rydell Seiffer tuning fork. Definition of Normal: > 5; 5: See written instructions for details of eligible foot surgery; 6: Intrinsic hand muscles strength assessment: Test only Abductor Pollicis Brevis (ABP) and First Dorsal Interosseus (FDI), then choose the stronger to give the score.

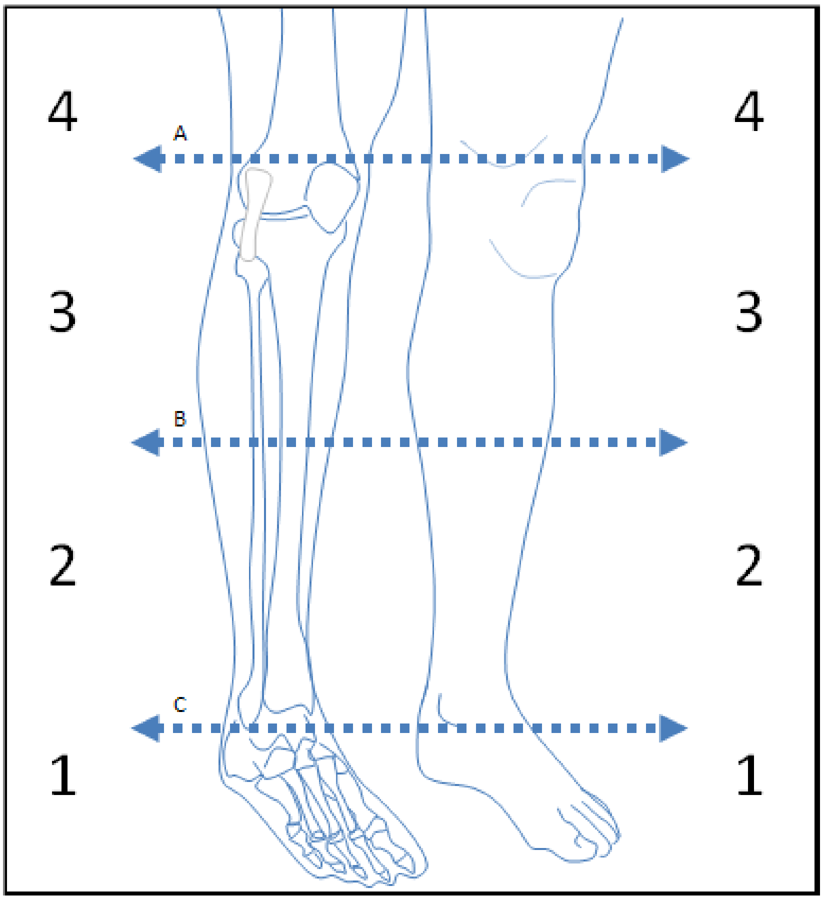

CMTSS Subtotal	________________________
CMTES Subtotal	________________________

CMTNS result

CMTNS Total

____

Patient details

Patient name: [subj_first_name] [subj_surname]

Age: [subj_age_yrs]

Gender: [subj_sex]

Raw scores

1. Functional Dexterity

______________________ (seconds)

2. 9 Hole Peg

______________________ (seconds)

3. Grip

______________________ (Newtons)

4. Plantarflexion

______________________ (Newtons)

5. Dorsiflexion

______________________ (Newtons)

6. Pinprick

- ◯ Normal
- ◯ Decreased below or at ankle bones
- ◯ Decreased at or below midline of calf
- ◯ Decreased above calf midline up to and including knee
- ◯ Decreased above knee (above top of patella)

7. Vibration

- ◯ Normal
- ◯ Reduced at first metatarsal bone
- ◯ Reduced at ankle
- ◯ Reduced at knee (tibial tuberosity)
- ◯ Absent at knee and ankle

8. Balance

______________________ (Bruininks Oseretsky (0-37))

9. Gait

Foot drop

- ◯ No
- ◯ Some
- ◯ Yes

Difficulty heel walking	◯ No<br>◯ Some<br>◯ Yes
Difficulty toe walking	◯ No<br>◯ Some<br>◯ Yes
10. Long Jump	__________________________________<br>(centimetres)
11. 6 Minute Walk	__________________________________<br>(metres)

CMTPedS result

Total CMTPedS Score	__________________________________<br>(CMTPedS calculator: https://www.cmtpeds.org/)

Report

Participant CMTPedS report	(Upload/view CMTPedS report)

Comments

Any other comments on clinical assessment	____

Follow-up comments

Comments from follow-up visits	____<br>(For utility; not expected as part of the ICGNMD study (cross-sectional))

Investigations

EMG

EMG	◯ Normal<br>◯ Abnormal<br>◯ Not done
EMG - abnormality	☐ Myopathic<br>☐ Neurogenic<br>☐ Neuromuscular transmission disorder<br>☐ Other
EMG abnormality - other	______________________
EMG spontaneous activity	◯ No<br>◯ Yes<br>◯ Not done
EMG spontaneous activity - type	◯ Myotonic/pseudomyotonic<br>◯ Fibrillation<br>◯ Positive sharp waves<br>◯ Fasciculations
EMG - report	(Upload/view EMG report)
EMG images available	◯ No<br>◯ Yes<br>◯ Unknown

Nerve conduction studies

Motor nerve conduction studies	◯ Normal<br>◯ Abnormal<br>◯ Not done
Motor nerve conduction studies - abnormality	◯ Axonal<br>◯ Demyelinating<br>◯ Mixed
Sensory nerve conduction studies	◯ Normal<br>◯ Abnormal<br>◯ Not done
Sensory nerve conduction studies - abnormality	◯ Axonal<br>◯ Demyelinating<br>◯ Mixed
Nerve conduction studies - report	(Upload/view Nerve conduction study report)

Echocardiogram

Echocardiogram	◯ Normal<br>◯ Abnormal<br>◯ Not done
Echocardiogram - report	(Upload/view Echocardiogram report)

MRI

Brain MRI	◯ Normal<br>◯ Abnormal<br>◯ Not done
Brain MRI - findings	☐ White matter abnormalities<br>☐ Lissencephaly<br>☐ Polymicrogyria<br>☐ Brainstem abnormalities<br>☐ Cerebral atrophy<br>☐ Basal ganglia atrophy<br>☐ Cerebellar atrophy<br>☐ Cerebellar dysplasia<br>☐ Cerebellar cysts<br>☐ Optic nerve abnormalities<br>☐ Other
Brain MRI - findings - other	______________________________
Brain MRI - report	(Upload/view Brain MRI report)
Spine MRI	◯ Normal<br>◯ Abnormal<br>◯ Not done
Spine MRI - report	(Upload/view Spine MRI report)
Limbs MRI	◯ Normal<br>◯ Abnormal<br>◯ Not done
Limbs MRI - report	(Upload/view Limbs MRI report)
MRI images available	◯ No<br>◯ Yes<br>◯ Unknown

Muscle ultrasound

Muscle ultrasound	◯ Normal<br>◯ Abnormal<br>◯ Not done
Muscle ultrasound - report	(Upload/view Muscle ultrasound report)

Nerve ultrasound

Nerve ultrasound	◯ Normal<br>◯ Abnormal<br>◯ Not done
Nerve ultrasound - report	(Upload/view Nerve ultrasound report)

Muscle biopsy

Muscle biopsy	◯ Normal<br>◯ Abnormal<br>◯ Not done
Suggestive of muscular dystrophy	◯ No<br>◯ Yes<br>◯ Unknown<br>(Internalized nuclei, fibrosis, fibre size variation, rounded fibers.)
Muscular dystrophy - findings	☐ Alpha dystroglycanopathy<br>☐ Merosin (laminin 2) deficiency<br>☐ Collagen VI related myopathy<br>☐ Myofibrillar myopathy (desmin, myotilin, VCP abnormal staining)<br>☐ Caveolin deficiency<br>☐ Emerin deficiency<br>☐ Calpain deficiency<br>☐ Dystrophin deficiency<br>☐ Sarcoglycan deficiency<br>☐ Dysferlin deficiency<br>☐ Other
Muscular dystrophy - merosin deficiency	◯ Partial<br>◯ Complete<br>◯ Unknown
Muscular dystrophy findings - other	______________________________
Suggestive of congenital myopathy	◯ No<br>◯ Yes<br>◯ Unknown

Congenital myopathy - findings	☐ Central core disease<br>☐ Centronuclear/Myotubular myopathy<br>☐ Congenital fibre type disproportion<br>☐ Multiminicore myopathy<br>☐ Nemaline myopathy<br>☐ Other
Congenital myopathy findings - other	__________________________________
Suggestive of mitochondrial disease	◯ No<br>◯ Yes<br>◯ Unknown
Mitochondrial disease - findings	☐ Ragged red fibres<br>☐ COX negative fibres<br>☐ COX/SDH positive fibres<br>☐ Other
Mitochondrial disease findings - other	__________________________________
Mitochondrial disease - respiratory chain enzyme analysis	◯ No<br>◯ Yes<br>◯ Unknown
Mitochondrial disease respiratory chain enzyme analysis - findings	__________________________________
Mitochondrial disease - respiratory chain enzyme analysis - report	(Upload/view Respiratory chain enzyme analysis report)
Additional histological findings	☐ Inflammatory infiltrates<br>☐ Myofibrillar changes<br>☐ Mitochondrial changes<br>☐ Rimmed vacuoles<br>☐ Protein aggregates<br>☐ Neurogenic change
Muscle biopsy - report	(Upload/view Muscle biopsy report)
Muscle biopsy slides available	◯ No<br>◯ Yes<br>◯ Unknown

Nerve biopsy

Nerve biopsy	◯ Normal<br>◯ Abnormal<br>◯ Not done
Nerve biopsy - report	(Upload/view Nerve biopsy report)
Nerve biopsy slides available	◯ No<br>◯ Yes<br>◯ Unknown

Other tissue studies

Other tissue studies	◯ No<br>◯ Yes<br>◯ Unknown
Other tissue studies done	__________________________________
Other tissue studies - report	(Upload/view Other tissue studies report)

Creatine phosphokinase (CPK)

CPK tested	◯ No<br>◯ Yes<br>◯ Unknown
CPK result - 1	__________________________________
CPK unit - 1	__________________________________
CPK tested - age - years - 1	__________________________________
CPK tested - age - months - 1	__________________________________
CPK result - 2	__________________________________
CPK unit - 2	__________________________________
CPK tested - age - years - 2	__________________________________
CPK tested - age - months - 2	__________________________________

CPK result - 3

____

CPK unit - 3

____

CPK tested - age - years - 3

____

CPK tested - age - months - 3

____

Additional laboratory tests

Additional laboratory tests available

◯ No
◯ Yes
◯ Unknown

Additional laboratory tests available - type

☐ Alb
☐ ALP
☐ ALT
☐ Ammonia (plasma)
☐ Blood lactate
☐ Cr
☐ CrCl
☐ CSF lactate
☐ Fasting glucose level
☐ GGT
☐ Glu (random)
☐ Hb
☐ HbA1c
☐ K
☐ MCV
☐ Na
☐ Plt
☐ T4
☐ TSH
☐ Urea
☐ WCC
☐ Other

Alb

____

Alb - unit

____

ALP

____

ALP - unit

____

ALT

____

ALT - unit

____

Ammonia (plasma)	________________
Ammonia (plasma) - unit	________________
Blood lactate	________________
Blood lactate - unit	________________
Cr	________________
Cr - unit	________________
CrCl	________________
CrCl - unit	________________
CSF lactate	________________
CSF lactate - unit	________________
Fasting glucose level	________________
Fasting glucose level - unit	________________
GGT	________________
GGT - unit	________________
Glu (random)	________________
Glu (random) - unit	________________
Hb	________________
Hb - unit	________________

HbA1c	______________________
HbA1c - unit	______________________
K	______________________
K - unit	______________________
MCV	______________________
MCV - unit	______________________
Na	______________________
Na - unit	______________________
Plt	______________________
Plt - unit	______________________
T4	______________________
T4 - unit	______________________
TSH	______________________
TSH - unit	______________________
Urea	______________________
Urea - unit	______________________
WCC	______________________
WCC - unit	______________________

Lab test - other(s)

____

HIV and hepatitis status

HIV status

◯ Negative
◯ Positive
◯ Unknown

HIV status - CD4 count

____

HIV status - viral load

____

Hep A status

◯ Negative
◯ Positive
◯ Unknown

Hep B status

◯ Negative
◯ Positive
◯ Unknown

Hep C status

◯ Negative
◯ Positive
◯ Unknown

Other known infectious diseases

List any other known infectious diseases

____

Comments

Any other comments on investigations

____

Non Icgnmd Genetic Testing

Have any of the following genetic tests been performed? (or are they currently being performed?)

	No	Yes, abnormal result (confirms diagnosis)	Yes, normal result	In progress	Unknown
SMA (SMN1) MLPA	○	○	○	○	○
DMD MLPA	○	○	○	○	○
DMD Sequencing	○	○	○	○	○
CMT1A MLPA (17p duplication analysis)	○	○	○	○	○
Myotonic dystrophy Type 1 testing	○	○	○	○	○
Myotonic dystrophy Type 2 testing	○	○	○	○	○
FSHD testing	○	○	○	○	○
Familial ALS (C9ORF72)	○	○	○	○	○
RYR-1 Screening for South African founder mutations	○	○	○	○	○
CANVAS	○	○	○	○	○

Please give details of result(s) ______________________________

Optional: Pseudonymised reports can be uploaded here

(Zip if more than one)

Record details of other local testing and local testing limitations here ______________________________

You can upload de-identified copies of non-ICGNMD genetic reports here (.pdf, .jpg, .png)

Are any of the following diagnostic tests required, but not available locally, for this participant?

- ☐ SMA (SMN1) MLPA
- ☐ DMD MLPA
- ☐ DMD Sequencing
- ☐ CMT1A MLPA (17p duplication analysis)
- ☐ Myotonic dystrophy Type 1 testing
- ☐ Myotonic dystrophy Type 2 testing
- ☐ FSHD testing
- ☐ Familial ALS (C9ORF72)
- ☐ RYR-1 Screening for South African founder mutations
- ☐ CANVAS

Has mitochondrial DNA testing been performed?

	No	Yes, abnormal result (confirms diagnosis)	Yes, normal result	In progress	Unknown
Common mitochondrial point mutations	○	○	○	○	○
Whole mitochondrial genome sequencing	○	○	○	○	○
mtDNA rearrangement analysis	○	○	○	○	○
mtDNA depletion analysis	○	○	○	○	○
Other	○	○	○	○	○

Other - please specify test ______________________________

Please give details of result(s) ______________________________

Has any exome sequencing been performed (targeted exome/ gene panel, or whole exome sequencing)?

	No	Yes	In progress	Unknown
Whole Exome Sequencing as singleton	○	○	○	○
Whole Exome Sequencing duo	○	○	○	○
Whole Exome Sequencing trio or larger	○	○	○	○
Gene Panel	○	○	○	○
Targeted Exome	○	○	○	○

Please give details e.g. positive results or family members included. Panel content may also be listed here. ______________________________

Are any other diagnostic genetic tests outstanding?
- ○ No
- ○ Yes
- ○ Unknown

Please give details ______________________________

Personal Details

Personal details	
Clinic/lab/local study subject ID	______________________ (Primary identifier at host)
Initials	______________________
Year of birth	______________________

Sample Tracking

SENDER TO COMPLETE	
Samples shipped for genetic analysis	◯ No<br>◯ Yes

Sample type(s) shipped	
DNA	◯ No<br>◯ Yes
Number of DNA sample tubes sent	◯ 1<br>◯ 2<br>◯ 3<br>◯ 4
Blood	◯ No<br>◯ Yes
Number of blood sample tubes sent	◯ 1<br>◯ 2<br>◯ 3<br>◯ 4
Skin tissue/fibroblast	◯ No<br>◯ Yes
Number of skin tissue sample tubes sent	◯ 1<br>◯ 2<br>◯ 3<br>◯ 4
Muscle tissue	◯ No<br>◯ Yes
Number of muscle tissue sample tubes sent	◯ 1<br>◯ 2<br>◯ 3<br>◯ 4
Buccal	◯ No<br>◯ Yes
Number of buccal sample tubes sent	◯ 1<br>◯ 2<br>◯ 3<br>◯ 4
Saliva	◯ No<br>◯ Yes

Number of saliva sample tubes sent	◯ 1<br>◯ 2<br>◯ 3<br>◯ 4
Urine	◯ No<br>◯ Yes
Number of urine sample tubes sent	◯ 1<br>◯ 2<br>◯ 3<br>◯ 4
Date samples sent	____________________<br>(YYYY-MM-DD)
Courier tracking ID	____________________
Upload/view photo of sample	(Photo of 1 x sample tube showing ICGNMD Study ID on tube to be shipped)
Upload/view sample manifest	
Sender comments	________________________

RECIPIENT TO COMPLETE

UCL OR IN-COUNTRY GENETIC ANALYSIS PROVIDER (TURKEY, INDIA)

Sample received	◯ No<br>◯ Yes
Date samples received	____________________<br>(YYYY-MM-DD)
Samples received ok?	◯ No<br>◯ Yes
Issue with samples received	◯ Sample damage/loss<br>◯ Sample ID unclear<br>◯ Sample absent from box<br>◯ Other
Issue with samples received - other	____________________
Sample quality	◯ Acceptable<br>◯ Re-send existing sample<br>◯ Collect fresh sample

Upload/view photo of received sample	(Photo of 1 x sample tube showing ICGNMD Study ID on tube to be shipped)
Date samples processed	______________________ (YYYY-MM-DD)
Processed sample information	
Recipient comments	______________________

RETURNS

Will some or all of the processed samples be returned to sender?	◯ No<br>◯ Yes
Date samples returned to sender	______________________ (YYYY-MM-DD)
Sample return comments	______________________ (Person shipping can add notes here)
Sample return complete comments	______________________ (Person at destination can add notes here)

THIRD PARTY PROCESSING

Will some or all of the processed samples be shipped to third party?	◯ No<br>◯ Yes
Date samples sent to third Party	______________________ (YYYY-MM-DD)
Third party processing comments	______________________ (Person shipping can add notes for third party recipient here)

Cohort Management

**Participant update**	
Is the participant dead?	◯ Yes
Age at death - years	______________________
Age at death - months	______________________
Cause of death - type	◯ NMD-disease related<br>◯ Non-NMD-disease related
Cause of death - description	______________________
Full consent for entry/continued data use post-mortem by the participant or parent/guardian?	◯ No<br>◯ Yes<br>◯ Undetermined
Any other comments on participant	____________________________

Monitoring And Analysis

RELATIVE: ICGNMD genetic testing

Relative affected or unaffected?

◯ Affected
◯ Unaffected

Relative Test #1

ICGNMD Test #1 done on this relative's sample

◯ Reanalyse non-ICGNMD WES/WGS data
◯ Single gene test
◯ Sanger
◯ Array
◯ WES
◯ WGS
(Always record relative's results separate from proband results)

Date of test

____
(YYYY-MM-DD)

Summary of this relative's test result

____

Upload this relative's report here

Optional: upload additional ICGNMD report for this relative

Add another ICGNMD test result for this relative?

◯ yes
◯ no

Relative Test #2

ICGNMD Test #2 done on this relative's sample

◯ Single gene test
◯ Sanger
◯ Array
◯ WES
◯ WGS
(Always record relative's results separate from proband results)

Date of test #2

____
(YYYY-MM-DD)

Summary of relative's test result

____

Upload relative report here

Optional: upload additional ICGNMD report for this relative	
Add another ICGNMD test result for this relative?	◯ yes<br>◯ no

Relative Test #2

ICGNMD Test #3 done on this relative's sample	◯ Single gene test<br>◯ Sanger<br>◯ Array<br>◯ WES<br>◯ WGS<br>(Always record relative's results separate from proband results)
Date of test #3	______________________<br>(YYYY-MM-DD)
Summary of relative's test result	____________________________
Upload relative report here	
Optional: upload additional ICGNMD report for this relative	

UNRELATED CONTROL PARTICIPANT: ICGNMD Genetic testing

ICGNMD Test done on this unrelated control sample	◯ Single gene test<br>◯ Sanger<br>◯ Array<br>◯ WES<br>◯ WGS
Date of test	______________________<br>(YYYY-MM-DD)
Any notes for control test data	____________________________
Upload unrelated control report here	

Genetic Test Planning

Test Planning Note #1	______________________
Date of Discussion	______________________ (YYYY-MM-DD)
Add another discussion note?	◯ Yes
Test Planning Note #2	______________________
Date of Discussion	______________________ (YYYY-MM-DD)
Add another discussion note?	◯ Yes
Test Planning Note #3	______________________
Date of Discussion	______________________ (YYYY-MM-DD)
Add another discussion note?	◯ Yes
Test Planning Note #4	______________________ (If >4 test discussions, add here)
Date of Discussion	______________________ (YYYY-MM-DD)
Participant already genetically "solved", no ICGNMD test needed?	◯ yes<br>◯ yes but want to discuss local results with ICGNMD<br>◯ no<br>(No ICGNMD report generated if solved locally)

Local tests/checks needed OUTSIDE ICGNMD project (Not funded by ICGNMD)

Any local tests/checks to do outside ICGNMD Project?	◯ yes<br>◯ no<br>(May be BEFORE or AT SAME TIME as ICGNMD testing. List any tests NOT FUNDED by ICGNMD award.)
Local tests/checks to do BEFORE ICGNMD test can start	◯ yes<br>◯ no

Summary of local tests/checks to do BEFORE ICGNMD testing

____
(Most recent notes at top, with date & initials)

Local tests/checks completed now? (Local team to complete)

- ◯ Yes, local tests complete: start ICGNMD test
- ◯ Yes, local tests complete: review & decide next steps
- ◯ Yes, local tests complete: ICGNMD test NO LONGER NEEDED
- ◯ No, local work in progress: ICGNMD testing can't start yet

(REMEMBER! Update "Non-ICGNMD Genetic Testing" section with local test results)

Local tests/checks to do AT SAME TIME as ICGNMD testing

- ◯ yes
- ◯ no

Summary of local tests/checks to do AT SAME TIME AS ICGNMD testing

____
(Most recent notes at top, with date & initials)

Local checks/test results - summary by local team

____
(REMEMBER! Update other REDCap sections with local test results)

ICGNMD reanalyse Non-ICGNMD data

Reanalyse existing WES/WGS data with ICGNMD pipeline?

- ◯ Yes
- ◯ No

Date of Joint Decision

____
(YYYY-MM-DD)

Reason for re-analysis of non-ICGNMD data

____

File format

- ◯ fastq (Best format)
- ◯ vcf (OK)
- ◯ Other (Check first)

File name (must contain ICGNMD Study ID)

____
(Transferring site must complete)

File(s) transferred to UCL?

- ◯ Yes
- ◯ No

(Transferring site must complete)

Date file transferred to UCL

____
(YYYY-MM-DD)

PanelApp filters

Select 1-5 PanelApp Panels to filter PDF report?	◯ Yes<br>◯ Not yet<br>(SELECT 1 to 5 PANELS)
Channelopathies	☐ Brain channelopathy<br>☐ Skeletal muscle channelopathy
Inherited Epilepsy Syndromes	☐ Genetic epilepsy syndromes
Mitochondrial	☐ Mitochondrial Disorders
Motor and Sensory Disorders of the PNS	☐ Hereditary neuropathy<br>☐ Paediatric motor neuronopathies
Motor Disorders of the CNS	☐ Cerebellar hypoplasia<br>☐ Early onset dystonia<br>☐ Hereditary spastic paraplegia<br>☐ Neurotransmitter disorders<br>☐ Structural basal ganglia disorders
Neurodegenerative Disorders	☐ Amyotrophic lateral sclerosis/motor neuron disease<br>☐ Hereditary ataxia - adult onset
Neurodevelopmental disorders	☐ Intellectual disability<br>☐ Malformations of cortical development<br>☐ Hereditary ataxia and cerebellar anomalies - childhood onset
Neuromuscular disorders	☐ Arthrogryposis<br>☐ Congenital muscular dystrophy<br>☐ Congenital myaesthenic syndrome<br>☐ Congenital myopathy<br>☐ Distal myopathies<br>☐ Limb girdle muscular dystrophy<br>☐ Rhabdomyolysis and metabolic muscle disorders
Parenchymal brain disorders	☐ Intracerebral calcification disorders
Peroxisomal disorders	☐ Peroxisomal disorders
Connective tissues disorders	☐ Ehlers Danlos syndromes
Specific metabolic abnormalities	☐ Undiagnosed metabolic disorders
List other PanelApp panels here	____________________<br>(Use EXACT panel names as listed at https://panelapp.genomicsengland.co.uk/panels/)
Additional analysis notes, e.g. specific genes to check	____________________<br>(Note: Checking specific genes NOT part of standard pipeline)

Request bespoke ICGNMD analysis?

Select bespoke ICGNMD analyses (in addition to Panels)
- ◯ Yes
- ◯ Not yet

(Please only select if essential: bespoke analyses use a lot of ICGNMD time.)

Bespoke ICGNMD analyses
- ☐ mtDNA analysis
- ☐ CNV or SV detection
- ☐ notinuse1
- ☐ notinuse2

(Please only select if essential: bespoke analyses use a lot of ICGNMD time.)

ICGNMD Report for Reanalysed Non-ICGNMD Data

UCL research report uploaded

____
(YYYY-MM-DD)

ICGNMD research report (summary PDF with filters)

ICGNMD .csv file (no panel filters)

(If requested) ICGNMD bespoke analysis report

ICGNMD Review of Reanalysis

Date of joint review of result

____
(YYYY-MM-DD)

Has ICGNMD reanalysis provisionally solved?
- ◯ Yes: provisionally solved
- ◯ Yes, confirms other test result
- ◯ No, has not supported a research finding

Causative variant #1

Condition #1 [Orphanet]

____

Free text box if Orphanet not suitable

____

Caustive gene & variant #1 [OMIM]

____

Free text box if OMIM not suitable

____

Known gene mutation 1 - VCF

____
(Use format (CHR POS ID REF ALT): 1 230710048 rs699 A G)

Variant results #1 - freetext notes

____

Variant #1 Provisional classification by ICGNMD/ACMG	◯ Pathogenic<br>◯ Likely Pathogenic<br>◯ VUS - Suspected Pathogenic<br>◯ VUS
Add another variant?	◯ yes<br>◯ no

Causative variant #2

Condition #2 [Orphanet]	__________________________________
Free text box if Orphanet not suitable	__________________________________
Caustive gene & variant #2 [OMIM]	__________________________________
Free text box if OMIM not suitable	__________________________________
Known gene mutation 2 - VCF	__________________________________<br>(Use format (CHR POS ID REF ALT): 1 230710048 rs699 A G)
Variant #2 Provisional classification by ICGNMD/ACMG	◯ Pathogenic<br>◯ Likely Pathogenic<br>◯ VUS - Suspected Pathogenic<br>◯ VUS
Variant #2 results - freetext notes	__________________________________
Add another variant?	◯ yes<br>◯ no

Causative variant #3

Condition #3 [Orphanet]	__________________________________
Free text box if Orphanet not suitable	__________________________________
Caustive gene & variant #3 [OMIM]	__________________________________
Free text box if OMIM not suitable	__________________________________
Known gene mutation 3 - VCF	__________________________________<br>(Use format (CHR POS ID REF ALT): 1 230710048 rs699 A G)

Variant #3 Provisional classification by ICGNMD/ACMG	◯ Pathogenic<br>◯ Likely Pathogenic<br>◯ VUS - Suspected Pathogenic<br>◯ VUS
Variant #3 results - freetext notes	______________________
Add another variant?	◯ yes<br>◯ no

Causative variant #4

Condition #4 [Orphanet]	______________________
Free text box if Orphanet not suitable	______________________
Caustive gene & variant #4 [OMIM]	______________________
Free text box if OMIM not suitable	______________________
Known gene mutation 4 - VCF	______________________<br>(Use format (CHR POS ID REF ALT): 1 230710048 rs699 A G)
Variant #4 Provisional classification by ICGNMD/ACMG	◯ Pathogenic<br>◯ Likely Pathogenic<br>◯ VUS - Suspected Pathogenic<br>◯ VUS
Variant #4 results - freetext notes	______________________

ICGNMD Next steps

ICGNMD testing needed after reanalysis?	◯ Yes<br>◯ No
Which additional test?	◯ WES<br>◯ WGS<br>◯ Sanger seq<br>◯ Array<br>◯ Single Gene Test<br>◯ Reanalyse WES data<br>(If you select a new test, complete that section)

ICGNMD Single Gene Test #1

Go to ICGNMD single gene test #1?

◯ Yes
◯ No

Date of Joint Decision

____
(YYYY-MM-DD)

Notes (e.g. justification, test conditions)

____

Which country will test take place in?

◯ UK (or Leiden for some FSHD)
◯ India
◯ Brazil
◯ Other

Single Gene Test #1 Agreed

At UCL or Leiden

◯ DM1
◯ CMT1a MLPA
◯ c9orf72
◯ SMA MLPA
◯ FSHD1
◯ FSHD2
◯ OPMD
◯ CANVAS
◯ Friedreich's Ataxia
◯ SCAS
◯ Other

Other test or extra detail

____

In India

◯ DM1
◯ CMT1a MLPA
◯ c9orf72
◯ SMA MLPA
◯ FSHD1
◯ FSHD2
◯ OPMD
◯ CANVAS
◯ Friedreich's Ataxia
◯ SCAS
◯ DMD MLPA
◯ DMD gene seq
◯ Other

Other test or extra detail

____

In Brazil

◯ pmp22
◯ MPZ
◯ GJB1
◯ Other

Other test or extra detail

____

Other (enter single gene test & country)

____

Same single gene test for relatives AT SAME TIME as proband?

Test relatives as well?
- ◯ Yes
- ◯ No

Optional: Relative #1

Relative #1: Relation to proband
- ◯ mother
- ◯ father
- ◯ brother
- ◯ sister
- ◯ other

Is this Relative also affected?
- ◯ yes
- ◯ no

Relative #1 Study ID (IC_ABC_NNNNN)

____

Add another relative?
- ◯ yes
- ◯ no

Optional: Relative #2

Relative #2: Relation to proband
- ◯ mother
- ◯ father
- ◯ brother
- ◯ sister
- ◯ other

Is this Relative also affected?
- ◯ yes
- ◯ no

Relative #2 Study ID (IC_ABC_NNNNN)

____

Add another relative?
- ◯ yes
- ◯ no

Optional: Relative #3

Relative #3: Relation to proband	◯ mother<br>◯ father<br>◯ brother<br>◯ sister<br>◯ other
Is this Relative also affected?	◯ yes<br>◯ no
Relative #3 Study ID (IC_ABC_NNNNN)	__________________________________
Add another relative?	◯ yes<br>◯ no

Optional: Relative #4

Relative #4: Relation to proband	◯ mother<br>◯ father<br>◯ brother<br>◯ sister<br>◯ other
Is this Relative also affected?	◯ yes<br>◯ no
Relative #4 Study ID (IC_ABC_NNNNN)	__________________________________

Relatives: summary

Tick box when relative(s) single gene tests complete and their REDCap records updated?	☐ family single gene test 1 complete<br>(Record relative's results in own REDCap record - not in proband record)

Sample tracking

Samples available at test site (or test co-ordinating site)?	◯ yes<br>◯ no<br>◯ not all samples
Notes on sample availability at testing/test co-ordinating site	__________________________________
(Optional) Samples sent to approved 3rd party test lab?	◯ yes<br>◯ no<br>(MTA & Terms of Service must be in place prior to sending to 3rd party)
(Optional) Date samples sent to approved 3rd party	____________________________<br>(YYYY-MM-DD)

After-test reporting

Result Quality Satisfactory?	◯ yes<br>◯ no
Testing site to repeat test on same sample?	◯ yes<br>◯ no
New sample needed to repeat?	◯ yes<br>◯ no
Decision = do not repeat test	◯ yes<br>◯ no
Reason for single gene test failure?	__________________________________
Data file for failed test	(OPTIONAL: upload file to show failed test or low quality outputs)
Single Gene Test Results file (research only)	(Upload/View Single Gene Test report)
Single gene: test provisional results	◯ Supports diagnosis<br>◯ Partially supports diagnosis<br>◯ Does not support diagnosis<br>◯ Inconclusive result

UCL ICGNMD Report for Single Gene Test#1

UCL research report uploaded	____________________________<br>(YYYY-MM-DD)
ICGNMD research report	

ICGNMD Review of Single Gene Test #1

Date of joint review of result	____________________________<br>(YYYY-MM-DD)
Condition [Orphanet]	____________________________
Free text box if Orphanet not suitable	____________________________
Caustive gene & variant [OMIM]	____________________________
Free text box if OMIM not suitable	____________________________

Known gene mutation 1 - VCF

____
(Use format (CHR POS ID REF ALT): 1 230710048 rs699 A G)

Single gene test #1 results - freetext notes

____

Provisional classification by ICGNMD/ACMG

◯ Pathogenic
◯ Likely Pathogenic
◯ VUS - Suspected Pathogenic
◯ VUS

Has Single Gene Test #1 provisionally solved?

◯ Yes: provisionally solved with this test
◯ Yes, this test confirms other test result
◯ No, this test has not supported a research finding

ICGNMD Next steps

More ICGNMD testing needed after Single Gene Test #1?

◯ Yes
◯ No

Which additional test?

◯ Same test on relatives
◯ WES
◯ WGS
◯ Sanger seq
◯ Array
◯ Different Single Gene Test
(If you select a new test, complete that section)

Sample tracking

Relative samples at test site (or test co-ordinating site)?

◯ yes
◯ no
◯ not all samples

Notes on sample availability at testing/test co-ordinating site

____

Optional: Relative #1

Relative #1: Relation to proband

◯ mother
◯ father
◯ brother
◯ sister
◯ other

Is this Relative also affected?

◯ yes
◯ no

Relative #1 Study ID (IC_ABC_NNNNN)

____

Add another relative?

◯ yes
◯ no

Optional: Relative #2

Relative #2: Relation to proband	◯ mother<br>◯ father<br>◯ brother<br>◯ sister<br>◯ other
Is this Relative also affected?	◯ yes<br>◯ no
Relative #2 Study ID (IC_ABC_NNNNN)	__________________________________
Add another relative?	◯ yes<br>◯ no

Optional: Relative #3

Relative #3: Relation to proband	◯ mother<br>◯ father<br>◯ brother<br>◯ sister<br>◯ other
Is this Relative also affected?	◯ yes<br>◯ no
Relative #3 Study ID (IC_ABC_NNNNN)	__________________________________
Add another relative?	◯ yes<br>◯ no

Optional: Relative #4

Relative #4: Relation to proband	◯ mother<br>◯ father<br>◯ brother<br>◯ sister<br>◯ other
Is this Relative also affected?	◯ yes<br>◯ no
Relative #4 Study ID (IC_ABC_NNNNN)	__________________________________

Relatives: summary

Tick box when relative(s) single gene test1 family REDCap records updated?

☐ family single gene test 1 records completed (Record relative's results in own REDCap record - not in proband record)

ICGNMD Review of later relative tests

Date of joint review of additional family single gene tests

____
(YYYY-MM-DD)

(Proband record only) What has single gene testing of relatives added to proband's ICGNMD research findings?

____

ICGNMD Single Gene Test #2

Go to ICGNMD single gene test #2?

○ Yes
○ No

Date of Joint Decision

____
(YYYY-MM-DD)

Notes (e.g. justification, test conditions)

____

Which country will test take place in?

○ UK (or Leiden for some FSHD)
○ India
○ Brazil
○ Other

Single Gene Test #2 Agreed

At UCL or Leiden

○ DM1
○ CMT1a MLPA
○ c9orf72
○ SMA MLPA
○ FSHD1
○ FSHD2
○ OPMD
○ CANVAS
○ Friedreich's Ataxia
○ SCAS
○ Other

Other test or extra detail

____

In India	◯ DM1<br>◯ CMT1a MLPA<br>◯ c9orf72<br>◯ SMA MLPA<br>◯ FSHD1<br>◯ FSHD2<br>◯ OPMD<br>◯ CANVAS<br>◯ Friedreich's Ataxia<br>◯ SCAS<br>◯ DMD MLPA<br>◯ DMD gene seq<br>◯ Other
Other test or extra detail	__________________________________
In Brazil	◯ pmp22<br>◯ MPZ<br>◯ GJB1<br>◯ Other
Other test or extra detail	__________________________________
Other (enter single gene test & country)	__________________________________

Same single gene test for relatives AT SAME TIME as proband?

Test relatives as well?	◯ Yes<br>◯ No

Optional: Relative #1

Relative #1: Relation to proband	◯ mother<br>◯ father<br>◯ brother<br>◯ sister<br>◯ other
Is this Relative also affected?	◯ yes<br>◯ no
Relative #1 Study ID (IC_ABC_NNNNN)	__________________________________
Add another relative?	◯ yes<br>◯ no

Optional: Relative #2

Relative #2: Relation to proband	◯ mother<br>◯ father<br>◯ brother<br>◯ sister<br>◯ other
Is this Relative also affected?	◯ yes<br>◯ no
Relative #2 Study ID (IC_ABC_NNNNN)	__________________________________
Add another relative?	◯ yes<br>◯ no

Optional: Relative #3

Relative #3: Relation to proband	◯ mother<br>◯ father<br>◯ brother<br>◯ sister<br>◯ other
Is this Relative also affected?	◯ yes<br>◯ no
Relative #3 Study ID (IC_ABC_NNNNN)	__________________________________
Add another relative?	◯ yes<br>◯ no

Optional: Relative #4

Relative #4: Relation to proband	◯ mother<br>◯ father<br>◯ brother<br>◯ sister<br>◯ other
Is this Relative also affected?	◯ yes<br>◯ no
Relative #4 Study ID (IC_ABC_NNNNN)	__________________________________

Sample tracking

Samples available at test site (or test co-ordinating site)?	◯ yes<br>◯ no<br>◯ not all samples
Notes on sample availability at testing/test co-ordinating site	__________________

Relatives: summary

Tick box when relative(s) testing complete and their REDCap records updated	☐ family single gene test 2 complete<br>(Record relative's results in own REDCap record - not in proband record)
(Optional) Samples sent to approved 3rd party test lab?	◯ yes<br>◯ no<br>(MTA & Terms of Service must be in place prior to sending to 3rd party)
(Optional) Date samples sent to approved 3rd party	__________________<br>(YYYY-MM-DD)

After-test reporting

Result Quality Satisfactory?	◯ yes<br>◯ no
Testing site to repeat test on same sample?	◯ yes<br>◯ no
New sample needed to repeat?	◯ yes<br>◯ no
Decision = do not repeat test	◯ yes<br>◯ no
Reason for single gene test failure?	__________________
Data file for failed test	(OPTIONAL: upload file to show failed test or low quality outputs)
Single Gene Test Results file (research only)	(Upload/View Single Gene Test report)
Single gene: test provisional results	◯ Supports diagnosis<br>◯ Partially supports diagnosis<br>◯ Does not support diagnosis<br>◯ Inconclusive result

UCL ICGNMD Report for Single Gene Test#2

UCL research report uploaded

____
(YYYY-MM-DD)

ICGNMD research report

ICGNMD Review of Single Gene Test #2

Date of joint review of result

____
(YYYY-MM-DD)

Condition [Orphanet]

____

Free text box if Orphanet not suitable

____

Caustive gene & variant [OMIM]

____

Free text box if OMIM not suitable

____

Known gene mutation 1 - VCF

____
(Use format (CHR POS ID REF ALT): 1 230710048 rs699 A G)

Single gene test #2 results - freetext notes

____

Provisional classification by ICGNMD/ACMG

- ◯ Pathogenic
- ◯ Likely Pathogenic
- ◯ VUS - Suspected Pathogenic
- ◯ VUS

Has Single Gene Test #2 provisionally solved?

- ◯ Yes: provisionally solved with this test
- ◯ Yes, this test confirms other test result
- ◯ No, this test has not supported a research finding

ICGNMD Next steps

More ICGNMD testing needed after Single Gene Test #2?

- ◯ Yes
- ◯ No

Which additional test?

- ◯ Same test on relatives
- ◯ WES
- ◯ WGS
- ◯ Sanger seq
- ◯ Array
- ◯ Different Single Gene Test

(If you select a new test, complete that section)

Sample tracking

Relative samples at test site (or test co-ordinating site)?	◯ yes<br>◯ no<br>◯ not all samples
Notes on sample availability at testing/test co-ordinating site	__________________________________

Optional: Relative #1

Relative #1: Relation to proband	◯ mother<br>◯ father<br>◯ brother<br>◯ sister<br>◯ other
Is this Relative also affected?	◯ yes<br>◯ no
Relative #1 Study ID (IC_ABC_NNNNN)	__________________________________
Add another relative?	◯ yes<br>◯ no

Optional: Relative #2

Relative #2: Relation to proband	◯ mother<br>◯ father<br>◯ brother<br>◯ sister<br>◯ other
Is this Relative also affected?	◯ yes<br>◯ no
Relative #2 Study ID (IC_ABC_NNNNN)	__________________________________
Add another relative?	◯ yes<br>◯ no

Optional: Relative #3

Relative #3: Relation to proband	◯ mother<br>◯ father<br>◯ brother<br>◯ sister<br>◯ other
Is this Relative also affected?	◯ yes<br>◯ no

Relative #3 Study ID (IC_ABC_NNNNN) ______________________________

Add another relative?
- ◯ yes
- ◯ no

Optional: Relative #4

Relative #4: Relation to proband
- ◯ mother
- ◯ father
- ◯ brother
- ◯ sister
- ◯ other

Is this Relative also affected?
- ◯ yes
- ◯ no

Relative #4 Study ID (IC_ABC_NNNNN) ______________________________

Relatives: summary

Tick box when relative(s) single gene tests complete and their REDCap records updated?
- ☐ family single gene test 2 records completed

(Record relative's results in own REDCap record - not in proband record)

ICGNMD Review of later relative tests

Date of joint review of additional family single gene tests ______________________________ (YYYY-MM-DD)

(Proband record only) What has single gene testing of relatives added to proband's ICGNMD research findings? ______________________________

ICGNMD Whole EXOME Sequencing

Go to ICGNMD WES
- ◯ Yes
- ◯ No

Date of Joint Decision ______________________________ (YYYY-MM-DD)

Notes (e.g. justification) ______________________________

Which country will test take place in?
- ◯ UK
- ◯ India
- ◯ Turkey
- ◯ Other

(UK includes Macrogen (EU))

Details if "Other" (Country & Testing Site)	__________________________________

WES PanelApp filters

Select 1-5 PanelApp Panels to filter PDF report?	◯ Yes<br>◯ Not yet<br>(SELECT 1 to 5 PANELS)
Channelopathies	☐ Brain channelopathy<br>☐ Skeletal muscle channelopathy
Inherited Epilepsy Syndromes	☐ Genetic epilepsy syndromes
Mitochondrial	☐ Mitochondrial Disorders
Motor and Sensory Disorders of the PNS	☐ Hereditary neuropathy<br>☐ Paediatric motor neuronopathies
Motor Disorders of the CNS	☐ Cerebellar hypoplasia<br>☐ Early onset dystonia<br>☐ Hereditary spastic paraplegia<br>☐ Neurotransmitter disorders<br>☐ Structural basal ganglia disorders
Neurodegenerative Disorders	☐ Amyotrophic lateral sclerosis/motor neuron disease<br>☐ Hereditary ataxia - adult onset
Neurodevelopmental disorders	☐ Intellectual disability<br>☐ Malformations of cortical development<br>☐ Hereditary ataxia and cerebellar anomalies - childhood onset
Neuromuscular disorders	☐ Arthrogryposis<br>☐ Congenital muscular dystrophy<br>☐ Congenital myaesthenic syndrome<br>☐ Congenital myopathy<br>☐ Distal myopathies<br>☐ Limb girdle muscular dystrophy<br>☐ Rhabdomyolysis and metabolic muscle disorders
Parenchymal brain disorders	☐ Intracerebral calcification disorders
Peroxisomal disorders	☐ Peroxisomal disorders
Connective tissues disorders	☐ Ehlers Danlos syndromes
Specific metabolic abnormalities	☐ Undiagnosed metabolic disorders
List other PanelApp panels here	__________________________________<br>(Use EXACT panel names as listed at https://panelapp.genomicsengland.co.uk/panels/)

Analysis notes, e.g. specific genes to check

____
(Note: Checking specific genes NOT part of standard pipeline)

Request bespoke ICGNMD analysis?

Select bespoke ICGNMD analyses (in addition to Panels)

- ◯ Yes
- ◯ Not yet

(Please only select if essential: bespoke analyses use a lot of ICGNMD time.)

Bespoke ICGNMD analyses

- ☐ mtDNA analysis
- ☐ CNV or SV detection
- ☐ notinuse1
- ☐ notinuse2

(Please only select if essential: bespoke analyses use a lot of ICGNMD time.)

WES for relatives AT SAME TIME as proband?

Test relatives at same time?

- ◯ Yes
- ◯ No

(Proband normally has WES first, relatives later)

Justification for testing relatives at same time as proband?

____

Optional: Relative #1

Relative #1: Relation to proband

- ◯ mother
- ◯ father
- ◯ brother
- ◯ sister
- ◯ other

Is this Relative also affected?

- ◯ yes
- ◯ no

Relative #1 Study ID (IC_ABC_NNNNN)

____

Add another relative?

- ◯ yes
- ◯ no

Optional: Relative #2

Relative #2: Relation to proband	◯ mother<br>◯ father<br>◯ brother<br>◯ sister<br>◯ other
Is this Relative also affected?	◯ yes<br>◯ no
Relative #2 Study ID (IC_ABC_NNNNN)	__________________________
Add another relative?	◯ yes<br>◯ no

Optional: Relative #3

Relative #3: Relation to proband	◯ mother<br>◯ father<br>◯ brother<br>◯ sister<br>◯ other
Is this Relative also affected?	◯ yes<br>◯ no
Relative #3 Study ID (IC_ABC_NNNNN)	__________________________
Add another relative?	◯ yes<br>◯ no

Optional: Relative #4

Relative #4: Relation to proband	◯ mother<br>◯ father<br>◯ brother<br>◯ sister<br>◯ other
Is this Relative also affected?	◯ yes<br>◯ no
Relative #4 Study ID (IC_ABC_NNNNN)	__________________________

Relatives: summary

Tick box when relative(s) WES complete and their REDCap records updated	☐ family WES complete<br>(Record relative's results in own REDCap record - not in proband record)

Sample tracking

Samples available at test site (or test co-ordinating site)?	◯ yes<br>◯ no<br>◯ not all samples
Notes on sample availability at testing/test co-ordinating site	______________________
(Optional) Samples sent to approved 3rd party? E.g. UCL to Macrogen	◯ yes<br>◯ no<br>(MTA & Terms of Service must be in place prior to sending to 3rd party)
(Optional) Date samples sent to approved 3rd party	______________________<br>(YYYY-MM-DD)
Samples sent to Macrogen? (UCL only)	◯ yes<br>◯ no

WES reporting

Raw WES data received by UCL?	◯ yes<br>◯ no
Date WES data received	______________________<br>(YYYY-MM-DD)
UCL record: Data Quality Satisfactory?	◯ yes<br>◯ no
Testing site to repeat test on same sample?	◯ yes<br>◯ no
New sample needed to repeat?	◯ yes<br>◯ no
Decision = do not repeat test	◯ yes<br>◯ no
Reason for WES failure?	______________________
Data file for failed test	(OPTIONAL: upload file to show failed test or low quality outputs)

UCL ICGNMD Report for WES

UCL research report uploaded

____
(YYYY-MM-DD)

ICGNMD research report (summary PDF with filters)

ICGNMD .csv file (no panel filters)

(If requested) ICGNMD bespoke analysis report

ICGNMD Review of WES

Date of joint review of result

____
(YYYY-MM-DD)

Has WES provisionally solved?

- ◯ Yes: provisionally solved with WES
- ◯ Yes, WES confirms other test result
- ◯ No, WES has not supported a research finding

Causative variant #1

Condition #1 [Orphanet]

____

Free text box if Orphanet not suitable

____

Caustive gene & variant #1 [OMIM]

____

Free text box if OMIM not suitable

____

Known gene mutation 1 - VCF

____
(Use format (CHR POS ID REF ALT): 1 230710048 rs699 A G)

WES variant results #1 - freetext notes

____

Variant #1 Provisional classification by ICGNMD/ACMG

- ◯ Pathogenic
- ◯ Likely Pathogenic
- ◯ VUS - Suspected Pathogenic
- ◯ VUS

Add another variant?

- ◯ yes
- ◯ no

Causative variant #2

Condition #2 [Orphanet] ________________________________

Free text box if Orphanet not suitable ________________________________

Caustive gene & variant #2 [OMIM] ________________________________

Free text box if OMIM not suitable ________________________________

Known gene mutation 2 - VCF ________________________________
(Use format (CHR POS ID REF ALT): 1 230710048 rs699 A G)

Variant #2 Provisional classification by ICGNMD/ACMG
- ◯ Pathogenic
- ◯ Likely Pathogenic
- ◯ VUS - Suspected Pathogenic
- ◯ VUS

WES variant #2 results - freetext notes ________________________________

Add another variant?
- ◯ yes
- ◯ no

Causative variant #3

Condition #3 [Orphanet] ________________________________

Free text box if Orphanet not suitable ________________________________

Caustive gene & variant #3 [OMIM] ________________________________

Free text box if OMIM not suitable ________________________________

Known gene mutation 3 - VCF ________________________________
(Use format (CHR POS ID REF ALT): 1 230710048 rs699 A G)

Variant #3 Provisional classification by ICGNMD/ACMG
- ◯ Pathogenic
- ◯ Likely Pathogenic
- ◯ VUS - Suspected Pathogenic
- ◯ VUS

WES variant #3 results - freetext notes ________________________________

Add another variant?	◯ yes<br>◯ no

Causative variant #4

Condition #4 [Orphanet]	__________________________
Free text box if Orphanet not suitable	__________________________
Caustive gene & variant #4 [OMIM]	__________________________
Free text box if OMIM not suitable	__________________________
Known gene mutation 4 - VCF	__________________________<br>(Use format (CHR POS ID REF ALT): 1 230710048 rs699 A G)
Variant #4 Provisional classification by ICGNMD/ACMG	◯ Pathogenic<br>◯ Likely Pathogenic<br>◯ VUS - Suspected Pathogenic<br>◯ VUS
WES variant #4 results - freetext notes	__________________________

ICGNMD Next steps

More ICGNMD testing needed after WES review?	◯ Yes<br>◯ No
Which additional test?	◯ WES on relatives<br>◯ WGS<br>◯ Sanger seq<br>◯ Array<br>◯ Single Gene Test<br>◯ Reanalyse WES data<br>(If you select a new test, complete that section)

Sample tracking

Relative samples at test site (or test co-ordinating site)?	◯ yes<br>◯ no<br>◯ not all samples
Notes on sample availability at testing/test co-ordinating site	__________________________

Optional: Relative #1

Relative #1: Relation to proband	◯ mother<br>◯ father<br>◯ brother<br>◯ sister<br>◯ other
Is this Relative also affected?	◯ yes<br>◯ no
Relative #1 Study ID (IC_ABC_NNNNN)	__________________________________
Add another relative?	◯ yes<br>◯ no

Optional: Relative #2

Relative #2: Relation to proband	◯ mother<br>◯ father<br>◯ brother<br>◯ sister<br>◯ other
Is this Relative also affected?	◯ yes<br>◯ no
Relative #2 Study ID (IC_ABC_NNNNN)	__________________________________
Add another relative?	◯ yes<br>◯ no

Optional: Relative #3

Relative #3: Relation to proband	◯ mother<br>◯ father<br>◯ brother<br>◯ sister<br>◯ other
Is this Relative also affected?	◯ yes<br>◯ no
Relative #3 Study ID (IC_ABC_NNNNN)	__________________________________
Add another relative?	◯ yes<br>◯ no

Optional: Relative #4

Relative #4: Relation to proband	◯ mother<br>◯ father<br>◯ brother<br>◯ sister<br>◯ other
Is this Relative also affected?	◯ yes<br>◯ no
Relative #4 Study ID (IC_ABC_NNNNN)	______________________

Relatives: summary

Tick box when relative(s) WES complete and their REDCap records updated	☐ family WES complete & REDCap updated<br>(Record relative's results in own REDCap record - not in proband record)

ICGNMD Review of later relative tests

Date of joint review of additional family single gene tests	______________________<br>(YYYY-MM-DD)
(Proband record only) What has later WES of relatives added to proband's ICGNMD research findings?	______________________

ICGNMD repeat WES analysis

Reason for repeating WES analysis?	◯ Long time since 1st analysis, check for new variants<br>◯ New bespoke analysis available<br>◯ Other
Expand with further details here, e.g specific genes to check	______________________<br>(Note: Checking specific genes NOT part of standard pipeline)

UCL ICGNMD WES Re-analysis

New research report created	______________________<br>(YYYY-MM-DD)
ICGNMD new research report (summary PDF with filters)	
ICGNMD new .csv file (no panel filters)	
(If requested) new ICGNMD bespoke analysis report	

ICGNMD Review of WES Re-analysis

Date of joint review of re-analysis

____
(YYYY-MM-DD)

Has WES provisionally solved?

- ◯ Yes: provisionally solved with WES
- ◯ Yes, WES confirms other test result
- ◯ No, WES has not supported a research finding

Causative variant #1

Condition #1 [Orphanet]

____

Free text box if Orphanet not suitable

____

Caustive gene & variant #1 [OMIM]

____

Free text box if OMIM not suitable

____

Known gene mutation 1 - VCF

____
(Use format (CHR POS ID REF ALT): 1 230710048 rs699 A G)

WES variant results #1 - freetext notes

____

Variant #1 Provisional classification by ICGNMD/ACMG

- ◯ Pathogenic
- ◯ Likely Pathogenic
- ◯ VUS - Suspected Pathogenic
- ◯ VUS

Add another variant?

- ◯ yes
- ◯ no

Causative variant #2

Condition #2 [Orphanet]

____

Free text box if Orphanet not suitable

____

Caustive gene & variant #2 [OMIM]

____

Free text box if OMIM not suitable

____

Known gene mutation 2 - VCF

____
(Use format (CHR POS ID REF ALT): 1 230710048 rs699 A G)

Variant #2 Provisional classification by ICGNMD/ACMG

◯ Pathogenic
◯ Likely Pathogenic
◯ VUS - Suspected Pathogenic
◯ VUS

WES variant #2 results - freetext notes

____

Add another variant?

◯ yes
◯ no

Causative variant #3

Condition #3 [Orphanet]

____

Free text box if Orphanet not suitable

____

Caustive gene & variant #3 [OMIM]

____

Free text box if OMIM not suitable

____

Known gene mutation 3 - VCF

____
(Use format (CHR POS ID REF ALT): 1 230710048 rs699 A G)

Variant #3 Provisional classification by ICGNMD/ACMG

◯ Pathogenic
◯ Likely Pathogenic
◯ VUS - Suspected Pathogenic
◯ VUS

WES variant #3 results - freetext notes

____

Add another variant?

◯ yes
◯ no

Causative variant #4

Condition #4 [Orphanet] ______________________________

Free text box if Orphanet not suitable ______________________________

Caustive gene & variant #4 [OMIM] ______________________________

Free text box if OMIM not suitable ______________________________

Known gene mutation 4 - VCF ______________________________
(Use format (CHR POS ID REF ALT): 1 230710048 rs699 A G)

Variant #4 Provisional classification by ICGNMD/ACMG
- ◯ Pathogenic
- ◯ Likely Pathogenic
- ◯ VUS - Suspected Pathogenic
- ◯ VUS

WES variant #4 results - freetext notes ______________________________

ICGNMD Whole GENOME sequencing

Go to ICGNMD WGS
- ◯ Yes
- ◯ No

Date of Joint Decision ______________________________
(YYYY-MM-DD)

Notes (e.g. justification) ______________________________

Which country will test take place in?
- ◯ UK Macrogen
- ◯ UK Illumina
- ◯ India
- ◯ Turkey
- ◯ Other

Details if "Other" (Country & Testing Site) ______________________________

WGS PanelApp filters

Select 1-5 PanelApp Panels to filter PDF report?	◯ Yes<br>◯ Not yet<br>(SELECT 1 to 5 PANELS)
Channelopathies	☐ Brain channelopathy<br>☐ Skeletal muscle channelopathy
Inherited Epilepsy Syndromes	☐ Genetic epilepsy syndromes
Mitochondrial	☐ Mitochondrial Disorders
Motor and Sensory Disorders of the PNS	☐ Hereditary neuropathy<br>☐ Paediatric motor neuronopathies
Motor Disorders of the CNS	☐ Cerebellar hypoplasia<br>☐ Early onset dystonia<br>☐ Hereditary spastic paraplegia<br>☐ Neurotransmitter disorders<br>☐ Structural basal ganglia disorders
Neurodegenerative Disorders	☐ Amyotrophic lateral sclerosis/motor neuron disease<br>☐ Hereditary ataxia - adult onset
Neurodevelopmental disorders	☐ Intellectual disability<br>☐ Malformations of cortical development<br>☐ Hereditary ataxia and cerebellar anomalies - childhood onset
Neuromuscular disorders	☐ Arthrogryposis<br>☐ Congenital muscular dystrophy<br>☐ Congenital myaesthenic syndrome<br>☐ Congenital myopathy<br>☐ Distal myopathies<br>☐ Limb girdle muscular dystrophy<br>☐ Rhabdomyolysis and metabolic muscle disorders
Parenchymal brain disorders	☐ Intracerebral calcification disorders
Peroxisomal disorders	☐ Peroxisomal disorders
Connective tissues disorders	☐ Ehlers Danlos syndromes
Specific metabolic abnormalities	☐ Undiagnosed metabolic disorders
List other PanelApp panels here	______________________________<br>(Use EXACT panel names as listed at https://panelapp.genomicsengland.co.uk/panels/)
Additional WGS notes, e.g. specific genes to check	______________________________<br>(Note: Checking specific genes NOT part of standard pipeline)

Request bespoke ICGNMD analysis?

Select bespoke ICGNMD analyses (in addition to Panels)	◯ Yes<br>◯ Not yet<br>(Please only select if essential: bespoke analyses use a lot of ICGNMD time.)
Bespoke ICGNMD analyses	☐ mtDNA analysis<br>☐ CNV or SV detection<br>☐ notinuse1<br>☐ notinuse2<br>(Please only select if essential: bespoke analyses use a lot of ICGNMD time.)

WGS for relatives AT SAME TIME as proband?

Test relatives at same time?	◯ Yes<br>◯ No<br>(Proband normally has wgs first, relatives later)
Justification for testing relatives at same time as proband?	______________________________

Optional: Relative #1

Relative #1: Relation to proband	◯ mother<br>◯ father<br>◯ brother<br>◯ sister<br>◯ other
Is this Relative also affected?	◯ yes<br>◯ no
Relative #1 Study ID (IC_ABC_NNNNN)	______________________________
Add another relative?	◯ yes<br>◯ no

Optional: Relative #2

Relative #2: Relation to proband	◯ mother<br>◯ father<br>◯ brother<br>◯ sister<br>◯ other
Is this Relative also affected?	◯ yes<br>◯ no
Relative #2 Study ID (IC_ABC_NNNNN)	______________________________

Add another relative?	◯ yes<br>◯ no

Optional: Relative #3

Relative #3: Relation to proband	◯ mother<br>◯ father<br>◯ brother<br>◯ sister<br>◯ other
Is this Relative also affected?	◯ yes<br>◯ no
Relative #3 Study ID (IC_ABC_NNNNN)	__________________________________
Add another relative?	◯ yes<br>◯ no

Optional: Relative #4

Relative #4: Relation to proband	◯ mother<br>◯ father<br>◯ brother<br>◯ sister<br>◯ other
Is this Relative also affected?	◯ yes<br>◯ no
Relative #4 Study ID (IC_ABC_NNNNN)	__________________________________

Relatives: summary

Tick box when relative(s) WGS complete and their REDCap records updated	☐ family WGS complete<br>(Record relative's results in own REDCap record - not in proband record)

Sample tracking

Samples available at test site (or test co-ordinating site)?	◯ yes<br>◯ no<br>◯ not all samples
Notes on sample availability at testing/test co-ordinating site	__________________________________
(Optional) Samples sent to approved 3rd party? E.g. UCL to Macrogen/Illumina	◯ yes<br>◯ no<br>(MTA & Terms of Service must be in place prior to sending to 3rd party)

(Optional) Date samples sent to approved 3rd party

____
(YYYY-MM-DD)

Samples sent to Macrogen/Illumina? (UCL only)
○ yes
○ no

WGS reporting

Raw WGS data received by UCL?
○ yes
○ no

Date WGS data received

____
(YYYY-MM-DD)

UCL record: Data Quality Satisfactory?
○ yes
○ no

Testing site to repeat test on same sample?
○ yes
○ no

New sample needed to repeat?
○ yes
○ no

Decision = do not repeat test
○ yes
○ no

Reason for WGS failure?

____

Data file for failed test

(OPTIONAL: upload file to show failed test or low quality outputs)

UCL ICGNMD Report for WGS

UCL research report uploaded

____
(YYYY-MM-DD)

ICGNMD research report (summary PDF with filters)

ICGNMD .csv file (no panel filters)

(If requested) ICGNMD bespoke analysis report

ICGNMD Review of WGS

Date of joint review of result

____
(YYYY-MM-DD)

Has WGS provisionally solved?

- ◯ Yes: provisionally solved with WGS
- ◯ Yes, WGS confirms other test result
- ◯ No, WGS has not supported a research finding

Causative variant #1

Condition #1 [Orphanet]

____

Free text box if Orphanet not suitable

____

Caustive gene & variant #1 [OMIM]

____

Free text box if OMIM not suitable

____

Known gene mutation 1 - VCF

____
(Use format (CHR POS ID REF ALT): 1 230710048 rs699 A G)

WGS variant results #1 - freetext notes

____

Variant #1 Provisional classification by ICGNMD/ACMG

- ◯ Pathogenic
- ◯ Likely Pathogenic
- ◯ VUS - Suspected Pathogenic
- ◯ VUS

Add another variant?

- ◯ yes
- ◯ no

Causative variant #2

Condition #2 [Orphanet]

____

Free text box if Orphanet not suitable

____

Caustive gene & variant #2 [OMIM]

____

Free text box if OMIM not suitable

____

Known gene mutation 2 - VCF

____
(Use format (CHR POS ID REF ALT): 1 230710048 rs699 A G)

Variant #2 Provisional classification by ICGNMD/ACMG

◯ Pathogenic
◯ Likely Pathogenic
◯ VUS - Suspected Pathogenic
◯ VUS

WGS variant #2 results - freetext notes

____

Add another variant?

◯ yes
◯ no

Causative variant #3

Condition #3 [Orphanet]

____

Free text box if Orphanet not suitable

____

Caustive gene & variant #3 [OMIM]

____

Free text box if OMIM not suitable

____

Known gene mutation 3 - VCF

____
(Use format (CHR POS ID REF ALT): 1 230710048 rs699 A G)

Variant #3 Provisional classification by ICGNMD/ACMG

◯ Pathogenic
◯ Likely Pathogenic
◯ VUS - Suspected Pathogenic
◯ VUS

WGS variant #3 results - freetext notes

____

Add another variant?

◯ yes
◯ no

Causative variant #4

Condition #4 [Orphanet] __________________________________

Free text box if Orphanet not suitable __________________________________

Caustive gene & variant #4 [OMIM] __________________________________

Free text box if OMIM not suitable __________________________________

Known gene mutation 4 - VCF __________________________________
(Use format (CHR POS ID REF ALT): 1 230710048 rs699 A G)

Variant #4 Provisional classification by ICGNMD/ACMG
- ◯ Pathogenic
- ◯ Likely Pathogenic
- ◯ VUS - Suspected Pathogenic
- ◯ VUS

WGS variant #4 results - freetext notes __________________________________

ICGNMD Next steps

More ICGNMD testing needed after WGS review?
- ◯ Yes
- ◯ No

Which additional test?
- ◯ WGS on relatives
- ◯ WGS
- ◯ Sanger seq
- ◯ Array
- ◯ Single Gene Test
- ◯ Reanalyse WGS data

(If you select a new test, complete that section)

Sample tracking

Relative samples at test site (or test co-ordinating site)?
- ◯ yes
- ◯ no
- ◯ not all samples

Notes on sample availability at testing/test co-ordinating site ____

Optional: Relative #1

Relative #1: Relation to proband	◯ mother<br>◯ father<br>◯ brother<br>◯ sister<br>◯ other
Is this Relative also affected?	◯ yes<br>◯ no
Relative #1 Study ID (IC_ABC_NNNNN)	__________________________________
Add another relative?	◯ yes<br>◯ no

Optional: Relative #2

Relative #2: Relation to proband	◯ mother<br>◯ father<br>◯ brother<br>◯ sister<br>◯ other
Is this Relative also affected?	◯ yes<br>◯ no
Relative #2 Study ID (IC_ABC_NNNNN)	__________________________________
Add another relative?	◯ yes<br>◯ no

Optional: Relative #3

Relative #3: Relation to proband	◯ mother<br>◯ father<br>◯ brother<br>◯ sister<br>◯ other
Is this Relative also affected?	◯ yes<br>◯ no
Relative #3 Study ID (IC_ABC_NNNNN)	__________________________________
Add another relative?	◯ yes<br>◯ no

Optional: Relative #4

Relative #4: Relation to proband
- ◯ mother
- ◯ father
- ◯ brother
- ◯ sister
- ◯ other

Is this Relative also affected?
- ◯ yes
- ◯ no

Relative #4 Study ID (IC_ABC_NNNNN)

____

Relatives: summary

Tick box when relative(s) WGS complete and their REDCap records updated

☐ family WGS complete & REDCap records updated (Record relative's results in own REDCap record - not in proband record)

ICGNMD Review of later relative tests

Date of joint review of additional family single gene tests

____
(YYYY-MM-DD)

(Proband record only) What has later WGS of relatives added to proband's ICGNMD research findings?

____

ICGNMD repeat WGS analysis

Reason for repeating WGS analysis?
- ◯ Long time since 1st analysis, check for new variants
- ◯ New bespoke analysis available
- ◯ Other

Expand with further details here

____

UCL ICGNMD WGS Re-analysis

New research report created

____
(YYYY-MM-DD)

ICGNMD new research report (summary PDF with filters)

ICGNMD new .csv file (no panel filters)

(If requested) new ICGNMD bespoke analysis report

ICGNMD Review of WGS Re-analysis

Date of joint review of re-analysis

____
(YYYY-MM-DD)

Has WGS provisionally solved?

- ◯ Yes: provisionally solved with WGS
- ◯ Yes, WGS confirms other test result
- ◯ No, WGS has not supported a research finding

Causative variant #1

Condition #1 [Orphanet]

____

Free text box if Orphanet not suitable

____

Caustive gene & variant #1 [OMIM]

____

Free text box if OMIM not suitable

____

Known gene mutation 1 - VCF

____
(Use format (CHR POS ID REF ALT): 1 230710048 rs699 A G)

WGS variant results #1 - freetext notes

____

Variant #1 Provisional classification by ICGNMD/ACMG

- ◯ Pathogenic
- ◯ Likely Pathogenic
- ◯ VUS - Suspected Pathogenic
- ◯ VUS

Add another variant?

- ◯ yes
- ◯ no

Causative variant #2

Condition #2 [Orphanet]

____

Free text box if Orphanet not suitable

____

Caustive gene & variant #2 [OMIM]

____

Free text box if OMIM not suitable

____

Known gene mutation 2 - VCF

____
(Use format (CHR POS ID REF ALT): 1 230710048 rs699 A G)

Variant #2 Provisional classification by ICGNMD/ACMG

- ◯ Pathogenic
- ◯ Likely Pathogenic
- ◯ VUS - Suspected Pathogenic
- ◯ VUS

WGS variant #2 results - freetext notes

____

Add another variant?

- ◯ yes
- ◯ no

Causative variant #3

Condition #3 [Orphanet]

____

Free text box if Orphanet not suitable

____

Caustive gene & variant #3 [OMIM]

____

Free text box if OMIM not suitable

____

Known gene mutation 3 - VCF

____
(Use format (CHR POS ID REF ALT): 1 230710048 rs699 A G)

Variant #3 Provisional classification by ICGNMD/ACMG

- ◯ Pathogenic
- ◯ Likely Pathogenic
- ◯ VUS - Suspected Pathogenic
- ◯ VUS

WGS variant #3 results - freetext notes

____

Add another variant?

- ◯ yes
- ◯ no

Causative variant #4

Condition #4 [Orphanet] __________________________________

Free text box if Orphanet not suitable __________________________________

Caustive gene & variant #4 [OMIM] __________________________________

Free text box if OMIM not suitable __________________________________

Known gene mutation 4 - VCF __________________________________
(Use format (CHR POS ID REF ALT): 1 230710048 rs699 A G)

Variant #4 Provisional classification by ICGNMD/ACMG
- ◯ Pathogenic
- ◯ Likely Pathogenic
- ◯ VUS - Suspected Pathogenic
- ◯ VUS

WGS variant #4 results - freetext notes __________________________________

ICGNMD Sanger Sequencing

Go to ICGNMD Sanger Sequencing?
- ◯ Yes
- ◯ No

(Complete ONLY if ICGNMD to fund Sanger)

Date of Joint Decision __________________________________
(YYYY-MM-DD)

Discussion notes ____
(Most recent at top, include date & initials)

Which country will test take place in?
- ◯ UK (or Leiden for some FSHD)
- ◯ India
- ◯ Brazil
- ◯ Other

Gene & variant to be tested by Sanger Seq (provide VCF and OMIM terms)

Variant to test in VCF format

____
(Use format (CHR POS ID REF ALT): 1 230710048 rs699 A G)

Variant to test [OMIM]

____

Free text box if OMIM not suitable

____

Testing notes (e.g. justification, conditions, protocol refs)

____

Same Sanger seq for relatives AT SAME TIME as proband?

Test relatives at same time as proband?

- ○ Yes
- ○ No

Optional: Relative #1

Relative #1: Relation to proband

- ○ mother
- ○ father
- ○ brother
- ○ sister
- ○ other

Is this Relative also affected?

- ○ yes
- ○ no

Relative #1 Study ID (IC_ABC_NNNNN)

____

Add another relative?

- ○ yes
- ○ no

Optional: Relative #2

Relative #2: Relation to proband

- ○ mother
- ○ father
- ○ brother
- ○ sister
- ○ other

Is this Relative also affected?

- ○ yes
- ○ no

Relative #2 Study ID (IC_ABC_NNNNN)

____

Add another relative?

- ○ yes
- ○ no

Optional: Relative #3

Relative #3: Relation to proband	◯ mother<br>◯ father<br>◯ brother<br>◯ sister<br>◯ other
Is this Relative also affected?	◯ yes<br>◯ no
Relative #3 Study ID (IC_ABC_NNNNN)	__________________________________
Add another relative?	◯ yes<br>◯ no

Optional: Relative #4

Relative #4: Relation to proband	◯ mother<br>◯ father<br>◯ brother<br>◯ sister<br>◯ other
Is this Relative also affected?	◯ yes<br>◯ no
Relative #4 Study ID (IC_ABC_NNNNN)	__________________________________

Relatives: summary

Tick box when relative(s) Sanger complete and their REDCap records updated	☐ family Sanger test 1 complete<br>(Record relative's results in own REDCap record - not in proband record)

Sample tracking

Samples available at test site (or test co-ordinating site)?	◯ yes<br>◯ no
Notes on sample availability at testing/test co-ordinating site	__________________________________
(Optional) Samples sent to approved 3rd party test lab?	◯ yes<br>◯ no<br>(MTA & Terms of Service must be in place prior to sending to 3rd party)
(Optional) Date samples sent to approved 3rd party	____________________________<br>(YYYY-MM-DD)

After-test reporting

Result Quality Satisfactory?
- ◯ yes
- ◯ no

Testing site to repeat test on same sample?
- ◯ yes
- ◯ no

New sample needed to repeat?
- ◯ yes
- ◯ no

Decision = do not repeat test
- ◯ yes
- ◯ no

Reason for Sanger failure?

____

Data file for failed test

(OPTIONAL: upload file to show failed test or low quality outputs)

Sanger seq Results file (research only)

(Upload/View Single Gene Test report)

Sanger seq test: results from test site
- ◯ Supports diagnosis
- ◯ Partially supports diagnosis
- ◯ Does not support diagnosis
- ◯ Inconclusive result

UCL ICGNMD Report for Sanger Sequencing

UCL research report uploaded

____
(YYYY-MM-DD)

ICGNMD research report

ICGNMD Review of Sanger sequencing result

Date of joint review of result

____
(YYYY-MM-DD)

Known gene mutation - VCF

____
(Use format (CHR POS ID REF ALT): 1 230710048 rs699 A G)

Condition [Orphanet]

____

Free text box if Orphanet not suitable

____

Caustive gene & variant [OMIM]

____

Free text box if OMIM not suitable	__________________________
Sanger seq results - freetext notes	__________________________
Provisional classification by ICGNMD/ACMG	◯ Pathogenic<br>◯ Likely Pathogenic<br>◯ VUS - Suspected Pathogenic<br>◯ VUS
Has Sanger seq provisionally solved?	◯ Yes: provisionally solved with this test<br>◯ Yes, this test confirms other test result<br>◯ No, this test has not supported a research finding

ICGNMD Next steps

More ICGNMD testing needed after Sanger seq?	◯ Yes<br>◯ No
Which additional test?	◯ Same test on relatives<br>◯ WES<br>◯ WGS<br>◯ Array<br>◯ Single Gene Test<br>(If you select a new test, complete that section)

Sample tracking

Relative samples at test site (or test co-ordinating site)?	◯ yes<br>◯ no<br>◯ not all samples
Notes on sample availability at testing/test co-ordinating site	__________________________

Optional: Relative #1

Relative #1: Relation to proband	◯ mother<br>◯ father<br>◯ brother<br>◯ sister<br>◯ other
Is this Relative also affected?	◯ yes<br>◯ no
Relative #1 Study ID (IC_ABC_NNNNN)	__________________________
Add another relative?	◯ yes<br>◯ no

Optional: Relative #2

Relative #2: Relation to proband	◯ mother<br>◯ father<br>◯ brother<br>◯ sister<br>◯ other
Is this Relative also affected?	◯ yes<br>◯ no
Relative #2 Study ID (IC_ABC_NNNNN)	______________________
Add another relative?	◯ yes<br>◯ no

Optional: Relative #3

Relative #3: Relation to proband	◯ mother<br>◯ father<br>◯ brother<br>◯ sister<br>◯ other
Is this Relative also affected?	◯ yes<br>◯ no
Relative #3 Study ID (IC_ABC_NNNNN)	______________________
Add another relative?	◯ yes<br>◯ no

Optional: Relative #4

Relative #4: Relation to proband	◯ mother<br>◯ father<br>◯ brother<br>◯ sister<br>◯ other
Is this Relative also affected?	◯ yes<br>◯ no
Relative #4 Study ID (IC_ABC_NNNNN)	______________________
Relative Sanger complete and relatives' REDCap records updated?	◯ yes<br>◯ no<br>(Record relative's results in own REDCap record - not in proband record)

ICGNMD Review of later relative tests

Date of joint review of additional family Sanger seq

____
(YYYY-MM-DD)

(Proband record only) What has Sanger seq of relatives added to proband's ICGNMD research findings?

____

ICGNMD Array

Go to ICGNMD Array?

◯ Yes
◯ No
(Complete ONLY if ICGNMD to fund array)

Date of Joint Decision

____
(YYYY-MM-DD)

Reason for Array

◯ CNV analysis
◯ Multigenerational testing/linkage
◯ Other

If "Other", enter reasons here

____

Which country will test take place in?

◯ UK
◯ India
◯ Other

If Other, enter details here (country & name of test provider)

____

Array product details (e.g. manufacturer, product code, batch #)

____

Array for relatives AT SAME TIME as proband?

Test relatives as well?

◯ Yes
◯ No

Optional: Relative #1

Relative #1: Relation to proband

◯ mother
◯ father
◯ brother
◯ sister
◯ other

Is this Relative also affected?

◯ yes
◯ no

Relative #1 Study ID (IC_ABC_NNNNN)	______________________
Add another relative?	◯ yes<br>◯ no

Optional: Relative #2

Relative #2: Relation to proband	◯ mother<br>◯ father<br>◯ brother<br>◯ sister<br>◯ other
Is this Relative also affected?	◯ yes<br>◯ no
Relative #2 Study ID (IC_ABC_NNNNN)	______________________
Add another relative?	◯ yes<br>◯ no

Optional: Relative #3

Relative #3: Relation to proband	◯ mother<br>◯ father<br>◯ brother<br>◯ sister<br>◯ other
Is this Relative also affected?	◯ yes<br>◯ no
Relative #3 Study ID (IC_ABC_NNNNN)	______________________
Add another relative?	◯ yes<br>◯ no

Optional: Relative #4

Relative #4: Relation to proband	◯ mother<br>◯ father<br>◯ brother<br>◯ sister<br>◯ other
Is this Relative also affected?	◯ yes<br>◯ no

Relative #4 Study ID (IC_ABC_NNNNN)	______________________

Relatives: summary

Tick box when relative(s) array complete and their REDCap records updated?	☐ family array complete<br>(Record relative's results in own REDCap record - not in proband record)

Sample tracking

Samples available at test site (or test co-ordinating site)?	○ yes<br>○ no
Notes on sample availability at testing/test co-ordinating site	______________________
(Optional) Samples sent to approved 3rd party test lab?	○ yes<br>○ no<br>(MTA & Terms of Service must be in place prior to sending to 3rd party)
(Optional) Date samples sent to approved 3rd party	______________________<br>(YYYY-MM-DD)

After-test reporting

Result Quality Satisfactory?	○ yes<br>○ no
Testing site to repeat test on same sample?	○ yes<br>○ no
New sample needed to repeat?	○ yes<br>○ no
Decision = do not repeat test	○ yes<br>○ no
Reason for Array failure?	______________________
Data file for failed test	(OPTIONAL: upload file to show failed test or low quality outputs)
Array Results file (research only)	(Upload/View Single Gene Test report)

Array: results from test site

◯ Supports diagnosis
◯ Partially supports diagnosis
◯ Does not support diagnosis
◯ Inconclusive result

UCL ICGNMD Report for Array

UCL research report uploaded

____
(YYYY-MM-DD)

ICGNMD research report

ICGNMD Review of Array

Date of joint review of result

____
(YYYY-MM-DD)

Has Array provisionally solved or helped to solve?

◯ Yes: provisionally solved with this test
◯ Yes, this test confirms other test result
◯ No, this test has not supported a research finding

Causative variant #1

Known gene mutation - VCF

____
(Use format (CHR POS ID REF ALT): 1 230710048 rs699 A G)

Condition #1 [Orphanet]

____

Caustive gene & variant #1 [OMIM]

____

Array variant #1 results - freetext notes

____

Provisional classification by ICGNMD

◯ Pathogenic
◯ Likely Pathogenic
◯ VUS - Suspected Pathogenic
◯ VUS

Add another variant?

◯ yes
◯ no

Causative variant #2

Known gene mutation 2 - VCF

____
(Use format (CHR POS ID REF ALT): 1 230710048 rs699 A G)

Condition #2 [Orphanet]

____

Caustive gene & variant #2 [OMIM]

____

Variant #2 Provisional classification by ICGNMD

◯ Pathogenic
◯ Likely Pathogenic
◯ VUS - Suspected Pathogenic
◯ VUS

Array variant #2 results - freetext notes

____

Add another variant?

◯ yes
◯ no

Causative variant #3

Condition #3 [Orphanet]

____

Caustive gene & variant #3 [OMIM]

____

Known gene mutation 3 - VCF

____
(Use format (CHR POS ID REF ALT): 1 230710048 rs699 A G)

Variant #3 Provisional classification by ICGNMD

◯ Pathogenic
◯ Likely Pathogenic
◯ VUS - Suspected Pathogenic
◯ VUS

Array variant #3 results - freetext notes

____

ICGNMD Next steps

More ICGNMD testing needed after Array?

◯ Yes
◯ No

Which additional test?

◯ Array on relatives
◯ WES
◯ WGS
◯ Sanger seq
◯ Single Gene Test
(If you select a new test, complete that section)

Sample tracking	
Relative samples at test site (or test co-ordinating site)?	◯ yes<br>◯ no<br>◯ not all samples
Notes on sample availability at testing/test co-ordinating site	____________________

Optional: Relative #1	
Relative #1: Relation to proband	◯ mother<br>◯ father<br>◯ brother<br>◯ sister<br>◯ other
Is this Relative also affected?	◯ yes<br>◯ no
Relative #1 Study ID (IC_ABC_NNNNN)	____________________
Add another relative?	◯ yes<br>◯ no

Optional: Relative #2	
Relative #2: Relation to proband	◯ mother<br>◯ father<br>◯ brother<br>◯ sister<br>◯ other
Is this Relative also affected?	◯ yes<br>◯ no
Relative #2 Study ID (IC_ABC_NNNNN)	____________________
Add another relative?	◯ yes<br>◯ no

Optional: Relative #3	
Relative #3: Relation to proband	◯ mother<br>◯ father<br>◯ brother<br>◯ sister<br>◯ other
Is this Relative also affected?	◯ yes<br>◯ no

Relative #3 Study ID (IC_ABC_NNNNN) ______________________________

Add another relative?
- ◯ yes
- ◯ no

Optional: Relative #4

Relative #4: Relation to proband
- ◯ mother
- ◯ father
- ◯ brother
- ◯ sister
- ◯ other

Is this Relative also affected?
- ◯ yes
- ◯ no

Relative #4 Study ID (IC_ABC_NNNNN) ______________________________

Relative(s) arrays complete and their REDCap records updated?
- ◯ yes
- ◯ no

(Record relative's results in own REDCap record - not in proband record)

ICGNMD Review of later relative tests

Date of joint review of additional family Arrays ______________________________ (YYYY-MM-DD)

(Proband record only) What has Array of relatives added to proband's ICGNMD research findings? ______________________________

Final ICGNMD Research Summary (UCL: complete at end of pathway)

Reason for issuing final report now
- ◯ No further testing required
- ◯ Further testing desirable, but not possible under current project
- ◯ No ICGNMD report, recruited as "solved" participant

Participant ICGNMD outcome (research only) - Select any that apply
- ☐ Genetically solved with no ICGNMD input
- ☐ Outcome supported with ICGNMD intellectual input
- ☐ Outcome supported by ICGNMD genetic testing
- ☐ Local AND ICGNMD genetic tests supported outcome
- ☐ No causative/likely causative variants identified
- ☐ Most suitable test not available locally or via ICGNMD
- ☐ Study ended before testing

Which ICGNMD genetic test(s) gave positive research result? (Select any that apply)	☐ WGS - proband<br>☐ WES - proband<br>☐ Array - proband<br>☐ Single Gene Test - proband<br>☐ Sanger seq - proband<br>☐ WGS - relatives<br>☐ WES - relatives<br>☐ Array - relatives<br>☐ Single gene test - relatives<br>☐ Sanger seq - relatives<br>☐ Other<br>☐ None
Further details	______________________________

Final ICGNMD diagnosis (research only)

Final diagnostic category	◯ Genetic motor neuron disease<br>◯ Spinal muscular atrophy<br>◯ Genetic peripheral neuropathy<br>◯ Congenital myasthenic syndrome<br>◯ Congenital muscular dystrophy or myopathy<br>◯ Duchenne or Becker muscular dystrophy<br>◯ Facioscapulohumeral muscular dystrophy<br>◯ Myotonic dystrophy type I<br>◯ Limb girdle muscular dystrophy<br>◯ Distal myopathy<br>◯ Adult onset myopathy<br>◯ Metabolic myopathy<br>◯ Mitochondrial disease<br>◯ Skeletal muscle channelopathy<br>◯ Undetermined genetic neuromuscular or neurological disorder<br>(Diagnosis most likely, after ICGNMD pathway complete. DO NOT COMPLETE FOR PARTICIPANTS RECRUITED AS SOLVED CASES)
Condition #1	________________________<br>((Orphanet) You can add extra diagnoses below if complex (e.g. "double trouble"))
Other Condition #2	________________________<br>(Orphanet)
Other Condition #3	________________________<br>(Orphanet)
Confirmed diagnoses - freetext	______________________________
Has original "best guess" diagnosis changed with ICGNMD?	◯ yes<br>◯ no<br>◯ not clear

Final Causative variant #1

Variant #1 ICGNMD Classification	◯ Pathogenic<br>◯ Likely Pathogenic<br>◯ VUS - Suspected Pathogenic
Causative variant #1 - VCF	__________________________________<br>(Use format (CHR POS ID REF ALT): 1 230710048 rs699 A G)
Condition #1 [Orphanet]	__________________________________
Caustive gene & variant #1 [OMIM]	__________________________________
Variant #1 - freetext notes	__________________________________
Add another variant?	◯ yes<br>◯ no

Final Causative variant #2

Variant #2 ICGNMD Classification	◯ Pathogenic<br>◯ Likely Pathogenic<br>◯ VUS - Suspected Pathogenic
Causative variant #2 - VCF	__________________________________<br>(Use format (CHR POS ID REF ALT): 1 230710048 rs699 A G)
Condition #2 [Orphanet]	__________________________________
Caustive gene & variant #2 [OMIM]	__________________________________
Variant #2 - freetext notes	__________________________________
Add another variant?	◯ yes<br>◯ no

Final Causative variant #3

Variant #3 ICGNMD Classification	◯ Pathogenic<br>◯ Likely Pathogenic<br>◯ VUS - Suspected Pathogenic
Causative variant #3 - VCF	__________________________________<br>(Use format (CHR POS ID REF ALT): 1 230710048 rs699 A G)

Condition #3 [Orphanet] ______________________

Caustive gene & variant #3 [OMIM] ______________________

Variant #3 - freetext notes ______________________

Final ICGNMD Report (Research only, not diagnostic)

Upload final ICGNMD report

ICGNMD comments for follow-on research

Select one option
- ◯ Variant(s) already well-known & good fit to phenotype
- ◯ Novel, suspected pathogenic variant in established disease gene
- ◯ Variant(s) known pathogenic but phenotype novel features
- ◯ Novel suspected variant in gene not linked to this disease
- ◯ Other

Details ______________________

Participant's data despoited in archive?

- ◯ yes
- ◯ no

Date deposited in EMBL-EBI EGA ______________________ (YYYY-MM-DD)

Specify which archive (all that apply)
- ☐ EMBL-EBI EGA
- ☐ RD-Connect
- ☐ Other

If "Other", add details here (location & date archived) ______________________

ICGNMD Data Use

Participant's data used in ICGNMD publication?
- ◯ yes
- ◯ no

Enter DOI for publication(s) here

____

Participant links to other studies

Is participant also recruited to another study (e.g. SOLVE-RD)?
- ◯ yes
- ◯ no
- ◯ don't know

Details of other studies participant enrolled in

____

Non-ICGNMD publications

Has participant been included in publications outside of ICGNMD (using non-ICGNMD data)?
- ◯ yes
- ◯ no

Enter DOI for non-ICGNMD-related publication(s) here

____

Final summary: relatives of proband

Has ICGNMD also provided findings for affected relatives?
- ◯ yes
- ◯ no

(Do NOT put details about relatives findings here - use relatives' own records)

How many affected relatives received their own ICGNMD research findings? (1-10)

____

List ICGNMD Study IDs of affected relatives with ICGNMD findings here

____
(Format IC_ABC_NNNNN, IC_ABC_NNNNN etc)
